# Macroevolution of Steep Interspecific Metabolic Allometry in an Old Insect Order

**DOI:** 10.1111/ele.70399

**Published:** 2026-05-17

**Authors:** Daniel Schönberger, Moa Metz, Masahito Tsuboi, Giuseppe Bianco, Zachary C. DeVries, Andreas Nord, Erik I. Svensson

**Affiliations:** ^1^ Department of Entomology University of Kentucky Lexington Kentucky USA; ^2^ Department of Biology Lund University Lund Sweden; ^3^ Department of Biology Norwegian University of Science and Technology Trondheim Norway

**Keywords:** allometry, bayou, evolvability, insects, macroevolution, metabolism, natural selection, phylogenetic comparative methods, phylogenetic path analysis, phytools

## Abstract

Endotherms exhibit metabolic allometric slopes of 0.75, which were long viewed as evolutionary constraints but which are increasingly questioned. In insects, allometric relationships between body size and metabolism are understudied. We used comparative phylogenetic methods to investigate macroevolution of metabolic allometry in Odonata (dragonflies and damselflies), an insect group with a unique ecology and dramatic macroevolution of body size. We found an interspecific allometric slope close to one (isometry). Metabolic rates evolved gradually with a weak pullback force and multiple shifts in metabolic optima, body size and flight behaviour. Cell size, nucleus size and body surface area did not explain this steep allometric slope. Instead, metabolic isometry likely reflects the high aerobic demands of flight and evolutionary increases in body size via cell numbers rather than cell size. Our results challenge traditional allometric theories and suggest that metabolic allometries can and do evolve, with far‐reaching implications for insect size evolution.

## Graphical abstract

We investigated the macroevolution of metabolic allometry in an old insect order (Odonata: dragonflies and damselflies) to test the hypothesis that metabolic scaling exponents reflect evolutionary constraints and a general slope of 0.75 on a log–log scale. In contrast to this constraint hypothesis, we found a steeper slope of 1.0 (metabolic isometry) for these highly aerobic insects, suggesting that such a universal constraint on the evolution of metabolic allometry is unlikely.

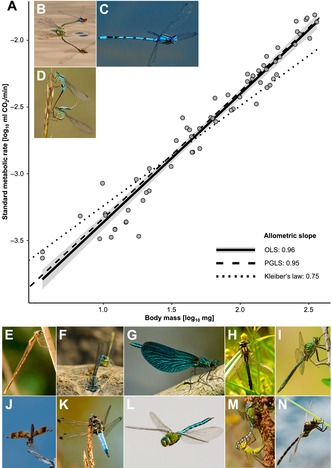

## 1 Introduction

The coevolution of size, shape and growth has been intensively discussed since Huxley and Teissier (1936). Allometry describes correlated growth of body size and other traits and links ontogeny, phylogeny and evolution (Gould 1966). Numerous traits such as brain size (Grabowski et al. 2023; Tsuboi et al. 2018), bird beaks (Rombaut et al. 2022), metabolic rates (Kleiber 1961; West et al. 1997; White et al. 2019) and life‐history characteristics (White et al. 2022) often show strong allometric relationships (i.e., high correlations) with body size. These allometric relationships typically have slopes less than one (Peters 1986). Isometry refers to the special case when the allometric slope is one. Tight allometric slopes indicate that much of the variation in trait size can be attributed to body size (Gould 1966; Huxley and Teissier 1936).

The mechanistic basis of allometric slopes and their ecological and evolutionary consequences have been extensively debated (Bolstad et al. 2015; Gould 1966, 1974; Pélabon et al. 2014; Voje and Hansen 2013). The allometric slope of metabolic rates is typically 0.75 across birds and mammals (Kleiber 1961; West et al. 1997). Such tight allometric slopes were historically interpreted as reflecting evolutionary constraints and low evolvability (Gould 1966). However, this slope of 0.75 is not shared across all taxonomic groups (Hatton et al. 2019).

The traditional view of allometric slopes as universal constraints has been increasingly questioned (Bokma 2004; Kozłowski and Konarzewsi 2004; Kozłowski and Weiner 1997). Experimental (Bolstad et al. 2015) and comparative studies (Grabowski et al. 2023; Tsuboi et al. 2018; Uyeda et al. 2017) have shown that allometric slopes can evolve (Bokma 2004; Kozłowski and Konarzewsi 2004; Kozłowski and Weiner 1997), albeit slowly and such changes are often only detectable over longer macroevolutionary time scales (Voje et al. 2014; Voje and Hansen 2013). However, Bolstad et al. (2015) used artificial selection on *Drosophila melanogaster* to change the allometric slope of wing shape, providing direct experimental evidence of evolvability of allometry. Furthermore, comparative studies (Grabowski et al. 2023; Tsuboi et al. 2018; Uyeda et al. 2017; White et al. 2022) indicate that allometric slopes reflect functional optimization and concerted evolution between body size and other traits (Arnqvist et al. 2022; Kozłowski and Weiner 1997) rather than only being evolutionary constraints. Concerted evolution could result from correlational selection on trait combinations (Svensson et al. 2021) that could shape genetic covariances between body size and metabolic rates and maintain adaptive allometric slopes (White et al. 2019).

The evolution of metabolic allometry is a major focus in ecology and evolutionary biology, with competing mechanistic theories predicting different allometric slopes (Glazier 2005, 2018, 2022b; Kozłowski et al. 2020; White et al. 2019, 2022). The Metabolic Theory of Ecology predicts a universal allometric slope of 0.75 based on nutrient supply networks (Brown et al. 2004; West et al. 1997). In contrast, another mechanistic theory grounded in physics and heat exchange principles predicts a slope of 0.67 because of greater surface‐to‐volume ratios of smaller animals (Dodds et al. 2001; Rubner 1883).

A third class of mechanistic hypotheses focuses on the links between cell size, nucleus size, genome size and metabolic rates (Gardner et al. 2020; Glazier 2022a; Hughes 1999; Kozłowski et al. 2003; Maciak et al. 2014; Schramm et al. 2021; Starostová et al. 2005, 2009). Since genome sizes are correlated with cell sizes, the small genome and cell sizes of birds and bats have been suggested to cause higher metabolic rates through larger cell surface area‐to‐volume ratios, providing respiratory advantages for these flying animals (Organ et al. 2007). Differences in cell size relative to cell numbers have also been proposed to affect the allometric slope (Glazier 2022a; Kozłowski et al. 2003; Maciak et al. 2014; Schramm et al. 2021). Specifically, metabolic allometries could evolve as a by‐product of body size optimization via an increased number of cells, and small cells have larger mass‐independent metabolic rates than large cells due to higher surface‐to‐volume ratios (Glazier 2022a; Kozłowski et al. 2003). Under this view, when body size increases via more cells we should expect isometric allometry (slope: 1.0), whereas increases via larger cells should produce a 0.67‐slope (Kozłowski et al. 2003). However, these proposed causal links between genome size, cell size, and flight as explanations of metabolic rate variation have been highly debated (Gardner et al. 2020; Starostová et al. 2009).

Macroevolutionary studies have revealed considerable variation in the metabolic allometric slope across clades (Glazier 2005, 2014, 2022b). Furthermore, metabolic rates can influence individual fitness and be targets of context‐dependent selection (Pettersen et al. 2016, 2020). This could imply that metabolic allometric slopes could evolve (Glazier 2005; White and Seymour 2005). Variation in metabolic allometries could reflect differences in physiology (Kozłowski and Weiner 1997; Maciak et al. 2014; Pettersen et al. 2016, 2020) and ecology (Glazier 2022b; Uyeda et al. 2017).

Due to their species richness and ecological diversity (Wilson 1999), insects should be of central importance in research on metabolic allometry (Ehnes et al. 2011; Gardner et al. 2020; Glazier 2022b; Riveros and Enquist 2011). However, few researchers have investigated metabolic allometry in insects (Chown et al. 2007; Ehnes et al. 2011; Gudowska et al. 2017; Riveros and Enquist 2011). Currently, metabolic rates and body mass data are only available for a few hundred insect species (Herberstein et al. 2022). Moreover, most studies have neglected measurement error (Brown et al. 2004; Glazier 2005; West et al. 1997), and few have used modern phylogenetic comparative methods (Chown et al. 2007; Uyeda et al. 2017; White et al. 2009, 2019). This is concerning, as phylogenetic relatedness and within‐species variation can significantly affect the estimates of allometric slopes (Ives et al. 2007; Tsuboi et al. 2018; White et al. 2009).

Here, we use phylogenetic comparative methods to study the macroevolution of metabolic allometry in the old insect order Odonata (dragonflies and damselflies), testing predictions for the different allometric slopes of 0.67, 0.75 and 1.0, from by the three theoretical frameworks introduced above. These insects are highly dependent on flight (Corbet 1999; Henry and Harrison 2014) and have a dramatic macroevolutionary history of body size, with extinct species being the largest known flying insects (Clapham and Karr 2012; Waller and Svensson 2017). These aerial insects can therefore shed light on the evolution of metabolic allometry in general, since insect flight is metabolically costly (Reinhold 1999). Our study is the largest on metabolic allometry in any insect group, covering 63 Odonata species from 654 individuals from Europe, North and Central America, 237 million years of macroevolution, and two orders of magnitude of body size variation.

We had three aims in this study. First, we estimated the metabolic allometric slope across Odonata, accounting for phylogeny and measurement errors. Next, we evaluated the support for the three different classes of theories that may explain allometric slopes. Specifically, we evaluated the effects of body surface area, body volume, cell size, nucleus size and flight behaviour on metabolic rates. Finally, we explored the macroevolutionary dynamics of metabolic allometry across the phylogeny of Odonata, and testing whether metabolic rates or the allometric slope have shifted over evolutionary history.

## 2 Results

### 2.1 Near‐Isometric Metabolic Slope

**FIGURE 1:**
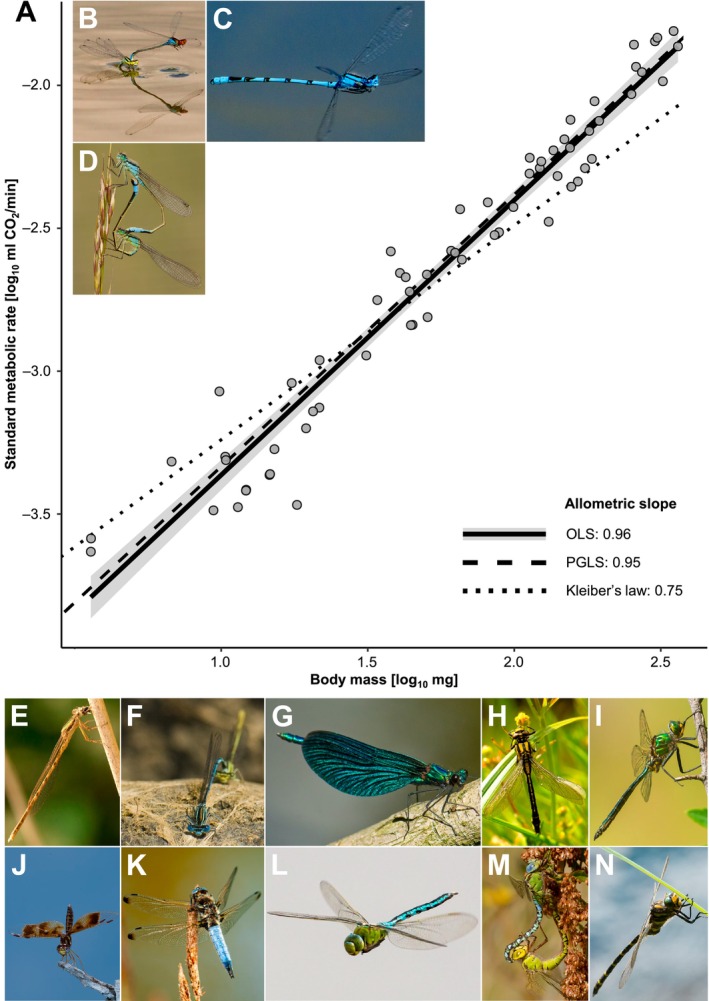
Interspecific metabolic allometry for 63 Odonata species across nine families and 30 genera. (A) Each point represents the species mean of log_10_ standard metabolic rate (SMR) and log_10_ body mass. SMR was strongly correlated with body mass (phylogenetically‐corrected correlation coefficient: 0.89). The allometric slope was close to one and significantly steeper than the expected slope of 0.75 based on Kleiber (1961) (dotted line). The solid regression line was fitted using ordinary least squares (OLS) (slope: 0.96 ± 0.03 SE, intercept: −4.33 ± 0.11 SE), with the 95% confidence interval shown as grey shading. The dashed line depicts the allometric regression line after accounting for phylogenetic relatedness and within‐species variances based on PGLS_IVES_ (Ives et al. 2007) (phylogenetic generalized least squares; slope: 0.95 ± 0.05 SE, intercept: −4.29 ± 0.09 SE). (B–N) Representative species from the nine families used in our study. Coenagrionidae: (B) *Erythromma viridulum*, (C) *Enallagma cyathigerum* , (D) *Ischnura elegans*. Lestidae: (E) *Sympecma fusca*. Platycnemididae: (F) *Platycnemis pennipes*. Calopterygidae: (G) *Calopteryx virgo*. Gomphidae: (H) *Gomphus vulgatissimus*. Corduliidae: (I) *Somatochlora metallica*. Libellulidae: (J) *Perithemis tenera* , (K) *Libellula fulva*. Aeshnidae: (L) *Anax imperator*, (M) *Aeshna viridis*. Cordulegastridae: (N) *Cordulegaster boltonii*. All photographs by E. I. Svensson.

We measured and compiled data on standard metabolic rate (SMR), flight behaviour, body mass, volume, surface area, cell size and nucleus size (see Section 4 and Supporting Information). When using species means and ignoring phylogenetic relatedness and within‐species variance, we found a strong positive relationship for metabolic allometry across Odonata (Figure 1A). On a log_10_‐scale, SMR was strongly correlated with body mass (*r* = 0.97), with an allometric slope of 0.96 (95% CI: 0.91–1.02). This allometric slope is considerably higher than traditionally expected (0.67/0.75) and is statistically indistinguishable from one (isometry).

Estimating trait relationships, such as metabolic allometries, among species requires accounting for phylogenetic relatedness among taxa (Revell and Harmon 2022). Hence, we used phylogenetic generalized least squares (PGLS) models with different branch‐length transformations to account for phylogenetic relatedness among taxa. After accounting for phylogeny, the correlation between SMR and body mass was high (phylogenetically‐corrected correlation coefficient: 0.89), and the allometric slope was still significantly higher than 0.67 and 0.75, and marginally below one (slope: 0.87, CI: 0.77–0.98) (Table S1).

Next, we tested if closely related species have similar metabolic rates by estimating the phylogenetic signals (*λ*) for absolute and mass‐independent SMR (Revell and Harmon 2022). Mass‐independent SMR was calculated using residuals obtained from phylogenetic size‐correction via PGSL_λ_ of SMR on body mass. Phylogenetic signals for absolute and mass‐independent SMR were both high (*λ* ≥ 0.98), revealing high phenotypic similarity between closely related taxa.

Within‐species variation has often been ignored in phylogenetic comparative studies but can cause a substantial downward bias of the estimated allometric slope (Garamszegi 2014; Ives et al. 2007). After correcting for within‐species variation (PGLS_IVES_), we recovered a near‐isometric slope that was not significantly different from one (slope: 0.96, CI: 0.86–1.05). This remained robust when incorporating within‐species sample sizes (Tables S1–S3). An additional source of attenuation bias is measurement error. By estimating the repeatability of SMR and body mass across species, we revealed high repeatability (0.88 and 0.96, respectively). Using the repeatability of body mass as a benchmark of attenuation bias leads to a corrected slope of 0.99.

Metabolic rates can show curvilinear relationships with body mass due to physiological limits at body mass extremes (Dodds et al. 2001; Kolokotrones et al. 2010). We found some evidence for curvilinearity indicated by a marginally better fit of a curvilinear PGLS_λ_ compared to a linear PGLS_λ_ and a positive quadratic coefficient (Table S4). Thus, the allometric slope might steepen with increasing body size in Odonata.

**FIGURE 2:**
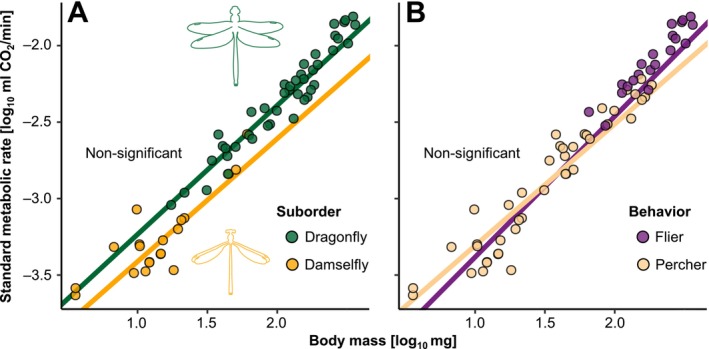
Comparison of interspecific metabolic allometry between suborders (A) and flight behaviours (B) across 63 Odonata species. There was no evidence for any significant difference in slope or intercept between the suborders (slope: *T* = −0.50, *p* = 0.62; intercept: *T* = −0.35, *p* = 0.72) or flight behaviours (slope: *T* = 0.62, *p* = 0.54; intercept: *T* = −0.84, *p* = 0.41). Each dot represents a species mean. Lines were fitted using phylogenetic analysis of covariance (Revell and Harmon 2022) with suborder or behaviour as covariates, including the interaction. Note that the apparent visual difference in intercept in panel A is not statistically significant and the confidence intervals around the intercept estimates are broad (intercept estimate for dragonflies = −4.08 ± 0.21; intercept estimate for damselflies = −4.22 ± 0.27).

Two ecological contrasts within Odonata are suborder (dragonflies and damselflies) and flight behavioural mode (fliers vs. perchers). The suborders differ profoundly in body size, ecology and life cycle lengths, whereas flight behavioural modes differ in morphology, physiology and ecology (Corbet 1999; May 1979). Thus, we expected different metabolic allometric slopes between suborder, behavioural modes or both. However, we found no differences in either allometric slope or intercept between suborders or flight behaviours (Figure 2). We also searched for outliers in metabolic allometry among Odonata families using phylogenetic analyses of covariance (Smaers and Rohlf 2016). We found that Coenagrionidae (pond damselflies) deviated significantly from the common metabolic allometry with a lower slope and higher intercept, while Lestidae (spreadwings) tended to deviate with a higher slope and lower intercept (Table S5). Excluding these two families from the overall PGLS_Ives_ estimate resulted in a similar steep slope (1.05, CI: 0.94–1.16).

### 2.2 Morphological and Cellular Traits Do Not Explain Interspecific Variation in Metabolic Rates

We performed phylogenetic path analysis—an indirect approach to infer causality using the covariance structure of a set of traits, combined with model comparisons (van der Bijl 2018; von Hardenberg and Gonzalez‐Voyer 2012). This enabled us to disentangle the direct and indirect effects on SMR of three morphological (body mass, volume and surface area) and two cellular traits (cell and nucleus size). Two sets of models were analysed (Model set 1 and Model set 2). Each set contained nine causal path models, where some models were nested within each other (Figure S4).

**FIGURE 3:**
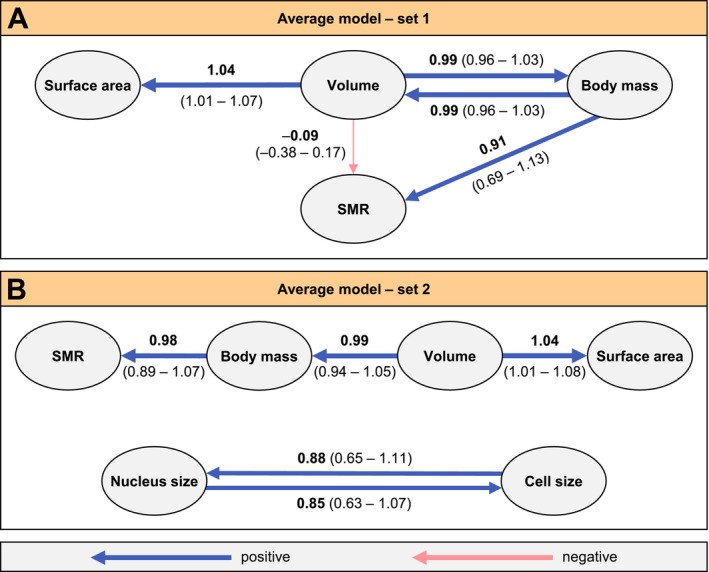
Average models from phylogenetic path analysis (Garamszegi 2014; von Hardenberg and Gonzalez‐Voyer 2012) based on the statistically supported models of set 1 (A) and set 2 (B). Only body mass (directly) and body volume (indirectly) influenced SMR, whereas cell size, nucleus size, and body surface area did not affect SMR. There was a strong reciprocal effect between body mass and body volume (A) and nucleus and cell size (B). Arrows in the directed acyclic graphs depict the direction of relationships between traits, with arrow widths representing relationship strength. Colours indicate negative (pink) or positive (blue) associations, and standardized path coefficients are provided with 95% confidence intervals in parentheses. For each model set, we used conditional model averaging to combine the supported models (ΔCIC ≤ 2) based on their model weight (Table S6, Figure S4; *n*_set1_ = 20 species, *n*_set2_ = 43 species) (Garamszegi 2014; von Hardenberg and Gonzalez‐Voyer 2012). Path models were fitted in the *phylopath R* package (van der Bijl 2018).

Model set 1 included the three morphological traits and had roughly twice the sample size of set 2. For set 1, we found strong statistical support for the models M7 and M2 (Table S6), where body mass but not surface area influenced SMR (Figure S4). These two supported models were combined into an average model that accounted for path uncertainty (Figure 3A). This average model showed a strong reciprocal effect between body mass and volume.

Model set 2 was based on the path structures of the statistically supported models of set 1, but included the two cellular traits in addition to the morphological traits in set 1 (Figure S4). For Model set 2, we found strong statistical support for models M1 and M2, whereas the remaining models fit poorly (Table S6). These best‐supported models revealed neither direct nor indirect effects of cell or nucleus size on SMR. Similarly, model structures with causal paths from cell or nucleus size to SMR, body mass, or volume lacked statistical support. Finally, we combined these two supported models by model averaging, leading to the best‐supported model (Figure 3B). In this average model, cell and nucleus size exhibited a strong correlation with each other, but neither influenced SMR (Figure 3B).

Overall, our phylogenetic path analysis (Figure 3) therefore strongly supported the positive link between SMR and body mass, with path coefficients close to one, which is consistent with metabolic isometry. The path analysis also revealed a strong positive effect of volume on body mass and surface area, whereas surface area did not affect SMR. Our results did not support any causal effect of cellular traits on metabolic rates.

### 2.3 Macroevolutionary Dynamics of Metabolic Rates

We investigated the tempo and mode of macroevolution of absolute and mass‐independent SMR and metabolic allometry and searched for multiple phenotypic optima. We evaluated several scenarios based on maximum likelihood (Revell and Harmon 2022) and Bayesian reversible‐jump Markov chain Monte Carlo (rjMCMC) models (Uyeda and Harmon 2014). Likelihood‐based models enabled the assessment of the relative role of suborders or flight behaviours by hypothesizing different adaptive regimes for dragonflies and damselflies or fliers and perchers mapped on the phylogeny (Figure S1). By contrast, rjMCMC searches for shifts in metabolic optima of an Ornstein‐Uhlenbeck (OU) process describing adaptive evolution without any predefined selective regimes (Uyeda and Harmon 2014).

In OU models (Hansen 1997), the phylogenetic half‐life describes the time it takes a lineage to evolve halfway towards a new trait optimum following a shift. Support for an OU over a Brownian Motion (BM) model combined with short phylogenetic half‐lives (shorter than tree height) indicates a pullback force towards trait optima. Long half‐lives (longer than tree height), or support for a BM over an OU model, are consistent with weak stabilizing selection and high phylogenetic inertia and could indicate neutral evolution. Short half‐lives towards multiple optima typically produce patterns of rapid evolutionary trait changes across a phylogenetic tree, while long half‐lives often lead to more gradual changes.

**FIGURE 4:**
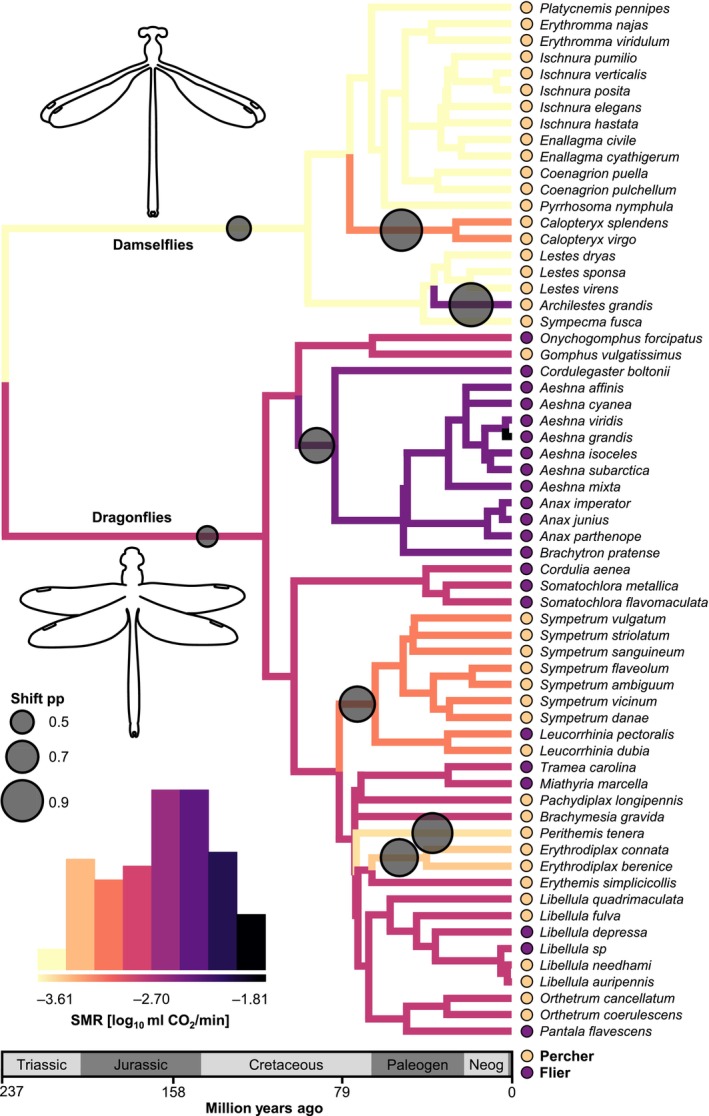
Macroevolution of standard metabolic rate (SMR) for perchers and fliers over 237 million years across the time‐calibrated Odonata phylogeny (63 species from nine families) based on a reversible‐jump Markov chain Monte Carlo analysis of a multi‐optima Ornstein‐Uhlenbeck model (Uyeda and Harmon 2014). Several optima shifts (circles) in SMR were identified across the phylogeny. Circle size on the phylogeny represents the posterior probability (pp) of a regime shift. Only shifts with pp ≥ 0.2 are shown. Colours of tree edges show the mean value of the trait optimum *θ* for that edge from the posterior. The histogram depicts the frequency of *θ* across the tree edges. Coloured circles at the tips represent flight behaviour (percher or flier). The analysis was performed and plotted in the *bayou R* package (Uyeda et al. 2020).

For absolute SMR, the maximum‐likelihood approach supported a multi‐optima Ornstein‐Uhlenbeck model with flight behaviour as an adaptive regime as the best fit (Table S7: _mo_OU behaviour). Fliers had a higher metabolic optimum than perchers (*θ*_Flier_: 8.13 ± 3.60 SE, *θ*_Percher_: −2.83 ± 0.21 SE). This model also had strong statistical support when restricting the analysis to dragonflies (ΔAIC_c_: 5.20). Large phylogenetic half‐life estimates of the _mo_OU behaviour model (*t*_1/2_ = 1256.5 MY, tree height: 237 MY) and rjMCMC analyses (Table S8) indicated only a weak pullback force on SMR. Combined with the high phylogenetic signal of SMR (see above), these results suggest gradual evolution without any rapid optima shifts (Figure S2). We also found support for an unbounded multi‐rate Brownian Motion (BM) model (Table S7: _mr_BM suborder), with SMR evolving twice as fast in damselflies (*σ* ^2^ = 0.0013) as in dragonflies (*σ* ^2^ = 0.00056). Furthermore, the rjMCMC approach identified multiple shifts for SMR optima across the phylogeny (*k* = 11, HPD: 7–15), suggesting that different clades have evolved towards higher or lower optima (Figure 4, Table S8).

**FIGURE 5:**
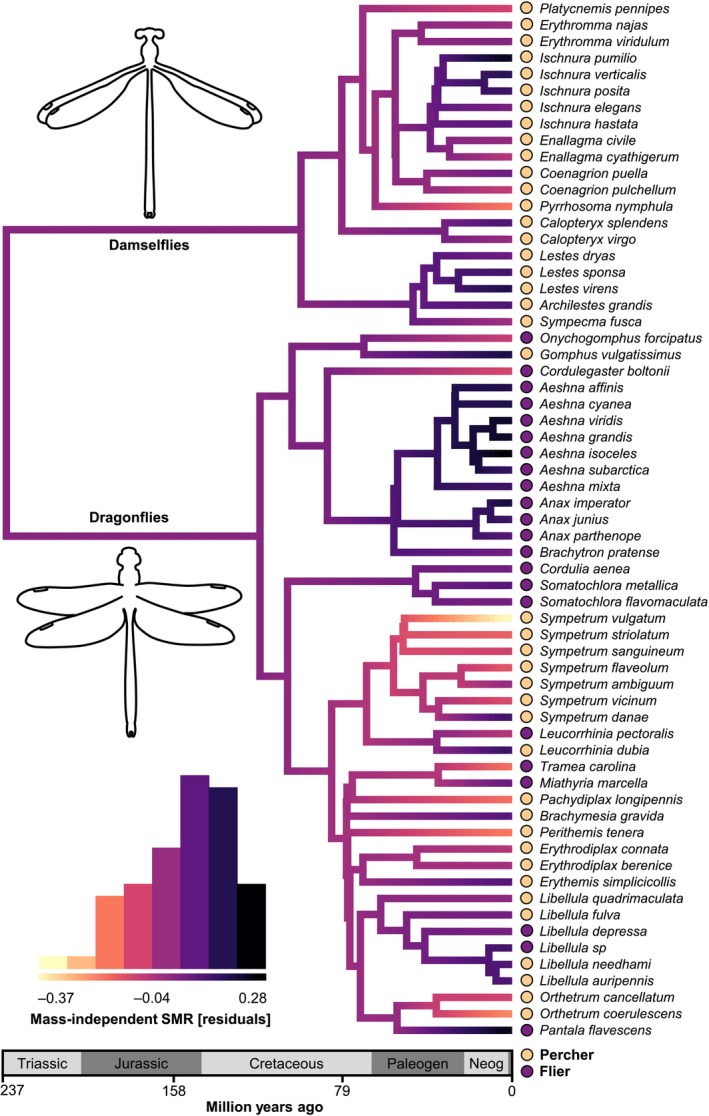
Macroevolution of mass‐independent standard metabolic rate (SMR) for perchers (tan circles) and fliers (purple circles) across the time‐calibrated Odonata phylogeny (63 species from nine families). Mass‐independent SMR evolved gradually and stayed remarkably constant over 237 million years, with only a few small clades or single species deviating from the common trend. The colour gradient shows the observed trait values at the tips and estimated values along the internal phylogeny. The histogram depicts the distribution of tip trait values. Mass‐independent SMR was estimated using the residuals obtained from phylogenetic size‐correction via generalized least squares regression of log_10_ SMR on log_10_ body mass. The tree was visualized based on a Brownian Motion model using the *contMap* function implemented in the *phytools R* package (Revell 2024).

Most metabolic optima shifts coincided with body size shifts, suggesting strongly concerted evolution between these two traits and that SMR might have evolved as a correlated response to selection on body mass (Figure 4, Figure S2). To investigate if and how metabolic rates could have evolved independently of body mass, we therefore next analysed mass‐independent SMR. Unlike absolute SMR, we found little evidence for variation in the macroevolutionary tempo and mode for mass‐independent SMR (Figure 5). The multi‐optima OU and multi‐rate BM maximum‐likelihood models had equally good fits in relation to flight behaviour and suborder, and both models were statistically indistinguishable from single‐optimum OU and single‐rate BM models (Table S7). The rjMCMC analysis for mass‐independent SMR detected several phenotypic shifts (*k* = 10, HPD: 5–16), but all had poor statistical support (Table S8). These results suggest a gradual evolution of mass‐independent metabolic rates, weak pullback forces towards optima and no major shifts. Although most taxa were close to the median mass‐independent SMR, a few species and small clades deviated from this evolutionary trend (Figure 5, Figure S3). In particular, the fast‐flying dragonfly families Aeshnidae (hawkers), Cordulegastridae (spiketails) and Corduliidae (emeralds) showed remarkably high metabolic rates (Figure S2), even after correcting for body mass (Figure 5). Their estimated metabolic optima differed from other clades (Figure 4).

**FIGURE 6:**
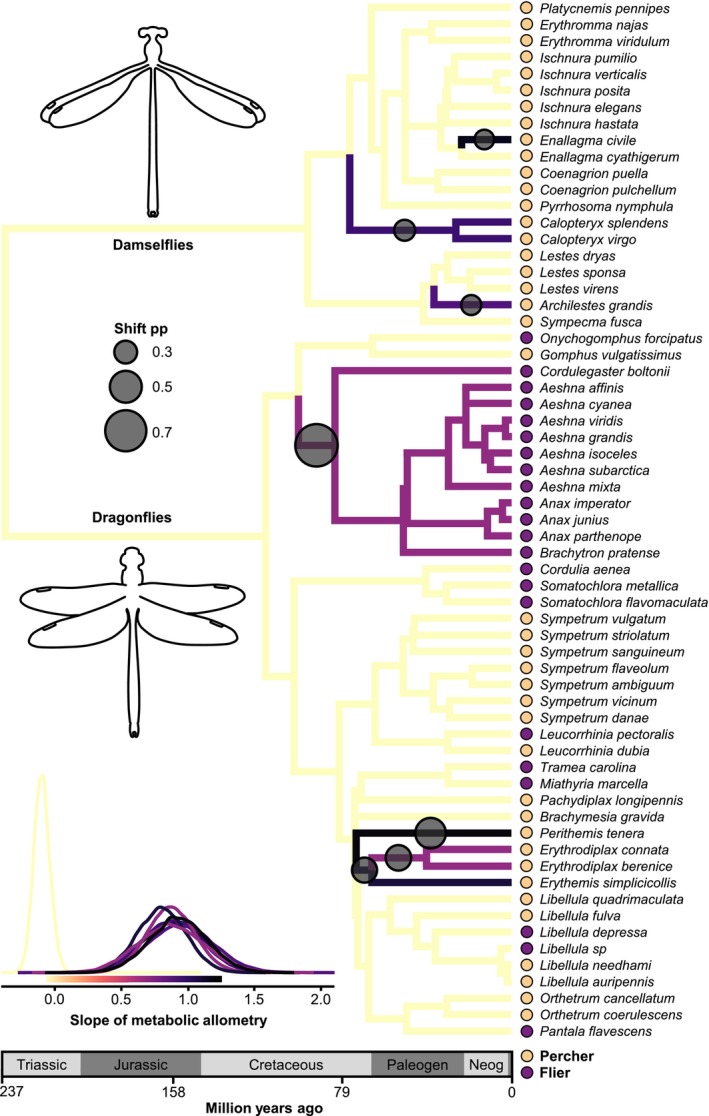
Macroevolutionary dynamics of the slope of the metabolic allometry for perchers and fliers across the time‐calibrated Odonata phylogeny (63 species from nine families) based on a reversible‐jump Markov chain Monte Carlo analysis of a multi‐optima Ornstein‐Uhlenbeck model (Uyeda and Harmon 2014). Several optima shifts (circles) in the allometric slope were identified across the phylogeny. Circle size on the phylogeny represents the posterior probability (pp) of a regime shift. Only shifts with pp ≥ 0.2 are shown. Colours of tree edges show the mean value of the trait optimum for that edge from the posterior (scale shown in density plot). The density plot depicts the posterior distribution of slope optima for the root state and divergent regimes (colours correspond to tree edge colours). Coloured circles at the tips indicate flight behaviour (percher or flier). The analysis was performed and plotted in the *bayou R* package (Uyeda et al. 2020).

Finally, we searched for shifts in metabolic allometry based on a multi‐optima OU model in an rjMCMC framework (Uyeda et al. 2017). This model suggested several clade‐specific optima shifts (*k* = 6.5, HPD: 2–10) and a gradual evolution of metabolic allometry over hundreds of millions of years, with weak pullback forces towards optima (high phylogenetic half‐lives) (Figure 6, Figure S11; Table S8). The inferred slope at the phylogenetic root was low, while all shifts towards clades with divergent optima were associated with increasing and near‐isometric slopes. Although most shifts led to single taxa or small clades, one major shift was found in the clade containing hawkers and spiketails, suggesting that these fast‐flying dragonflies have evolved towards a particularly steep allometric slope. Overall, the rjMCMC therefore indicates that the metabolic allometric slope has evolved from a relatively low ancestral optimum towards near isometry across the phylogeny. The cause behind the relatively shallow slope optimum inferred for the root remains an open question. In summary, SMR and allometric slopes seem to have evolved without rapid shifts and with weak pullback forces towards clade‐specific optima (Grabowski et al. 2023) and with metabolic rates being tightly correlated with body mass.

## 3 Discussion

Here, we investigated the evolution of metabolic allometry in dragonflies and damselflies using multiple phylogenetic comparative approaches. We evaluated three mechanistic sets of theories that predict different allometric slopes, namely 0.67, 0.75 and 1.00. Across this diverse insect order, there is a steep, nearly isometric metabolic allometric slope (Figure 1). Our results differ from the comparative study of metabolic allometry by Ehnes et al. (2011), which reported an allometric slope of 0.76 across 415 insect species. However, several insect orders deviate from this overall slope near 0.75 (Riveros and Enquist 2011).

Our results add to growing evidence that metabolic allometric slopes are not fixed constraints (Bokma 2004; Glazier 2005, 2018, 2022b; Hatton et al. 2019; Kozłowski and Konarzewsi 2004) but can evolve, albeit slowly (White et al. 2022). These results underscore the need to broaden the scope of research on metabolic allometries to encompass more insects and other invertebrates. Given their high taxonomic and ecological diversity (Wilson 1999), future research on insects and other small ectothermic invertebrates should advance our understanding of the evolution of metabolic allometry.

The steep metabolic allometry in Odonata enables us to reject both the constraint model based on the surface area‐to‐volume relationship (0.67‐slope) (Dodds et al. 2001; Rubner 1883) and the Metabolic Theory of Ecology, which is based on nutrient supply networks (0.75‐slope) (Brown et al. 2004; West et al. 1997). The former model based on the surface area‐to‐volume relationship predicts a slope of 0.67 and assumes isometrically shaped animals (Dodds et al. 2001; Rubner 1883). Although odonates rely on heat regulation via their body surface (May 1979), we found no support for any effects of surface area on SMR (Figure 3). Instead, the metabolic allometry showed a steep slope near one (isometry), contradicting these two classical constraint models with predicted slopes of 0.67 and 0.75, respectively.

The best predictor of SMR was body mass, suggesting strong co‐adaptation between metabolic rates and body mass (Arnqvist et al. 2022; Kozłowski and Weiner 1997; White et al. 2022). The steep allometric slope in Odonata did not differ statistically between the two suborders (dragonflies and damselflies), although both differ markedly in morphology, behaviour and ecology (Corbet 1999). Furthermore, we found no significant difference between fliers and perchers in metabolic slopes (May 1979). A metabolic difference between fliers and perchers has been predicted because of their different thermoregulatory strategies, with species spending more time flying predicted to have higher metabolic rates to facilitate flight respiration (Corbet 1999; May 1979). Although we found no overall metabolic difference between these flight types, we found evidence for multiple optima among some of the fast‐flying dragonfly families (Figures 4, 5, 6).

We found no evidence for cell size or nucleus size influencing SMR (Figure 3). The lack of detectable effects of cell and nucleus size on SMR is surprising, given strong correlations between cell, nucleus and genome size (Malerba and Marshall 2021) and given previous studies on birds and mammals suggesting nucleotypic effects on metabolic rates in some vertebrate groups. We note that our analysis was limited to hemolymph cells and we did not include other metabolically active cell types. While a strong correlation between nucleus and cell size is prevalent across Odonata (this study) and other organisms (Malerba and Marshall 2021), any relationship between genome size and metabolic rates is likely to be mediated by cell size (Starostová et al. 2005). Research on birds, bats and pterosaurs has revealed repeated evolution of smaller genomes in flying lineages compared to flightless relatives (Hughes 1999; Organ et al. 2007). Birds with high flight capacity have smaller genomes, while flightless birds have larger genomes than closely related flying relatives (Hughes 1999). Small genomes and small cells have been suggested to facilitate avian flight due to increased metabolic efficiency (Hughes 1999). Such nucleotypic effects are expected because of the tight correlations between genome, nucleus and cell size, with small cells having high metabolic capacity because of high surface area‐to‐volume ratios (Gardner et al. 2020; Maciak et al. 2014).

These results are consistent with the cell number model for the evolution of body size in Odonata. This model proposes that metabolic allometric slopes are by‐products of body size optimization and arise through changes in cell number. Body size increases via increasing cell numbers are expected to yield isometric slopes, whereas increases via larger cell size should yield a slope of 0.67. Consistent with this hypothesis (Chown et al. 2007; Glazier 2022a; Kozłowski et al. 2003; Schramm et al. 2021), we found a near‐isometric allometric slope without effects of cell size on body mass. These results suggest that body size in Odonata increases primarily via more rather than larger cells, a pattern common in animals (Glazier 2005). Future studies on other insect groups that quantify cell numbers across a range of body sizes could provide further support for this hypothesis.

Alternatives to constraint models of metabolic allometries are adaptive models based on natural selection and life‐history theory. Kozłowski and Weiner (1997) argued that interspecific allometries are by‐products of intraspecific optimization of body size and life‐history traits. According to this view, interspecific metabolic allometries are non‐causal emergent epiphenomena arising from the underlying life‐history evolution at the intraspecific level. Thus, body size and metabolic rates are likely to be co‐adapted traits rather than metabolic allometries or simply reflecting physical, physiological, or genetic constraints (Arnqvist et al. 2022; White et al. 2022). Rather than evolving solely as a correlated response to selection on body size (Peters 1986), metabolic rates have been shown to affect fitness and are sometimes direct targets of selection (Pettersen et al. 2016, 2020). Metabolic rates and body size could therefore become co‐adapted by correlational selection (Svensson et al. 2021; White et al. 2019).

We found several clade‐specific optima for the metabolic allometry, suggestive of adaptive evolutionary trends towards different optima (Grabowski et al. 2023; Hansen 1997). Although the rate of adaptation was low (high phylogenetic half‐lives) and slower than for metabolic allometry in vertebrates (Uyeda et al. 2017), our results suggest that a single shared optimum for metabolic allometry is unlikely. One Odonata family was a significant outlier from the overall allometry, while another tended to deviate. The gradual evolution of metabolic allometry rates in Odonata over the last 237 MY suggests that large deviations from the overall trend are rare. Our results contradict the hypothesis that correlational selection maintains a global slope of 0.75 across all animal groups (White et al. 2019). Instead, our results suggest a common and shared allometric slope without major shifts across Odonata, but with adaptive evolution of allometric slopes still possible within this narrow ‘phenotypic corridor’, as indicated by some clades deviating from the overall trend across this order.

The high phylogenetic signals for metabolic rates and the strong correlation between metabolic rate and body size and between cell and nucleus size have implications for the macroevolutionary predictability and the prospects of reconstructing ancestral physiological and cellular phenotypes in Odonata. Such ancestral state reconstructions of metabolic phenotypes have been conducted for fossil and extant molluscs (Finnegan et al. 2011; Payne et al. 2014; Strotz et al. 2018). Since the body sizes of molluscs (Strotz et al. 2018) and Odonata (this study) are so strongly correlated with metabolic rates, it is possible to reliably infer metabolic rates from measurements of body sizes alone. This opens research avenues for macroevolutionary studies on physiology, especially given the rich fossil records of both groups (Finnegan et al. 2011; Grimaldi and Engel 2005; Payne et al. 2014). Paleontological data indicate that metabolic rates of extinct mollusc taxa were higher than for extant taxa, but species assemblages remained energetically stable throughout many extinctions and climate changes (Strotz et al. 2018).

These high phylogenetic signals are noteworthy, considering that physiological traits are often labile and plastic (Garamszegi 2014). Interestingly, high phylogenetic signals were previously found for another plastic trait in Odonata linked to physiology: temperature‐dependent phenological reaction norms (De Lisle et al. 2022). This reliability is supported by high cross‐validation accuracy (Supporting Information). However, we detected a weak curvature in the allometric relationship (Table S4), so predictions are less reliable for very small or large Odonata. We further note that we measured the metabolic rates of resting animals, rather than active metabolic rates during flight. Nevertheless, SMR and maximum metabolic rates are usually strongly correlated, and increased SMR might have evolved as a correlated response to selection on active or maximal metabolic performance during energetically demanding behaviours (Bennet and Ruben 1979; Reinhold 1999).

The steep metabolic allometry in Odonata has implications for their dramatic body size evolution (Clapham and Karr 2012; Svensson et al. 2023; Waller and Svensson 2017). Ancestors of Odonata include the extinct large‐bodied genus *Meganeura* from the Carboniferous (Grimaldi and Engel 2005). The large size of these extinct insects has been attributed to higher atmospheric oxygen levels in the Carboniferous and their dependence on trachea for oxygen uptake (Grimaldi and Engel 2005). Atmospheric oxygen levels later declined (Clapham and Karr 2012) but body size evolution has been slow over the last 120 MY despite fitness benefits of large size (Waller and Svensson 2017). Explanations for this slow evolution include conflicting selection between life stages (Waller and Svensson 2017), falling atmospheric oxygen levels and increased predation on larger species following the origin of birds (Clapham and Karr 2012), and a combination of temperature, predation risk and size‐dependent dispersal (Svensson et al. 2023).

We suggest that decreasing prey availability and high energetic demands of large‐bodied Odonata species may have contributed to their extinction. Because of near‐isometric metabolic allometry, large species expend similar amounts of energy as small species per unit body mass. Although these insects are highly efficient predators, their success rate decreases when prey availability declines (Combes et al. 2012). Declines in insect prey would probably affect larger Odonata more than smaller species due to their high energetic demands compared to other animals (Henry and Harrison 2014). These higher demands make large Odonata particularly susceptible to prey abundance declines and could elevate their extinction risk (Suárez‐Tovar et al. 2019).

Recent research has shown that allometries are not fixed constraints but can and do evolve (Bolstad et al. 2015; Tsuboi et al. 2018; Uyeda et al. 2017). Our study adds to the growing evidence that allometric relationships have evolved, rather than there being a single universal allometric slope across the entire Tree of Life (Bokma 2004; Kozłowski and Konarzewsi 2004). Our results underscore the need to test multiple hypotheses (Glazier 2005, 2018, 2022a) using modern phylogenetic comparative methods (Garamszegi 2014; Revell and Harmon 2022) in research on metabolic allometries. Body mass and metabolic rates are tightly linked and have evolved in concert, possibly through selection for co‐adaptation of physiology, behaviour and life‐history traits (Arnqvist et al. 2022; White et al. 2022). The high metabolic demands (Moore et al. 2023), strong environmental effects on body size (Clapham and Karr 2012; Svensson et al. 2023; Waller and Svensson 2017), and selection for high foraging efficiency (Combes et al. 2012) presumably explain the evolution of the steep metabolic allometry in Odonata, with implications for other flying insects.

## 4 Methods

We captured 651 adults from 57 species in Sweden and the USA (2021–2023). We estimated SMR by measuring the rate of CO_2_ production via stop‐flow respirometry. We extracted additional published SMR and body mass data from 13 species from North America. In total, 654 measurements of 63 species were used. We used micro‐computed tomography to estimate the volume and surface area of specimens. We measured hemocyte cells from the hemolymph of field‐collected Odonata.

Statistical analyses used *R* v.4.3.1 (R Core Team 2021). We used linear models and phylogenetic comparative approaches (Garamszegi 2014; Revell 2024; Revell and Harmon 2022) to analyse interspecific variation in metabolic rate and its relation to body mass, volume, surface area, cell size, nucleus size and flight behaviour.

To investigate the macroevolution of SMR, we compared maximum‐likelihood models based on Brownian Motion or Ornstein‐Uhlenbeck processes, hypothesizing different selective regimes for suborders or flight behaviours (Hansen 1997; Revell and Harmon 2022). This hypothesis‐driven approach was complemented by reversible‐jump Markov chain Monte Carlo analyses to detect optima shifts in absolute and mass‐independent SMR and metabolic allometry (Uyeda et al. 2017, 2020; Uyeda and Harmon 2014).

A time‐calibrated phylogeny (Waller and Svensson 2017) was used for all phylogenetic analyses. Seven taxa were not included in the phylogeny, so they were replaced with closely‐related congeners. This did not affect our results (Tables S11 and S12).

Additional methodological details are provided in Supporting Information.

## Author Contributions

Conceived study: E.I.S. and A.N. Planned experiments: E.I.S., A.N., M.M. and D.S. Fieldwork: D.S., M.M. and E.I.S. Laboratory work: D.S. and M.M. (supervised by A.N. and Z.C.D.). Statistical analyses and figures: D.S. (supervised by E.I.S., A.N., M.T. and G.B.). First draft: D.S. (supervised by E.I.S.). Second draft: E.I.S. (with input from D.S., M.M., M.T., G.B., Z.C.D. and A.N.). Contribution to final manuscript: All authors.

## Funding

This work was financially supported by the Swedish Research Council (“Vetenskapsrådet”: grants no. 2020 03123 and 2024 03965 to E.I.S. and no. 2020 04686 to A.N.), “Sven och Lilly Lawskis Fond för Naturvetenskaplig Forskning”, to M.M. and “Lunds Djurskyddsfond” to E.I.S.

## Conflicts of Interest

The authors declare no conflicts of interest.

## Supporting information


**Figure S1:** Marginal ancestral state reconstruction and transition rates (Revell and Harmon 2022) for the flight behaviour of 67 Odonata species from nine families over 237 million years across the time‐calibrated phylogeny. Transition rates (shown in left bottom corner) from flier to percher were much higher than vice versa, with percher being the ancestral state. Reconstruction was performed by model weighting based on four different transition matrices and stochastic character mapping using the *fitMk* function and 701 simulations using the *simmap* function in the *phytools R* package (Revell 2024).
**Figure S2:** Macroevolution of standard metabolic rate (SMR) for fliers (tan circles) and perchers (purple circles) over 237 million years across the time‐calibrated Odonata phylogeny (63 species of nine families). SMR strongly varied across the phylogeny and evolved gradually and in concert with body mass and flight behaviour. The colour gradient shows the observed trait values at the tips and estimated values along the internal phylogeny. The histogram depicts the distribution of trait values at the tips. The tree was visualized using the *contMap* function in the *phytools R* package (Revell 2024), which creates maps based on a Brownian Motion model.
**Figure S3:** Macroevolution of mass‐independent standard metabolic rate (SMR) for fliers (tan tip circles) and perchers (purple tip circles) over 237 million years across the time‐calibrated Odonata phylogeny (63 species) based on a reversible‐jumb Markov chain Monte Carlo analysis of the multi‐regime Ornstein‐Uhlenbeck model (Uyeda and Harmon 2014). Evidence for regime shifts (circles on phylogeny) in mass‐independent SMR was limited since all detected shifts had low posterior probabilities (pp). The colours of the tree edges show the mean value of the trait optimum (*θ*) for that edge from the posterior sample, and the histogram depicts the frequency of *θ* across the tree edges. Only shifts with pp ≥ 0.2 are shown. We estimated mass‐independent SMR using the residuals obtained from phylogenetic size‐correction via generalized least squares regression of log_10_ SMR on log_10_ body mass. The analysis was performed and plotted in the *bayou R* package (Uyeda et al. 2020).
**Figure S4:** Directed acyclic graphs (DAG) of the alternative phylogenetic path models for model set 1 (A) and set 2 (B) (Garamszegi 2014; von Hardenberg and Gonzalez‐Voyer 2012). Each DAG depicts the hypothesized relationships among morphological and cellular traits (log_10_‐scale) across Odonata. Two sets with nine causal path models each were performed. Set 1 represents the relationship between standard metabolic rate and three morphological traits (body mass, body volume and body surface area) across 43 Odonata species. Statistically supported models of set 1 were retained and incorporated in set 2. Model set 2 additionally included two cellular traits (nucleus and cell size) and the morphological traits of model set 1 across 20 species. Statistically supported models (ΔCIC_c_ ≤ 2; Table S7) are highlighted in orange and were combined into an average model for each set (Figure 5). Path models were fitted in *phylopath* (van der Bijl 2018).
**Figure S5:** Map of collection sites for Odonata specimens collected in southern Sweden (regions of Skåne and Blekinge). Community sampling locations (rosa dots) were visited regularly over the field season, whereas targeted sampling localities (blue dots) were only visited once or a few times to aim for rare species. Black dots represent cities. Numbers depict locality ID (Table S9). The enlarged section of the map is depicted in the top left corner.
**Figure S6:** Body volume and body surface area reconstruction of the dragonfly and damselfly specimens in *Blender* based on prior micro‐computer tomography (μCT) scans. The reconstruction and the resultant remesh of a male common darter *Sympetrum striolatum* (CT ID: 226) are shown as an example. (A) Reconstruction after the successful μCT scan and removal of the noise, the wings, legs and antennae. (B) The reconstruction was then remeshed and manually adjusted to approximate the reconstructions. (C) The head was removed and the abdomen and thorax were separated, allowing the estimation of the surface area and volume of separately.
**Figure S7:** High‐resolution cell imaging using the Operetta CLS High‐Contend Analysis System and the integrated Harmony High‐Content Imaging and Analysis Software to estimate cell and nucleus sizes (area) using hemocytes in dragonflies and damselflies. This exemplary image shows hemocyte cells of the blue emperor Anax imperator (Anisoptera: Aeshnidae). Cells were stained using Anthraquinone dye DRAQ5TM.
**Figure S8:** There was a strong correlation (*r* = 0.99, *p* < 10^−16^) between log_10_ dry and log_10_ fresh mass across the Odonata order (53 species). Each green or orange dot represents an individual measurement (*n* = 626) of a dragonfly or damselfly, respectively. The solid line represents the fitted regression line of a linear model with log_10_ dry mass as response and log_10_ fresh mass as predictor variable (slope: 0.99 ± 0.005 standard error, intercept: −4.12 ± 0.01 standard error).
**Figure S9:** Comparison of body mass, standard metabolic rate (SMR), body surface area (SA), body volume, thorax SA, thorax volume, abdomen SA and abdomen volume between flight behaviours (top) and suborders (bottom) for 47 or 56 Odonata species (*n*). Large points and bars show estimates of the mean and 95% confidence intervals based on phylogenetic analysis of variance (pANOVA) models fitted with lambda estimation in the *phylolm R* package (Ho and Ane 2014). For all body traits, a log_10_‐transformation was conducted. For all body traits except body mass, we accounted for mass differences by including body mass as a covariate. ns indicates no evidence for a significant difference (*p* > 0.05) and asterisks a significant difference (*p* ≤ 0.05*, *p* ≤ 0.001***).
**Figure S10:** Overview of morphological data, comparing the two flight behaviours (left) and suborders (right). Untransformed data is shown (see Figure S9 for sample sizes).
**Figure S11:** Macroevolutionary dynamics of the slope of the metabolic allometry for perchers (tan tip circles) and fliers (purple tip circles) across the time‐calibrated Odonata phylogeny (63 species from nine families) based on a reversible‐jumb Markov chain Monte Carlo analysis of the multi‐optima Ornstein‐Uhlenbeck model (Uyeda and Harmon 2014). Several optima shifts (circles) in allometric slope were identified across the phylogeny, with evidence for weak constraining forces. Circle size on the phylogeny represents the posterior probability (pp) of regime shifts. Only shifts with pp. ≥ 0.2 are shown. Colours of tree edges show the mean value of the trait optimum for that edge from the posterior (scale shown in density plot). Density plot depicts the posterior distribution of slope optima for the root state and divergent regimes (colours correspond to tree edge colours). The analysis was performed and plotted in the *bayou R* package (Uyeda et al. 2020).
**Figure S12:** Interspecific metabolic allometry for 63 Odonata species across nine families and 30 genera. Similar to Figure 1 in the main text, but with the source of each species indicated by point colour.
**Table S1:** Phylogenetic generalized least squares (PGLS) models for the interspecific metabolic allometry of 63 Odonata species.
**Table S2:** Phylogenetic generalized least squares (PGLS) models for the interspecific metabolic allometry of 63 Odonata species using fresh mass instead of dry mass.
**Table S3:** Phylogenetic generalized least squares (PGLS) models for the interspecific metabolic allometry of 34 Odonata species after excluding species with sample sizes smaller than 10.
**Table S4:** Evidence for curvilinearity in metabolic allometry in Odonata (Kolokotrones et al. 2010).
**Table S5:** Phylogenetic analysis of covariance models (Smaers and Rohlf 2016) to detect Odonata families representing a significant outlier in the metabolic allometry.
**Table S6:** Results of phylogenetic path analyses (Garamszegi 2014; von Hardenberg and Gonzalez‐Voyer 2012) for models (set 1 and set 2).
**Table S8:** Results of the reversible‐jumb Markov chain Monte Carlo (rjMCMC) models based on multi‐optima Ornstein‐Uhlenbeck models (Uyeda et al. 2017; Uyeda and Harmon 2014).
**Table S9:** Collection localities in Sweden for the dragonfly and damselfly individuals used in our study.
**Table S10:** Overview of the 63 Odonata species used to study the metabolic allometry.
**Table S13:** Overview of the 20 Odonata species used to estimate the cell and nucleus sizes (area in μm^2^), including their respective family, suborder, flight behaviour and sample size (number of individuals).
**Table S14:** Overview of the 47 Odonata species used to estimate the body volume (mm^3^) and body surface area (mm^2^), including their respective family, suborder, flight behaviour and sample size (number of individuals).
**Table S15:** Output from regression models assuming different respiratory quotients for data conversion.

## Data Availability

Data accessibility statement: Original data and R code behind all analyses are uploaded on Zenodo (https://doi.org/10.5281/zenodo.19667267).

## References

[ele70399-bib-0001] Arnqvist, G. , J. Ronn , C. Watson , J. Goenaga , and E. Immonen . 2022. “Concerted Evolution of Metabolic Rate, Economics of Mating, Ecology, and Pace of Life Across Seed Beetles.” Proceedings of the National Academy of Sciences of the United States of America 119: 1–11. 10.1073/pnas.2205564119 PMC9388118 35943983

[ele70399-bib-0002] Bennet, A. F. , and J. A. Ruben . 1979. “Endothermy and Activity in Vertebrates.” Science 206: 649–654. 493968 10.1126/science.493968

[ele70399-bib-0003] Bokma, F. 2004. “Evidence Against Universal Metabolic Allometry.” Functional Ecology 18: 184–187.

[ele70399-bib-0004] Bolstad, G. H. , J. H. Cassara , E. Márquez , et al. 2015. “Complex Constraints on Allometry Revealed by Artificial Selection on the Wing of *Drosophila melanogaster* .” Proceedings of the National Academy of Sciences of the United States of America 112: 13284–13289. 26371319 10.1073/pnas.1505357112 PMC4629349

[ele70399-bib-0005] Brown, J. H. , J. F. Gillooly , A. P. Allen , M. van Savage , and G. B. West . 2004. “Toward a Metabolic Theory of Ecology.” Ecology 85: 1771–1789.

[ele70399-bib-0006] Chown, S. L. , E. Marais , J. S. Terblanche , C. J. Klok , J. R. B. Lighton , and T. M. Blackburn . 2007. “Scaling of Insect Metabolic Rate Is Inconsistent With the Nutrient Supply Network Model.” Functional Ecology 21: 282–290.

[ele70399-bib-0007] Clapham, M. E. , and J. A. Karr . 2012. “Environmental and Biotic Controls on the Evolutionary History of Insect Body Size.” Proceedings of the National Academy of Sciences of the United States of America 109: 10927–10930. 22665762 10.1073/pnas.1204026109 PMC3390886

[ele70399-bib-0008] Combes, S. A. , D. E. Rundle , J. M. Iwasaki , and J. D. Crall . 2012. “Linking Biomechanics and Ecology Through Predator–Prey Interactions: Flight Performance of Dragonflies and Their Prey.” Journal of Experimental Biology 215: 903–913. 22357584 10.1242/jeb.059394

[ele70399-bib-0009] Corbet, P. S. 1999. Dragonflies – Behaviour and Ecology of Odonata. Harley Books.

[ele70399-bib-0010] De Lisle, S. P. , M. I. Mäenpää , and E. I. Svensson . 2022. “Phenotypic Plasticity Is Aligned With Phenological Adaptation on Both Micro‐ and Macroevolutionary Timescales.” Ecology Letters 25: 790–801. 35026042 10.1111/ele.13953

[ele70399-bib-0011] Dodds, P. S. , D. H. Rothman , and J. S. Weitz . 2001. “Re‐Examination of the “3/4‐Law” of Metabolism.” Journal of Theoretical Biology 209: 9–27. 11237567 10.1006/jtbi.2000.2238

[ele70399-bib-0012] Ehnes, R. B. , B. C. Rall , and U. Brose . 2011. “Phylogenetic Grouping, Curvature and Metabolic Scaling in Terrestrial Invertebrates.” Ecology Letters 14: 993–1000. 21794052 10.1111/j.1461-0248.2011.01660.x

[ele70399-bib-0013] Finnegan, S. , C. M. McClain , M. A. Kosnik , and J. L. Payne . 2011. “Escargots Through Time: An Energetic Comparison of Marine Gastropod Assemblages Before and After the Mesozoic Marine Revolution.” Paleobiology 37: 252–269.

[ele70399-bib-0014] Garamszegi, L. Z. 2014. Modern Phylogenetic Comparative Methods and Their Application in Evolutionary Biology. Concepts and Practice. Springer.

[ele70399-bib-0015] Gardner, J. D. , M. Laurin , and C. L. Organ . 2020. “The Relationship Between Genome Size and Metabolic Rate in Extant Vertebrates.” Philosophical Transactions of the Royal Society B 375: 20190146. 10.1098/rstb.2019.0146 PMC7017434 31928192

[ele70399-bib-0016] Glazier, D. S. 2005. “Beyond the “3/4‐Power Law”: Variation in the Intra‐ and Interspecific Scaling of Metabolic Rate in Animals.” Biological Reviews of the Cambridge Philosophical Society 80: 611–662. 16221332 10.1017/S1464793105006834

[ele70399-bib-0017] Glazier, D. S. 2014. “Metabolic Scaling in Complex Living Systems.” System 2: 451–540.

[ele70399-bib-0018] Glazier, D. S. 2018. “Rediscovering and Reviving Old Observations and Explanations of Metabolic Scaling in Living Systems.” System 6: 4.

[ele70399-bib-0019] Glazier, D. S. 2022a. “How Metabolic Rate Relates to Cell Size.” Biology‐Basel 11: 1106. 35892962 10.3390/biology11081106 PMC9332559

[ele70399-bib-0020] Glazier, D. S. 2022b. “Variable Metabolic Scaling Breaks the Law: From “Newtonian” to “Darwinian” Approaches.” Proceedings of the Royal Society B: Biological Sciences 289: 20221605. 10.1098/rspb.2022.1605 PMC9579773 36259209

[ele70399-bib-0021] Gould, S. J. 1966. “Allometry and Size in Ontogeny and Phylogeny.” Biological Reviews 41: 587–638. 5342162 10.1111/j.1469-185x.1966.tb01624.x

[ele70399-bib-0022] Gould, S. J. 1974. “The Origin and Function of “Bizarre” Structures: Antler Size and Skull Size in the “Irish Elk” Megaloceros Giganteus.” Evolution (N Y) 28: 191–220. 10.1111/j.1558-5646.1974.tb00740.x 28563271

[ele70399-bib-0023] Grabowski, M. , B. T. Kopperud , M. Tsuboi , and T. F. Hansen . 2023. “Both Diet and Sociality Affect Primate Brain‐Size Evolution.” Systematic Biology 72: 404–418. 36454664 10.1093/sysbio/syac075 PMC10275546

[ele70399-bib-0024] Grimaldi, D. A. , and M. E. Engel . 2005. Evolution of the Insects. Cambridge University Press.

[ele70399-bib-0025] Gudowska, A. , B. W. Schramm , M. Czarnoleski , A. Antol , U. Bauchinger , and J. Kozlowski . 2017. “Mass Scaling of Metabolic Rates in Carabid Beetles (Carabidae)—The Importance of Phylogeny, Regression Models and Gas Exchange Patterns.” Journal of Experimental Biology 220: 3363–3371. 28724648 10.1242/jeb.159293

[ele70399-bib-0026] Hansen, T. F. 1997. “Stabilizing Selection and the Comparative Analysis of Adaptation.” Evolution (N Y) 51: 1341–1351. 10.1111/j.1558-5646.1997.tb01457.x 28568616

[ele70399-bib-0027] Hatton, I. A. , A. P. Dobson , D. Storch , E. D. Galbraith , and M. Loreau . 2019. “Linking Scaling Laws Across Eukaryotes.” Proceedings of the National Academy of Sciences of the United States of America 116: 21616–21622. 31591216 10.1073/pnas.1900492116 PMC6815163

[ele70399-bib-0028] Henry, J. R. , and J. F. Harrison . 2014. “Body Size Effects on the Oxygen‐Sensitivity of Dragonfly Flight.” Journal of Experimental Biology 217: 3447–3456. 25063859 10.1242/jeb.095828

[ele70399-bib-0029] Herberstein, M. E. , D. J. Mclean , E. Lowe , et al. 2022. “AnimalTraits—A Curated Animal Trait Database for Body Mass, Metabolic Rate and Brain Size.” Scientific Data 9: 265. 35654905 10.1038/s41597-022-01364-9 PMC9163144

[ele70399-bib-0079] Ho, L. S. T. , and C. Ane . 2014. “A Linear‐Time Algorithm for Gaussian and Non‐Gaussian Trait Evolution Models.” Systematic Biology 63: 397–408. 24500037 10.1093/sysbio/syu005

[ele70399-bib-0030] Hughes, A. L. 1999. “Adaptive Characteristics of Genomes.” In Adaptive Evolution of Genes and Genomes, 180–221. Oxford University Press.

[ele70399-bib-0031] Huxley, J. S. , and G. Teissier . 1936. “Terminology of Relative Growth.” Nature 137: 780–781.

[ele70399-bib-0032] Ives, A. R. , P. E. Midford , and T. Garland . 2007. “Within‐Species Variation and Measurement Error in Phylogenetic Comparative Methods.” Systematic Biology 56: 252–270. 17464881 10.1080/10635150701313830

[ele70399-bib-0033] Kleiber, M. 1961. The Fire of Life. An Introduction to Animal Energetics. John Wiley & Sons.

[ele70399-bib-0034] Kolokotrones, T. , V. Savage , E. J. Deeds , and W. Fontana . 2010. “Curvature in Metabolic Scaling.” Nature 464: 753–756. 20360740 10.1038/nature08920

[ele70399-bib-0035] Kozłowski, J. , and M. Konarzewsi . 2004. “Is West, Brown and Enquist's Model of Allometric Scaling Mathematically Correct and Biologically Relevant?” Functional Ecology 18: 283–289.

[ele70399-bib-0036] Kozłowski, J. , M. Konarzewski , and M. Czarnoleski . 2020. “Coevolution of Body Size and Metabolic Rate in Vertebrates. A Life‐History Perspective.” Biological Reviews 95: 1393–1417. 32524739 10.1111/brv.12615 PMC7540708

[ele70399-bib-0037] Kozłowski, J. , M. Konarzewski , and A. T. Gawelczyk . 2003. “Cell Size as a Link Between Noncoding DNA and Metabolic Rate Scaling.” Proceedings of the National Academy of Sciences of the United States of America 100: 14080–14085. 14615584 10.1073/pnas.2334605100 PMC283549

[ele70399-bib-0038] Kozłowski, J. , and J. Weiner . 1997. “Interspecific Allometries Are By‐Products of Body Size Optimization.” American Naturalist 149: 352–380.

[ele70399-bib-0039] Maciak, S. , E. Bonda‐Ostaszewska , M. Czarnoleski , M. Konarzewski , and J. Kozłowski . 2014. “Mice Divergently Selected for High and Low Basal Metabolic Rates Evolved Different Cell Size and Organ Mass.” Journal of Evolutionary Biology 27: 478–487. 24417348 10.1111/jeb.12306

[ele70399-bib-0040] Malerba, M. E. , and D. J. Marshall . 2021. “Larger Cells Have Relatively Smaller Nuclei Across the Tree of Life.” Evolution Letters 5: 306–314. 34367657 10.1002/evl3.243 PMC8327945

[ele70399-bib-0041] May, M. L. 1979. “Energy Metabolism of Dragonflies (Odonata: Anisoptera) at Rest and During Endothermic Warm‐Up.” Journal of Experimental Biology 83: 79–94.

[ele70399-bib-0042] Moore, M. P. , J. Shaich , and J. T. Stroud . 2023. “Upslope Migration Is Slower in Insects That Depend on Metabolically Demanding Flight.” Nature Climate Change 13: 1063–1066.

[ele70399-bib-0043] Organ, C. L. , A. M. Shedlock , A. Meade , M. D. Pagel , and S. V. Edwards . 2007. “Origin of Avian Genome Size and Structure in Non‐Avian Dinosaurs.” Nature 446: 180–184. 17344851 10.1038/nature05621

[ele70399-bib-0044] Payne, J. L. , N. A. Heim , M. L. Knope , and C. R. McClain . 2014. “Metabolic Dominance of Bivalves Predates Brachiopod Diversity Decline by More Than 150 Million Years.” Proceedings. Biological Sciences 281: 20133122. 24671970 10.1098/rspb.2013.3122 PMC3996599

[ele70399-bib-0045] Pélabon, C. , C. Firmat , G. H. Bolstad , et al. 2014. “Evolution of Morphological Allometry.” Annals of the New York Academy of Sciences 1320: 58–75. 24913643 10.1111/nyas.12470

[ele70399-bib-0046] Peters, R. H. 1986. The Ecological Implications of Body Size. Cambridge University Press.

[ele70399-bib-0047] Pettersen, A. K. , M. D. Hall , C. R. White , and D. J. Marshall . 2020. “Metabolic Rate, Context‐Dependent Selection, and the Competition‐Colonization Trade‐Off.” Evolution Letters 4: 333–344. 32774882 10.1002/evl3.174 PMC7403701

[ele70399-bib-0048] Pettersen, A. K. , C. R. White , and D. J. Marshall . 2016. “Metabolic Rate Covaries With Fitness and the Pace of the Life History in the Field.” Proceedings. Biological Sciences 283: 20160323. 27226476 10.1098/rspb.2016.0323 PMC4892794

[ele70399-bib-0049] R Core Team . 2021. R: A Language and Environment for Statistical Computing. R Foundation for Statistical Computing. https://www.R‐project.org/.

[ele70399-bib-0050] Reinhold, K. 1999. “Energetically Costly Behaviour and the Evolution of Resting Metabolic Rate in Insects.” Functional Ecology 13: 217–224.

[ele70399-bib-0051] Revell, L. J. 2024. “Phytools 2.0: An Updated R Ecosystem for Phylogenetic Comparative Methods (and Other Things).” PeerJ 12: e16505. 38192598 10.7717/peerj.16505 PMC10773453

[ele70399-bib-0052] Revell, L. J. , and L. J. Harmon . 2022. Phylogenetic Comparative Methods in R. Princeton University Press.

[ele70399-bib-0053] Riveros, A. J. , and B. J. Enquist . 2011. “Metabolic Scaling in Insects Supports the Predictions of the WBE Model.” Journal of Insect Physiology 57: 688–693. 21296084 10.1016/j.jinsphys.2011.01.011

[ele70399-bib-0054] Rombaut, L. M. K. , E. J. R. Capp , C. R. Cooney , E. C. Hughes , Z. K. Varley , and G. H. Thomas . 2022. “Allometric Conservatism in the Evolution of Bird Beaks.” Evolution Letters 6: 83–91. 35127139 10.1002/evl3.267 PMC8802239

[ele70399-bib-0055] Rubner, M. 1883. “Ueber den Einfluss der Körpergröße auf Stoff und Kraftwechsel.” Zeitschrift für Biologie 19: 535–562.

[ele70399-bib-0056] Schramm, B. W. , A. M. Labecka , A. Gudowska , et al. 2021. “Concerted Evolution of Body Mass, Cell Size and Metabolic Rate Among Carabid Beetles.” Journal of Insect Physiology 132: 104272. 34186071 10.1016/j.jinsphys.2021.104272

[ele70399-bib-0057] Smaers, J. B. , and F. J. Rohlf . 2016. “Testing Species' Deviation From Allometric Predictions Using the Phylogenetic Regression.” Evolution (N Y) 70: 1145–1149. 10.1111/evo.12910 27060983

[ele70399-bib-0058] Starostová, Z. , L. Kratochvíl , and D. Frynta . 2005. “Dwarf and Giant Geckos From the Cellular Perspective: The Bigger the Animal, the Bigger Its Erythrocytes?” Functional Ecology 19: 744–749.

[ele70399-bib-0059] Starostová, Z. , L. Kubická , M. Konarzewski , J. Kozłowski , and L. Kratochvíl . 2009. “Cell Size but Not Genome Size Affects Scaling of Metabolic Rate in Eyelid Geckos.” American Naturalist 174: E100–E105. 10.1086/603610 19604072

[ele70399-bib-0060] Strotz, L. C. , E. E. Saupe , J. Kimmig , and B. S. Lieberman . 2018. “Metabolic Rates, Climate and Macroevolution: A Case Study Using Neogene Molluscs.” Proceedings of the Royal Society B: Biological Sciences 285: 20181292. 10.1098/rspb.2018.1292 PMC6125889 30135165

[ele70399-bib-0061] Suárez‐Tovar, C. M. , M. Rocha‐Ortega , A. González‐Voyer , D. González‐Tokman , and A. Córdoba‐Aguilar . 2019. “The Larger the Damselfly, the More Likely To Be Threatened: A Sexual Selection Approach.” Journal of Insect Conservation 23: 535–545.

[ele70399-bib-0062] Svensson, E. I. , S. J. Arnold , R. Bürger , et al. 2021. “Correlational Selection in the Age of Genomics.” Nature Ecology & Evolution 5: 562–573. 33859374 10.1038/s41559-021-01413-3

[ele70399-bib-0063] Svensson, E. I. , M. Gómez‐Llano , and J. T. Waller . 2023. “Out of the Tropics: Macroevolutionary Size Trends in an Old Insect Order Are Shaped by Temperature and Predators.” Journal of Biogeography 50: 489–502.

[ele70399-bib-0064] Tsuboi, M. , W. van der Bijl , B. T. Kopperud , et al. 2018. “Breakdown of Brain–Body Allometry and the Encephalization of Birds and Mammals.” Nature Ecology & Evolution 2: 1492–1500. 30104752 10.1038/s41559-018-0632-1

[ele70399-bib-0066] Uyeda, J. C. , J. Eastman , and L. J. Harmon . 2020. “bayou: Bayesian Fitting of Ornstein‐Uhlenbeck Models to Phylogenies R Package.”

[ele70399-bib-0065] Uyeda, J. C. , and L. J. Harmon . 2014. “A Novel Bayesian Method for Inferring and Interpreting the Dynamics of Adaptive Landscapes From Phylogenetic Comparative Data.” Systematic Biology 63: 902–918. 25077513 10.1093/sysbio/syu057

[ele70399-bib-0067] Uyeda, J. C. , M. W. Pennell , E. T. Miller , R. Maia , and C. R. McClain . 2017. “The Evolution of Energetic Scaling Across the Vertebrate Tree of Life.” American Naturalist 190: 185–199. 10.1086/692326 28731792

[ele70399-bib-0068] van der Bijl, W. 2018. “Phylopath: Easy Phylogenetic Path Analysis in R.” PeerJ 2018: e4718. 10.7717/peerj.4718 PMC5923215 29713568

[ele70399-bib-0069] Voje, K. L. , and T. F. Hansen . 2013. “Evolution of Static Allometries: Adaptive Change in Allometric Slopes of Eye Span in Stalk‐Eyed Flies.” Evolution (N Y) 67: 453–467. 10.1111/j.1558-5646.2012.01777.x 23356617

[ele70399-bib-0070] Voje, K. L. , T. F. Hansen , C. K. Egset , G. H. Bolstad , and C. Pélabon . 2014. “Allometric Constraints and the Evolution of Allometry.” Evolution (N Y) 68: 866–885. 10.1111/evo.12312 24219593

[ele70399-bib-0071] von Hardenberg, A. , and A. Gonzalez‐Voyer . 2012. “Disentangling Evolutionary Cause‐Effect Relationships With Phylogenetic Confirmatory Path Analysis.” Evolution (N Y) 67: 378–387. 10.1111/j.1558-5646.2012.01790.x 23356611

[ele70399-bib-0072] Waller, J. T. , and E. I. Svensson . 2017. “Body Size Evolution in an Old Insect Order: No Evidence for Cope's Rule in Spite of Fitness Benefits of Large Size.” Evolution (N Y) 71: 2178–2193. 10.1111/evo.13302 28685868

[ele70399-bib-0073] West, G. B. , J. H. Brown , and B. J. Enquist . 1997. “A General Model for the Origin of Allometric Scaling Laws in Biology.” Science 1979, no. 276: 122–126. 10.1126/science.276.5309.122 9082983

[ele70399-bib-0074] White, C. R. , L. A. Alton , C. L. Bywater , E. J. Lombardi , and D. J. Marshall . 2022. “Metabolic Scaling Is the Product of Life‐History Optimization.” Science 1979, no. 839: 834–839. 10.1126/science.abm7649 35981018

[ele70399-bib-0075] White, C. R. , T. M. Blackburn , and R. S. Seymour . 2009. “Phylogenetically Informed Analysis of the Allometry of Mammalian Basal Metabolic Rate Supports Neither Geometric nor Quarter‐Power Scaling.” Evolution (N Y) 63: 2658–2667. 10.1111/j.1558-5646.2009.00747.x 19519636

[ele70399-bib-0076] White, C. R. , D. J. Marshall , L. A. Alton , et al. 2019. “The Origin and Maintenance of Metabolic Allometry in Animals.” Nature Ecology & Evolution 3: 598–603. 30886370 10.1038/s41559-019-0839-9

[ele70399-bib-0077] White, C. R. , and R. S. Seymour . 2005. “Allometric Scaling of Mammalian Metabolism.” Journal of Experimental Biology 208: 1611–1619. 15855392 10.1242/jeb.01501

[ele70399-bib-0078] Wilson, E. O. 1999. The Diversity of Life. Belknap Press of Harvard University Press.

