# Supplementary material for: Macroevolution of Steep Interspecific Metabolic Allometry in an Old Insect Order
Source: Ecol Lett. 2026 May 17;29:e70399. doi: 10.1111/ele.70399 (PMC13180310; doi:10.1111/ele.70399)
Supplement: Supplementary file 1 — Figure S1: Marginal ancestral state reconstruction and transition rates (Revell and Harmon 2022) for the flight behaviour of 67 Odonata species from nine families over 237 million years across the time‐calibrated phylogeny. Transition rates (shown in left bottom corner) from flier to percher were much higher than vice versa, with percher being the ancestral state. Reconstruction was performed by model weighting based on four different transition matrices and stochastic character mapping using the fitMk function and 701 simulations using the simmap function in the phytools R package (Revell 2024). Figure S2: Macroevolution of standard metabolic rate (SMR) for fliers (tan circles) and perchers (purple circles) over 237 million years across the time‐calibrated Odonata phylogeny (63 species of nine families). SMR strongly varied across the phylogeny and evolved gradually and in concert with body mass and flight behaviour. The colour gradient shows the observed trait values at the tips and estimated values along the internal phylogeny. The histogram depicts the distribution of trait values at the tips. The tree was visualized using the contMap function in the phytools R package (Revell 2024), which creates maps based on a Brownian Motion model. Figure S3: Macroevolution of mass‐independent standard metabolic rate (SMR) for fliers (tan tip circles) and perchers (purple tip circles) over 237 million years across the time‐calibrated Odonata phylogeny (63 species) based on a reversible‐jumb Markov chain Monte Carlo analysis of the multi‐regime Ornstein‐Uhlenbeck model (Uyeda and Harmon 2014). Evidence for regime shifts (circles on phylogeny) in mass‐independent SMR was limited since all detected shifts had low posterior probabilities (pp). The colours of the tree edges show the mean value of the trait optimum (θ) for that edge from the posterior sample, and the histogram depicts the frequency of θ across the tree edges. Only shifts with pp ≥ 0.2 are shown. We estimated mass‐ [file ELE-29-0-s001.docx]

Supplementary Materials for

**Macroevolution of steep metabolic allometry in an old insect order**

Daniel Schönberger^1,3^, Moa Metz^2,3^, Masahito Tsuboi^3^, Giuseppe Bianco^3^, Zachary C. DeVries^1^, Andreas Nord^3^ & Erik I. Svensson^3*^

1. Department of Entomology, University of Kentucky, Lexington, KY 40508, USA

2. Department of Biology, [Norwegian University of Science and Technology, NO–7491 Trondheim, NORWAY](https://no.linkedin.com/company/ntnu?trk=public_profile_topcard-current-company)

3. Department of Biology, Lund University, SE–223 62 Lund, SWEDEN.

*Author for correspondence

**Email:** [erik.svensson@biol.lu.se](mailto:erik.svensson@biol.lu.se)

**This PDF file includes:**

Supplementary Methods
Supplementary Figures S1–S12
Supplementary Tables S1–S15
Supplementary References

**Supplementary Methods**

## *Descriptive statistics of SMR, body traits, and cellular traits*

The individual body mass (dry mass) covered a 274-fold variation (mean ± standard error [SE]: 85.8 ± 4.0 mg, range: 2.0–548.4 mg, n = 647) whereas the species means of body mass covered a roughly hundred-fold variation (mean: 104.5 ± 12.5 mg, range: 3.6–361.9 mg, n = 64; Supplementary Table S10, Supplementary Fig. S10). The individual standard metabolic rate (SMR) covered a roughly thousand-fold variation (mean: 39.6 ± 2.1 ml CO^2^ × 10^–4^/min, range: 0.5–518.7 ml CO^2^ × 10^–4^/min, n = 646) whereas the species means of SMR covered a 74-fold variation (mean: 45.3 ± 7.6 ml CO^2^ × 10^–4^/min, range: 2.3–170.6 ml CO^2^ × 10^–4^/min, n = 63; Supplementary Table S10, Supplementary Fig. S10). For body volume and body surface area, an individual variation of a factor of 62 (mean: 465.2 ± 51.9 mm^3^, range: 30.1–1870.1 mm^3^, n = 87) and thirteen was found (mean: 50.1 ± 4.0 mm^2^, range: 10.9–145.7 mm^2^, n = 87), respectively. The species means of the same traits covered a 49‑fold (mean: 496.1 ± 73.0 mm^3^, range: 33.3–1634.4 mm^3^, n = 47) and eleven‑fold variation (mean: 52.9 ± 5.8 mm^2^, range: 11.9–131.6 mm^2^, n = 47), respectively (Supplementary Table S14, Supplementary Fig. S10). A two‑fold variation was found in species means for both nucleus and cell size (n = 20, Supplementary Table S13; nucleus size mean: 43.7 ± 1.7 µm^2^, range: 29.7–57.8 µm^2^; cell size mean: 135.3 ± 5.8 µm^2^, range: 88.0–188.9 µm^2^).

## *Fieldwork and specimen collection*

We captured adult dragonflies and damselflies from different species at nineteen field sites in the regions of Skåne and Blekinge in southern Sweden between May and August 2021 and 2022 (Supplementary Figure S5, Supplementary Table S9) as part of an ongoing long-term field study focused on Odonata (Le Rouzic *et al.* 2015; Svensson *et al.* 2020). This regular sampling was complemented by targeted catching of rare species at fifteen localities in the regions of Skåne and Blekinge (Supplementary Fig. S5, Supplementary Table S9). All collected specimens (n = 562) were identified using the field guide of Dijkstra *et al.* (2020) and kept in 14 x 24 cm mesh cages until further processing. Mating individuals were kept together and their copulation status was recorded. Only mature specimens were used to obtain metabolic data, but we also used immature individuals for hemolymph extraction. In addition, we collected 89 dragonflies and damselflies from ten species at three field sites in Lexington and Cave Run Lake (Kentucky, USA) in October and September 2023. These specimens were identified in the lab using field guides (Abbott 2015; Paulson 2011).

## *Measurements and obtaining of standard metabolic rate*

Field-captured specimens were stored in a cold room at 5 °C and measured the next day. All individuals were acclimated to room temperature for at least one hour before measuring. To estimate the standard metabolic rate (SMR), the rate of carbon dioxide production (VCO_2_) was measured via stop‑flow respirometry (Lighton 2008) suitable for small animals (Lighton & Halsey 2011). Open-ended polypropylene metabolic chambers were modified in size to fit different Odonata species (sizes in cm: 6.5 x 1.5, 7.5 x 1.5, 8.5 x 1.5, 9.5 x 2.5 or 15 x 3.5; see provided raw data on *Zenodo*) to obtain optimal signal-to-noise ratios for the metabolic response and minimize movement. Each end of the chambers was sealed with a rubber stopper connected to valved polypropylene couplings (Omega Engineering, Manchester, England) connected to bev‑a‑line IV tubing (Thermo Fisher Scientific, New Jersey, USA). Influent air was scrubbed from water vapor using Drierite (granular calcium sulfate; Alfa Aesar, Kandel, Germany) and from carbon dioxide (CO_2_) using ascarite II (Acros Organics, Thermo Fisher Scientific) and was then pushed through the chamber using an SS4 subsampler pump (Sable Systems International, Las Vegas, Nevada, USA) (Lighton & Halsey 2011). The flow rate ranged from 170 to 500 ml per minute (standard temperature and pressure, dry [STPD]), depending on the size of the species (Supplementary Table S10). The water vapor in the effluent air was removed by an anhydrous magnesium perchlorate drying column (Honeywell, Charlotte, North Carolina, USA), and a CA 10a CO_2_ analyzer (Sable Systems International) was used to measure CO_2_ enrichment in the air.

The chambers were first flushed until CO_2_ and H_2_O reached zero. Then, the chambers were sealed and the animal was incubated for 20–60 min depending on the species (Supplementary Table S10) at 25 ± 1 °C in a climate test chamber (C180, Vötsch Industrietechnik, Ballingen, Germany), after which the chamber atmosphere was flushed to measure CO_2_ produced by the animal. *ExpeData* v.1.927 (Sable Systems International 2023) was used to calculate VCO_2_ data using equation 10.4 of Lighton (2008). The total CO_2_ produced by the specimen was calculated by integrating CO_2_ production over the time taken to evacuate the atmosphere. This value was then divided by incubation time to derive VCO_2_ in ml CO_2_/min.

A precision scale (Samo Tronic Precisa 100A–300M, Malmö, Sweden; ± 0.1 mg SE) was used to measure the fresh mass (before and after each metabolic procedure) and dry mass of each specimen (mg). Before measuring the dry mass, the individuals were killed by freezing at –8 °C for 20 min and dried overnight at 60 °C in an oven (Termaks, Kungsbacka, Sweden). Based on a pilot study, this procedure was sufficient to obtain constant mass. Unless stated otherwise, dry mass was used in all analyses.

For the 89 specimens collected in the US, we used the same procedure to estimate the SMR and body mass, with the following methodological deviations due to differences in available equipment. An electric air compressor (8.0 Gal., 1.0 HP Ultra Quiet and Oil‑Free Electric Air Compressor, California Air Tools, San Diego, California, USA) delivered air into a purge‑gas generator (Spectra 30, Parker Balston, Cleveland, Ohio, USA) at 440–630 pascals which removed CO_2_ and water vapor. The air was then moved into a 360‑liter mixing tank to permit equilibration to atmospheric pressure. Air was pulled out of the manifold by a MFS2 mass flow system (Sable Systems International) through a Drierite-Ascarite-Drierite column (Drierite‑W.A. Hammond Drierite Company Ltd., Xenia, Ohio, USA; Ascarite-Thomas Scientific, Swedesboro, New Jersey, USA) to ensure CO_2_‑free and dry air. The air then exited the mass flow system at a rate of 100 ml per minute at STPD and passed through a second Drierite-Ascarite-Drierite column. The air passed through a sample injection port (Trace Gas Sample Kit, LI‑COR Biosciences, Lincoln, Nebraska, USA), followed by a LI‑7000 CO_2_/H_2_O analyzer (LI‑COR Biosciences) to measure CO_2_ enrichment in the air. Respirometry chambers were manufactured out of 3 ml, 10 ml, or 20 ml syringes (Becton, Dickinson and Company, Franklin Lakes, New Jersey, USA), in which the Odonata specimens were placed individually. The air flushed out of the syringes at a rate of 0.7 ml/min for 5–10 minutes (UnicLife Air Pump, Hasty, Colorado, USA). Syringes were sealed, and Odonata individuals were allowed to respire for 5–57 min (mean: 44 min ± 0.89 SE). After this time, a 0.2–4.0 ml air sample was injected into the injection port to measure the enrichment of CO_2_. Carbon dioxide excretion was recorded in ppm, then using flow rate converted into ml CO_2_ consumed per hour. The total area under the curve for each sample injection was integrated against time to determine the total amount of CO_2_ excreted by the organism. This value was further corrected to account for the injection volume, respirometry chamber volume, and time spent in the chamber to determine the VCO2 rate (ml CO_2_/min). The precision scale Explorer EX125D (OHAUS, Parsippany, New Jersey, USA; d0.1 mg SE) was used to measure the fresh and dry mass of each specimen.

Overall, the SMR of 645 individuals of 57 species was measured. Nineteen measurements were excluded due to technical errors during the measurement (sealing issues of the chambers or heavily truncated peaks of the CO_2_ curve).

We supplemented our SMR and body mass dataset with published data from Table 1 of May (May 1979a), which included the means of oxygen consumption rate and fresh mass of 121 individuals of thirteen Nearctic dragonfly species (mean within-species sample size: 9.3 ± 0.8 SE) measured at 25 °C in a temperature room. We only included data measured at 30 °C body temperature (T_b_ in Table 1 of (May 1979a) to reduce confounding effects of body temperature on SMR.

All SMR measurements used in this study were quantified at 25 °C. The final combined dataset used for the interspecific metabolic allometry in our study included 747 individuals of 63 Odonata species belonging to nine families, with a mean sample size per species of 11.1 (standard deviation [SD]: 7.5, range: 1–30; Supplementary Table S10; Supplementary Figure S12).

## *Estimating volume and surface area*

We used three-dimensional imaging via x‑ray micro-computed tomography (µCT) to estimate the body volume (mm^2^) and surface area (mm^3^) of the Odonata specimens, including their thorax and abdomen (the legs, wings, and antennae were excluded). This is a non-destructive approach to obtain high-resolution morphological and anatomical data for quantitative and qualitative analysis of animals, including insects (Keklikoglou *et al.* 2021; Li *et al.* 2008; Mizutani & Suzuki 2012; Schambach *et al.* 2010). It generates a three-dimensional tomographic model of a specimen by combining multiple two-dimensional radiographic image projections into sequential cross-sectional slices (Keklikoglou *et al.* 2021; Li *et al.* 2008; Mizutani & Suzuki 2012; Schambach *et al.* 2010). These are combined in a scale-accurate volumetric model (Keklikoglou *et al.* 2021; Li *et al.* 2008; Mizutani & Suzuki 2012; Schambach *et al.* 2010).

The collected specimens were killed by freezing at –8 °C for 20 min and soaked in acetone for 12–36 hours. The dried specimens were stored in propylene envelopes for subsequent scanning. Three-dimensional images were acquired with an Extra-Ultra-High-Resolution U‑CT^XUHR^ system (MILabs, Houten, Netherlands) and the integrated *MILabs Acquisition* v.11.00 software. The system uses a rotational gantry design (Li *et al.* 2008; Schambach *et al.* 2010), where the X‑ray source and the detector rotate around a fixed bed (diameter: 5.5 cm for dragonflies, 2.5 cm for damselflies). Two specimens were analyzed simultaneously in the same bed. The scanning parameters were set up as follows: isotropic voxel size = 40 µm per pixel, source voltage = 65 kV, source current = 0.13 mA, exposure time = 75 ms, source filter “air”, and image rotation step = 0.25º. Full 360‑degree rotations were conducted, resulting in 1440 projections for each reconstruction. The obtained raw image projections were reconstructed using the integrated *MILabs Reconstruction* v.13.11 software and the resultant *.nii* files were visually reviewed using *PMOD* v.4.005 (PMOD Technologies, Fällanden, Switzerland). The latter program was used to select the desired section of the reconstruction. The µCT procedure was performed at the Lund University Bioimaging Center in Sweden under the supervision of Marie Sydoff.

After reconstruction into a three-dimensional volumetric model, the isosurface of the specimens was extracted in *arivis Vision4D* v.4.0 (Carl Zeiss Microscopy Software Center Rostock, Rostock, Germany) and exported as *.obj* file. In *Blender* v.4.0.1 (Community 2018), the *.obj* file was imported, scaled to the correct size, and exported as *.stl* file. The *.stl* file was re‑imported to *Blender*, where the volume and surface area estimation of the reconstruction was conducted as follows: The remaining noise, the legs, wings, and antennae were manually removed. The resultant reconstruction (Supplementary Fig. 6A) was remeshed using a voxel size of 0.01–0.09, and the mesh was manually adjusted to approximate the specimen reconstruction (Supplementary Fig. 6B). For the estimates of the thorax and abdomen, the head was removed and both were separated (Supplementary Fig. 6C). The volume and surface area of the meshes were calculated using the *Mesh: 3D‑Print Toolbox* add‑on. We tested the repeatability of the reconstruction process by reiterating the procedure for seven randomly chosen species. This test showed that the reconstruction process was highly repeatable, with marginal deviations of the volume and surface area estimates from the original estimates (mean deviations: body volume: 1.67% ± 0.44 SE, body surface area 2.49% ± 0.85 SE, thorax volume: 2.77% ± 1.12 SE, thorax surface area 3.24% ± 0.98 SE, abdomen volume: 5.06% ± 1.23 SE, abdomen surface area 4.00% ± 0.75 SE). The reconstruction process was supervised by Jonas Ahlstedt (Lund University Bioimaging Center).

In total, the body volume and surface area were obtained for 87 individuals of 47 Odonata species, with a mean sample size per species of 1.85 (SD: 0.86, range: 1–5; Supplementary Table S14). For all analyses, the volume and surface area estimates were log_10_-transformed and species-specific means were calculated.

## *Measuring cell and nucleus size*

We measured hemocyte cell properties from the hemolymph of field-collected specimens. We considered these estimates of hemocyte nucleus and cell size as a proxy for the overall body cells since there are likely only marginal differences in relative cell size between different cell types (Schramm *et al.* 2021; Stevenson *et al.* 1995). Collected specimens were stored at 2 °C until further processing. Hemolymph was extracted on the same day or the day after collection. To extract the hemolymph, the tip of the abdomen was cut off and dipped into a 5 ml Eppendorf tube containing 25 µl formaldehyde (37%) in phosphate-buffered saline to fixate the cells.

To stain the fixed cells, a stock solution containing Anthraquinone dye DRAQ5*™* (Thermo Fisher Scientific, Waltham, Massachusetts, USA) was used by diluting it with phosphate-buffered saline to 5 µM. This dye can be excited at wavelengths of 488–647 nm, allowing its detection by a high-resolution imaging system. For this, we used the Operetta CLS™ High Content Analysis System (PerkinElmer, Waltham, Massachusetts, USA) and an imaging plate with 96 wells. For our analyses, 62 wells were each filled with 100 µL of stock solution and 20 µL of the fixed cell suspension of one species. After 30 min of sedimentation, the cells were analyzed with the integrated *Harmony High‑Content Imaging and Analysis Software* v.4.9, allowing the quantification of the cell and nucleus sizes (i.e., area) (Supplementary Fig. S7) with the aid of a customized protocol. This work was carried out at the Lund Stem Cell Center FACS Facility in Sweden under the supervision of Anna Fossum.

For all analyses, the cell and nucleus sizes were log_10_‑transformed and species‑specific means were calculated. In total, the cell and nucleus sizes were estimated for 20 Odonata species, with a mean sample size of 1.45 per species (SD: 0.51, range: 1–2; Supplementary Table S13).

***Classification of flight behavior***

The flight behavior of Odonata species can be categorized into fliers and perchers (Corbet 1962, 1999; Corbet & May 2008). Both flight behaviors are expected to differ in energy demands because they contrast in the time spent flying relative to perching and in thermoregulatory strategies (Corbet 1962, 1999; Corbet & May 2008; May 1979a). We classified the flight behaviors of our species based on the Odonate Phenotypic DataBase (Waller *et al.* 2019) and field observations (Supplementary Table S11).

## *General Statistical analyses*

All statistical analyses were performed with *RStudio* v.2023.09.1+494 (RStudio Team 2022) in *R* v.4.3.1 (R Core Team 2021). The *ggplot2* v.3.4.4 (Wickham 2016), *viridis* v.0.6.4 (Garnier *et al.* 2023) and *gridextra* v.2.3 (Auguie 2017) *R* packages were used for visualization and the *dplyr* v.1.1.4 (Wickham *et al.* 2023), and *tidyverse* v.2.0.0 (Wickham *et al.* 2019) *R* packages for data handling. The *ape* v.5.7‑1 (Paradis & Schliep 2019), *caper* v.1.0.3 (Orme *et al.* 2018), *treeplyr* v.0.1.11 (Harmon 2023), *phytools* v.2.1‑1 (Revell 2024), and *geiger* v.2.0.11 (Pennell *et al.* 2014) *R* packages were used for reading, plotting and manipulating phylogenetic trees and analyzing phylogenetic comparative data (Revell & Harmon 2022). Standard statistical tests such as linear models (Sokal & Rohlf 2009) and complementary phylogenetic comparative methods were used (Garamszegi 2014a; von Hardenberg & Gonzalez-Voyer 2012; Harmon 2019; Revell & Harmon 2022; Uyeda *et al.* 2017; Uyeda & Harmon 2014) and are described in detail below.

For all statistical tests, p ≤ 0.05 was assumed as the significance threshold. To compare among and select the best‑fitting maximum-likelihood models, we used the sample-size corrected Akaike Information Criterion (AICc) (Akaike 1974; Cavanaugh 1997) and Akaike weights ω. The latter represents the weight of evidence supporting each fitted model (Burnham & Anderson 2003). ΔAICc is the AICc value of a model of interest minus the value of the model with the lowest AICc (Burnham & Anderson 2003). Models with ΔAICc ≤ 2 were considered to have statistical support (Burnham & Anderson 2003).

If stated, the standard errors of the means were incorporated into the models, as recommended (Liam Revell, personal communication). Standard errors were available for log_10_ SMR, log_10_ body mass, log_10_ nucleus size, log_10_ cell size, log_10_ body volume, log_10_ body surface area, and mass-independent SMR. As estimates of the standard errors of mass-independent SMR, we used the square root of the sum of squared log_10_ SMR and squared log_10_ body mass. For the Nearctic species extracted from May (1979a) and species with sample sizes lower than three (or lower than two in the case of volume and surface area), standard errors were not available. Hence, the mean standard errors of all other dragon- or damselfly species were used for these traits. For nucleus and cell size, within-individual standard errors based on a large amount of analyzed hemocyte cells per individual were used because within-species variation was not obtainable.

Unless stated otherwise, mean values and estimates are given with standard errors or 95% confidence intervals (CI). For all Bayesian models, the posterior mean and the 95% highest posterior density intervals (HPD) are reported.

## *Analyzing metabolic allometry*

To analyze metabolic allometry patterns, ordinary least squares (OLS) regression has been shown to outperform reduced major-axis regression (Symonds & Elgar 2002; White 2011; White & Seymour 2005). The latter method does not only assume error in the estimation of the dependent variable, as OLS models do, but also in the independent variable (Symonds & Elgar 2002; White 2011; White & Seymour 2005). Therefore, we used OLS to study the relationship between SMR and body mass using a log_10_‑transformation to account for allometric scales:

1. log_10_SMR = log_10_b + a log_10_(body mass) + ε

with *ε* representing the error term of the model, *b* the metabolic scaling coefficient, and *a* the scaling exponent. For the interspecific analyses, we used species-specific mean values. Visual inspection of residual plots revealed no apparent deviations from homoscedasticity or normality. Confidence intervals were calculated to evaluate if the allometric slopes differed significantly from the traditionally expected allometric slopes of 0.67 or 0.75 (Dodds *et al.* 2001; Kleiber 1932, 1961; Rubner 1883; West *et al.* 1997, 1999; White & Seymour 2003).

Fresh and dry mass were highly correlated across 626 individuals of 54 species (r = 0.99; Supplementary Fig. S8), allowing the conversion of the dragonfly fresh mass of May (1979) to dry mass using the mean conversion factor (0.351749) found for our dragonfly dataset (n = 343). A similar conversion factor of 0.34 was found by May (1979a). To transform the oxygen consumption rate data of May (1979) to VCO_2_ (prior to the log_10_-transformation), a respiratory quotient (RQ) of 0.79 was assumed (Duncan & Crewe 1993). We combined both datasets and subsequently evaluated the fit of the OLS regression. Conversions using RQs assumed by other insect studies, namely RQ = 1.0 (May 1979a), RQ = 0.84 (Addo-Bediako *et al.* 2002; Chown *et al.* 2007), and RQ = 0.8 (White *et al.* 2019), resulted in similar or worse fitting regression models (Supplementary Table S15).

Linear regression assumes independent model residuals (Sokal & Rohlf 2009). This assumption is usually violated when using interspecific data because closely related taxa tend to have more similar residuals than distantly related ones due to their shared evolutionary history (Felsenstein 1985; Harmon 2019; Harvey & Pagel 1991; Revell 2010). This leads to increased Type I error rates, inflated degrees of freedom, an overestimation of the strength of the relationship and a drastic increase in the estimation of the scaling exponent (Garland *et al.* 2005; Harvey & Pagel 1991; O’Connor *et al.* 2007). Phylogenetic-informed approaches are therefore preferred to analyze metabolic allometries (White 2011), as shown in mammals (Capellini *et al.* 2010) and insects (Chown *et al.* 2007).

To account for phylogenetic relatedness, we performed phylogenetic generalized least squares (PGLS) models (Garland & Ives 2000; Grafen 1989; Harmon 2019; Martins & Hansen 1997; Pagel 1997) using the *phylolm* v.2.6.2 *R* package (Ho & Ane 2014). We extracted the most comprehensive time-calibrated Odonata phylogeny available (Waller & Svensson 2017) and pruned it to match the species in our study (Figs. 3–4, Supplementary Figs. S1–S3). Seven of our 63 taxa were not available in the phylogeny and their tip labels were hence reassigned to the most closely related proxy species of the same genus (Supplementary Table S11). Our main results were not affected by the choice of these replacement species (Supplementary Table S12).

For the PGLS, a transformation of the phylogenetic tree into a variance‑covariance matrix is performed, where the diagonal values of the matrix represent the path length from the root to the phylogeny tip and the off‑diagonal values represent the shared evolutionary history of a given pair of taxa given as the time of shared evolution from the tree root to the last common ancestor (Freckleton *et al.* 2002; Pagel 1999). PGLS addresses the interspecific autocorrelation caused by phylogeny by implementing the variance-covariance matrix into the model, resulting in modified intercept and slope estimates (Freckleton *et al.* 2002; Pagel 1999).

To improve the fit of the phylogeny to the data and estimate the phylogenetic signal of the residuals (Revell 2010), branch length transformations were performed by estimating the parameters λ, δ, and κ using maximum likelihood (Pagel 1997, 1999; Revell & Harmon 2022). For the specific tree transformations, the internal branches are multiplied by λ, the node heights are raised to the power of δ, or each branch length is raised to the power of κ, respectively (Pagel 1999). The parameter λ varies between 0 and 1, with λ = 0 representing phylogenetic independence equivalent to OLS. λ = 1 signifies a pattern of variation at the trips predicted by the phylogeny under a Brownian Motion (BM) model where the similarity between species covaries directly with their shared evolutionary time. Values of λ between 0 and 1 indicate that the trait evolution is evolutionary correlated but less than expected under BM. When λ is significantly higher than zero, a phylogenetic perspective of the statistical model is therefore necessary (Freckleton *et al.* 2002; Pagel 1997, 1999). For δ < 1, the length of external nodes is reduced, indicating a decrease in evolutionary rate over time (Pagel 1999). δ > 1 represents the opposite model with an increase in length and evolutionary rate, and δ = 1 models a BM process where the tree remains unchanged (Pagel 1999). For κ < 0, all branch lengths are equal, indicating a punctuated evolutionary model (Pagel 1997, 1999). On the other hand, κ = 1 depicts a BM scenario where the tree is unchanged, and κ > 1 increases the length of long branches disproportionally to short branches suggesting that trait variance accumulates faster than total divergence (Pagel 1997, 1999).

We also fitted the PGLS models to Early Burst (Harmon *et al.* 2010) and two Ornstein-Uhlenbeck (OU) models with the ancestral state estimated at the root (OU_fR_) or the ancestral state at the root having a stationary distribution (OU_rR_) (OU models are further discussed in the paragraphs below). This enabled us to fit the PGLS in an evolutionary framework deviating from a BM process (Hansen *et al.* 2008). The Early Burst model hypothesizes that the rate of evolution σ^2^ starts with some initial value at the root of the tree but then declines monotonically through time according to an exponential decay function with the decay parameter *b* (Blomberg *et al.* 2003; Harmon 2019; Harmon *et al.* 2010). To ensure the robustness of the PGLS results, we repeated all PGLS models but using fresh instead of dry mass or excluding species with within-species sample sizes lower than ten (since their mean trait values are likely less reliable, as discussed below). No residual outliers were detected in any PGLS. Estimates and their confidence intervals were determined by 100,000 bootstrap replicates. Measurement errors were set to be estimated (except for PGLS_λ_ models, for which they cannot estimate λ and the error simultaneously).

We performed two-way phylogenetic analysis of covariance (pANCOVA) models (Garamszegi 2014a; Revell & Harmon 2022) including lambda estimation in *phylolm* (Ho & Ane 2014) to test for a difference in metabolic allometric slopes and intercepts between suborders (dragonflies and damselflies) and flight behaviors (fliers and perchers). We specified suborder or flight behavior as the covariate and included the interaction terms (body mass × suborder or flight behavior).

To test for a curvilinear relationship in the metabolic allometry, we compared a linear PGLS_λ_ with the fit of a non‑linear quadratic PGLS_λ_ (log_10_‑scale) (Kolokotrones *et al.* 2010):

1. log_10_SMR = log_10_b + a log_10_(body mass) + c log_10_(body mass)^2^ + ε

with *c* representing the constant of the quadratic term of the model and all other parameters similar to equation 1. A curvilinear relationship would be indicated if the non‑linear PGLS_λ_ shows a better fit than the linear PGLS_λ_ and by a significant quadratic coefficient of the non‑linear PGLS_λ_.

To explore if any of the Odonata families represents a significant outlier in the metabolic scaling relationship, we conducted pANCOVA models (Smaers & Rohlf 2016) using the *gls.ancova* function in the *evomap* v*.*0.0.0.9000 *R* package (Smaers & Mongle 2018). This approach is based on standard PGLS procedures with BM. Using an F‑ratio test, it explores if a complex model that includes a separate intercept and slope for a specific family fits the data better than a PGLS that assumes equal slopes and intercepts for all families (Smaers & Rohlf 2016). This analysis was repeated for all Odonata families with at least one species.

Since the two families Coenagrionidae and Lestidae were detected as outliers in the metabolic scaling relationship based on the pANCOVA approach (see Results), we also fitted PGLS models to estimate the metabolic allometry that excluded both families. This was also done for PGLS models that incorporated within-species sample size or variation (described below).

## *Cross‐validation of SMR predictions*

To assess the predictive accuracy of our phylogenetically informed model, we fitted a PGLS_λ_ model predicting log₁₀(SMR) from log₁₀(body mass). We performed leave‐one‐out cross‐validation (Geisser 1975; Stone 1974), in which each species was excluded in turn, the PGLS model was re‐fit to the remaining species (re‐estimating λ at each step), and the SMR of the omitted species was predicted from its body mass. Predictive accuracy was evaluated by the squared correlation (R²) between observed and predicted SMR values and by the root mean squared error (RMSE) in log₁₀ units. The model showed very high predictive performance (R² = 0.95, RMSE = 0.14), indicating that tip values can be closely approximated from the body‐mass scaling relationship when phylogeny is accounted for. This supports the reliability of the ancestral state reconstructions for SMR within the observed body‐mass range.

## *Incorporating within-species variation*

Most studies use trait means of species to estimate metabolic allometry while assuming no estimation errors as well as ignoring differences in within-species sample size and intraspecific variance in SMR and body mass (Garamszegi 2014b; Garamszegi & Møller 2010). However, substantial within-species variation is expected due to measurement errors and biological variability, including differences between individuals, sexes, and life stages as well as spatial or temporal variability across populations (Garamszegi 2014b). This variation below the species level can lead to considerable deviations from the actual species-specific means and a higher probability of type II statistical errors (Chesher 1991; Manisha 2001). Ignoring heterogeneity in within-species sample size can lead to different model results (Garamszegi & Møller 2010). In the framework of phylogenetic comparative methods, the rate of type I statistical errors increases and the accuracy of parameter estimates decreases with increasing within-species variation and decreasing sampling size, highlighting the need to account for both in the analysis (Felsenstein 2008; Harmon & Losos 2005; Ives *et al.* 2007). When within-species variation occurs, the slope estimates of PGLS models will be underestimated and the estimation of the evolutionary rate σ^2^ and phylogenetic signal K will be affected (Ives *et al.* 2007).

To quantify the within-species variation and dispersion from the species-specific means of log_10_ SMR and log_10_ body mass, we calculated the coefficient of variation (CV) (Garamszegi 2014b). The CV represents the extent of variability relative to the mean for each species and is comparable across different traits and trait value ranges (Garamszegi 2014b; Sokal & Rohlf 2009). A CV lower than one demonstrates low variability, whereas a CV higher than one indicates high trait variance (Garamszegi 2014b). Across all Odonata species, the coefficients of variation (CV) revealed low variation in SMR (mean CV_SMR_: –0.071 ± 0.004 SE, range: –0.171 to –0.008) and body mass (mean CV_BM_ = 0.068 ± 0.008 SE, range: 0.003 to 0.258).

Moreover, we calculated the repeatability for both traits to estimate the proportion of within-species variances to the total variances (the sum of within‑ and between-species variances) (Garamszegi 2014b). The repeatability is close to one if most of the variance resides at the between-species level but around 0.5 if between‑ and within-species variations are similar (Garamszegi 2014b; Nakagawa & Schielzeth 2010). In other words, if the repeatability of a trait is close to one, a single specimen likely represents the true species-specific value, but if the repeatability is low, appropriate statistical treatment of within-species variances is needed (Garamszegi 2014b; Nakagawa & Schielzeth 2010). The repeatability was calculated based on a linear mixed effects model approach using the *lmer* function in the *lme4* v.1.1‑34 *R* package (Bates *et al.* 2015), with confidence intervals estimated by 100,000 parametric bootstrap samples (Garamszegi 2014b; Nakagawa & Schielzeth 2010).

To adjust for differences in sampling size across species (Garamszegi 2014b), we also fitted weighted PGLS_λ_ using the *corPagel* function in *ape* and the *gls* function and *weights* argument in the *nlme* v.3.1‑164 *R* package (Pinheiro *et al.* 2022). In this method, species with larger sampling sizes are assumed to provide more accurate species-specific trait value means and are thus given higher weight in the model (Garamszegi 2014b; Garamszegi & Møller 2010). To avoid too much weight on species with sufficiently large sample sizes, sample sizes were square-root transformed (Garamszegi 2014b; Garamszegi & Møller 2010).

Next, we performed an approach by Ives et al. (Ives *et al.* 2007) to address within-species variances and measurement errors in a phylogenetic framework using the *pgls.Ives* function in *phytools*. This method has been shown to perform best compared to other models that incorporate within-species variation (Garamszegi 2014b). In the PGLS_Ives_ model, a vector of within-species variances in addition to the vector of variances caused by phylogeny is incorporated in the error term (Garamszegi 2014b; Ives *et al.* 2007). The model parameters are estimated by model-fitting iteration processes based on estimated generalized least squares, maximum-likelihood, and restricted-maximum-likelihoods (Garamszegi 2014b; Ives *et al.* 2007).

For this approach, data was available from 627 individuals of 53 species. We used adjusted within-species variances, which incorporate the coefficient of variation for each species combined with the pooled variance over all species (Garamszegi 2014b). Since the model often failed to converge and to ensure the detection of the most likely model, we ran one million model-fitting iterations. Log-likelihood values varied substantially among the converged models (range: –206.8–21.7). Therefore, we considered the model with the highest log-likelihood and all models with a log-likelihood difference of two or less as supported. We extracted the mean slope and mean intercept from these supported models and used the standard deviation of these estimates as uncertainty measures.

## *Comparing suborders and flight behaviors*

To test if dragonflies and damselflies (suborders) or fliers and perchers (flight behaviors) differ in body traits, we fitted phylogenetic analysis of variance (pANOVA) models with lambda estimation (Revell & Harmon 2022) in *phylolm* (Ho & Ane 2014). We specified suborder or flight behavior as discrete predictors and body mass, SMR, nucleus size, cell size, body volume, body surface area, thorax volume, thorax surface area, abdomen volume, or abdomen surface area as continuous response variables (all on log_10_‑scale). For all body traits except body mass, we included body mass as a covariate to account for size. In particular, we were interested in testing if fliers show higher SMR and lower nucleus sizes than perchers. Besides, we expected thorax volume to be strongly correlated with the size of the wing muscles (located in the thorax), which we assumed to be larger in more active species. Therefore, we also tested if fliers have higher thorax volume than perchers, which would be consistent with their higher flying activity.

There was no evidence for a statistical difference between fliers and perchers in body mass (t = –1.42, p = 0.16), SMR (t = –1.53, p = 0.13), body volume (t = –0.43, p = 0.67), thorax volume (t = 1.22, p = 0.23) and thorax surface area (t = 0.48, p = 0.63) (Supplementary Fig. S9). However, perchers had smaller body surface area (t = –2.19, p = 0.034), abdomen volume (t = –2.62, p = 0.012) and abdomen surface area (t = –2.60, p = 0.013) than fliers (Supplementary Fig. S9).

Between suborders, there was no statistical support for a difference in SMR (t = –0.97, p = 0.34), body mass (t = –1.37, p = 0.18) and body surface area (t = 0.51, p = 0.61) (Supplementary Fig. 9). However, damselflies had smaller body volume (t = –2.58, p = 0.014), thorax volume (t = –3.55, p < 0.001) and thorax surface area (t = –4.69, p < 10^–4^) but larger abdomen volume (t = 2.42, p = 0.020) and abdomen surface area (t = 5.28, p < 10^–5^) than dragonflies (Supplementary Fig. S9).

## *Modeling continuous trait evolution*

Since SMR was strongly correlated with body mass (phylogenetic correlation coefficient r = 0.89; Fig. 1), we estimated mass-independent SMR by using the residuals obtained from phylogenetic size-correction via PGLS regression including lambda estimation of log_10_ SMR on log_10_ body mass (Revell 2009), as implemented in the *phyl.resid* function in *phytools*. Using residuals as size-correction is preferred over the SMR body mass ratio due to the mathematical issues related to ratios (Jasienski & Bazzaz 1999; Kratochvíl & Rovatsos 2021; Sokal & Rohlf 2009). Mass-independent SMR thus allowed us to compare the energy expenditure among species while controlling for body mass and separating the evolution of SMR from the evolutionary trends in body mass.

We calculated the phylogenetic signals λ (Pagel 1999) for absolute and mass-independent SMR using the *phylosig* function in *phytools* (Revell & Harmon 2022). The phylogenetic signal describes the tendency for closely related species to resemble one another more than expected by chance based on a BM process of evolutionary change (Blomberg *et al.* 2003; Harmon 2019; Revell *et al.* 2008). Traits with high phylogenetic signals have likely evolved gradually over time, whereas traits lacking a signal are probably either extremely stable or the trait values change rapidly along the phylogeny (Revell *et al.* 2008). A likelihood-ratio test was used to evaluate if λ was higher than zero (Revell & Harmon 2022). Standard errors of the means were incorporated as measurement errors.

To detect the presence of any evolutionary constraints on the interspecific trait variance accumulation, we fitted a single-optimum OU model (Butler & King 2004; Hansen 1997) to absolute and mass-independent SMR. A high α parameter indicates a constraint on minimum or maximum trait values and a trend for all species to return to a specific long-term optimum θ, which suggests the presence of a pullback force operating on a lineage and a tendency to quickly adapt to new conditions (Grabowski *et al.* 2023; Hansen 1997; Hansen *et al.* 2008; Harmon 2019; O’Meara & Beaulieu 2014). Since α scales with tree height, it is recommended to use the phylogenetic half-life t_1/2_ = ln(2)/α instead and interpret it relative to the tree height (Cooper *et al.* 2016; Grabowski *et al.* 2023). The phylogenetic half-life is a measure of the time that is needed before adaptation to a novel regime is expected to be more influential than constraints based on the ancestral state (Hansen 1997). A substantial evolutionary pullback force is likely present when the half‑life is much smaller than the tree length (here: 237 million years [MY]), suggesting that the phylogenetic signal decreases rapidly and there is a minor influence of the past on trait values (Cooper *et al.* 2016; Grabowski *et al.* 2023; Hansen 1997). By contrast, phylogenetic half‑lives substantially higher than the tree height represent evolutionary processes increasingly resembling BM (Cooper *et al.* 2016; Grabowski *et al.* 2023; Hansen 1997). When the phylogenetic half-life is large compared to the tree height and there is evidence for optima outside of realistic trait values, the evolutionary evolution of the trait can be described by a Trend model (Grabowski *et al.* 2023; Hansen 1997). This Trend model approximates a BM process, but clade-specific evolutionary trends are evident so that some clades evolve towards specific metabolic optima with very low rates of adaptation (high phylogenetic half-lives) (Grabowski *et al.* 2023; Hansen 1997).

We expected separate adaptive regimes for each suborder (dragonflies and damselflies) and flight behavior (fliers and perchers). To test this, we searched for different evolutionary optima θ on the phylogeny by fitting multi-optima OU models for each trait with both regime scenarios (Beaulieu *et al.* 2012; Butler & King 2004; Hansen 1997; Revell & Harmon 2022). The OU models were then compared to a BM model where traits evolve as a random walk from the ancestral state with a linear increase in interspecific trait values according to the evolutionary rate parameter σ^2^ and lacking evolutionary constraints on the upper and lower bounds of the trait values (Felsenstein 1973, 1985; Hansen 1997; Harmon 2019; Martins & Hansen 1997). In addition, we compared the fit of these models with multi-rate BM models allowing each regime to evolve at a different evolutionary rate (σ^2^) (O’Meara *et al.* 2006; Revell *et al.* 2018). All models were fitted in the *OUwie* v.2.10 *R* package (Beaulieu & O’Meara 2022), with *root.station* set to *FALSE* to fit the noncensored model of O’Meara et al. (2006) so that all tree edges are assigned to a regime and only one value for the tree root is calculated. The standard errors of the trait means were incorporated as measurement errors. This is recommended to reduce model selection bias towards an OU model due to ignored within-species variation (Silvestro et al. 2015;Liam Revell, personal communication).

To fit the multi-optima OU and multi-rate BM models, the respective regimes were first modeled on the phylogeny by stochastic character mapping based on 701 simulations with random sampling of discrete character histories of the trait under the evolutionary model (Bollback 2006; Huelsenbeck *et al.* 2003; Nielsen 2002) and the Fitzjohn et al. (2009) root prior, as recommended (Revell & Harmon 2022; Liam Revell, personal communication). For the suborder regimes, the *make.simmap* function in *phytools* and an equal‑rate model were used (Revell & Harmon 2022). For the flight behavior regimes, we used the *fitMk* function in *phytools* to fit four different models with different transition rate matrices (an equal‑rate, an all‑rate different, and two customized matrices where the transition in only one direction was allowed). Their fitting was compared using the *anova* function and *simmaps* were created by averaging all models by their model weights so that the *simmap* includes the stochastic character histories with probabilities proportional to each model weight (Revell & Harmon 2022). The marginal posterior probabilities of all combined trees were obtained using the *summary* function of the *simmap* object (Revell & Harmon 2022). The “one‑direction model” best explained the transition between behavioral states (model weight: 0.57), with the transition from perchers to fliers being drastically higher than vice versa (Supplementary Fig. S1). For each multi‑rate BM or multi‑optima OU model, the model was fitted with each of the 701 simulated *simmaps*. The model with the lowest AICc value was selected for the subsequent analysis. This approach ensured the reproducibility of the results by diminishing the stochasticity of the simulation process since slight deviating results were found when running only single simulations. Since fliers were only present in the Anisoptera (dragonfly) suborder, we also performed these maximum-likelihood models but only using the Anisoptera clade.

To test the robustness of the results of the maximum-likelihood and hypothesis-driven evolutionary models and further decipher the evolutionary history of metabolic allometry and metabolic rates, we complemented the models in a Bayesian framework by running reversible-jump Markov chain Monte Carlo (rjMCMC) analyses (Green 1995; Revell & Harmon 2022; Uyeda *et al.* 2017; Uyeda & Harmon 2014). rjMCMC samples parameter values of the multi-optima OU model to approximate their joint posterior probability distribution (Green 1995; Uyeda & Harmon 2014). It also samples different parameter values for the OU model from their posterior distribution and includes jumps between alternative model parameterization (Green 1995; Uyeda & Harmon 2014). This procedure allows the exploration of evolutionary dynamics across the phylogeny by detecting the number of regime shifts *k*, including their magnitude and location, without specifying a specific number of shifts or their location as a priori assumption for the respective trait (Green 1995; Uyeda & Harmon 2014). Phenotypic shifts in trait optima indicate changes in the adaptive landscape over evolutionary history, suggesting that selection pressures have moved species to different trait optima. If the trait evolves according to a BM process, this would be indicated by very high phylogenetic half‑lives (Cooper *et al.* 2016; Grabowski *et al.* 2023; Hansen 1997). By contrast, low phylogenetic half‑lives represent evidence of trait evolution described by an OU model (Cooper *et al.* 2016; Grabowski *et al.* 2023; Hansen 1997).

We tested three models using the following traits: absolute SMR, mass-independent SMR, and metabolic allometry. The latter model allowed us to investigate the more realistic scenario where the slope and intercept evolve in a coordinated fashion across the phylogeny (Uyeda *et al.* 2017), using SMR as the trait and body mass as the predictor variable (log_10_‑scale). Models were performed in the *bayou* v.2.2.0 *R* package (Uyeda *et al.* 2020). We ran five independent chains for each rjMCMC model. Each chain was performed with 32 million MCMC iterations, sampling every thousand generations (2500 for allometric model), and a burn-in proportion of 30%. We set a conditional Poisson prior to the number of shifts *k*, with a mean equal to 17% of the total number of branches in the full Odonata tree and a maximum number of shifts equal to 50%. For α and σ^2^, we set weakly informative Half Cauchy distributions with 0.1 as scale parameters. We used a normal distribution with the mean of SMR or mass-independent SMR and 1.5 times the standard deviation as relatively informative prior to the optima. For the allometric model, we specified the “OUrepar” model, centered the log_10_ body mass data, and used the mean slope of 0.87 based on the result of the best-supported PGLS model (Supplementary Table S1) with a standard variation of 0.25 as prior for the optima, and a lognormal distribution as prior to the phylogenetic half-life *t_1/2_* (logspace mean: tree height, logspace standard deviation: 0.8) and stationary variance *Vy* (logspace mean: 1.0, logspace standard deviation: 1.5). These relatively informative priors are recommended to avoid fitting models in unrealistic regions of parameter space enabling taxa to track unrealistic distant adaptive optima, facilitating convergence across chains, especially for the phylogenetic half-life (Josef Uyeda, personal communication) (Uyeda *et al.* 2017; Uyeda & Harmon 2014). A uniform prior on the probability of a shift over all branches on the phylogeny and the locations of shifts along branches was placed. Starting values for the parameters of all MCMC model were selected by randomly picking values from their prior distribution. The model parameters α, σ^2^, k, the priors, and the model likelihood (lnL) were considered convergent across chains when the effective sample size was greater than a thousand and the Gelman and Rubin’s R statistic was smaller than 1.05 (< 1.1 for *α*) (Brooks & Gelman 1998; Gelman & Rubin 1992), as determined by the *gelman.R* function in *bayou*. Converging chains were combined to increase the sampled parameter space and effective sample sizes. Posterior probabilities equal to or higher than 0.7 were interpreted as strong statistical support for an adaptive trait shift, as applied in previous studies using *bayou* (Cuff *et al.* 2015; Tonini *et al.* 2020; Uyeda *et al.* 2017; Uyeda & Harmon 2014). For the allometric model, we rerun the analysis using fixed shift locations with all inferred shifts with an average posterior probability of 0.2 or higher across the post-burn-in samples (corresponding to an eightfold increase in the posterior probability over the prior probability) from all converged chains of the first run (Uyeda *et al.* 2017) For this, we set the ”dsb” and ”dk” parameters as ”fixed”. We report and plot the results of the fixed run in the main results rather than of the first run (except for the shift posterior probabilities).

## *Phylogenetic path analysis*

To illuminate the causes of the deviating metabolic allometry in this insect order, we used phylogenetic confirmatory path analysis and directed acyclic graphs (Gonzalez-Voyer & von Hardenberg 2014; von Hardenberg & Gonzalez-Voyer 2012) in the *phylopath* v.1.2.0 *R* package (van der Bijl 2018). Like confirmatory path analysis, phylogenetic path analysis is an extension of multiple regression, but it accounts for the non-independence of observations due to phylogenetic relatedness among species while disentangling the evolutionary relationships between variables (Gonzalez-Voyer & von Hardenberg 2014; von Hardenberg & Gonzalez-Voyer 2012). Specifically, phylogenetic path analysis integrates PGLS models, including a maximum-likelihood estimate of lambda or other evolutionary parameters, with the d‑separation method for path analysis proposed by Shipley (2000b). D‑separation characterizes the minimum set of independence and conditional independence relationships that remain true among all variables of the hypothesized causal model (Gonzalez-Voyer & von Hardenberg 2014; von Hardenberg & Gonzalez-Voyer 2012).

The goodness of fit of the alternative path models was evaluated by d‑sep tests (Shipley 2000a), with significant p‑values of Fisher’s C statistic implying a poor fit of the path model (Gonzalez-Voyer & von Hardenberg 2014; von Hardenberg & Gonzalez-Voyer 2012). Causal models with supported fit (p > 0.05) were compared based on the C statistic Information Criterion corrected for small sample sizes (CICc) and CICc weights (Gonzalez-Voyer & von Hardenberg 2014; von Hardenberg & Gonzalez-Voyer 2012). We accounted for model uncertainty by conditional model averaging, which averages all models with statistical support (ΔCICc ≤ 2) weighted by their relative evidence (i.e., their model likelihood) (van der Bijl 2018; Gonzalez-Voyer & von Hardenberg 2014; von Hardenberg & Gonzalez-Voyer 2012).

We used this approach to explore two sets of alternative causal path models specified by directed acyclic graphs (Gonzalez-Voyer & von Hardenberg 2014; von Hardenberg & Gonzalez-Voyer 2012), allowing the quantification of the direct and indirect effects of body mass, nucleus size, cell size, body volume and body surface area on SMR (all on log_10_‑scale) (Supplementary Fig. S4).

Since the sample sizes of nucleus and cell size were low (20 species), we first fitted nine causal path models without nucleus and cell size, designated as model set 1. Path models in set 1 had a sample size of 43 species and included SMR, body mass, volume and surface area as traits (Supplementary Fig. S4). Causal path structures of the supported models in set 1 were retained and used to test nine additional path models, designated as model set 2. Causal path models in set 2 included nucleus and cell size in addition to the morphological traits of set 1 (Supplementary Fig. S4), with a sample size of 20 species.

**Supplementary Figures**


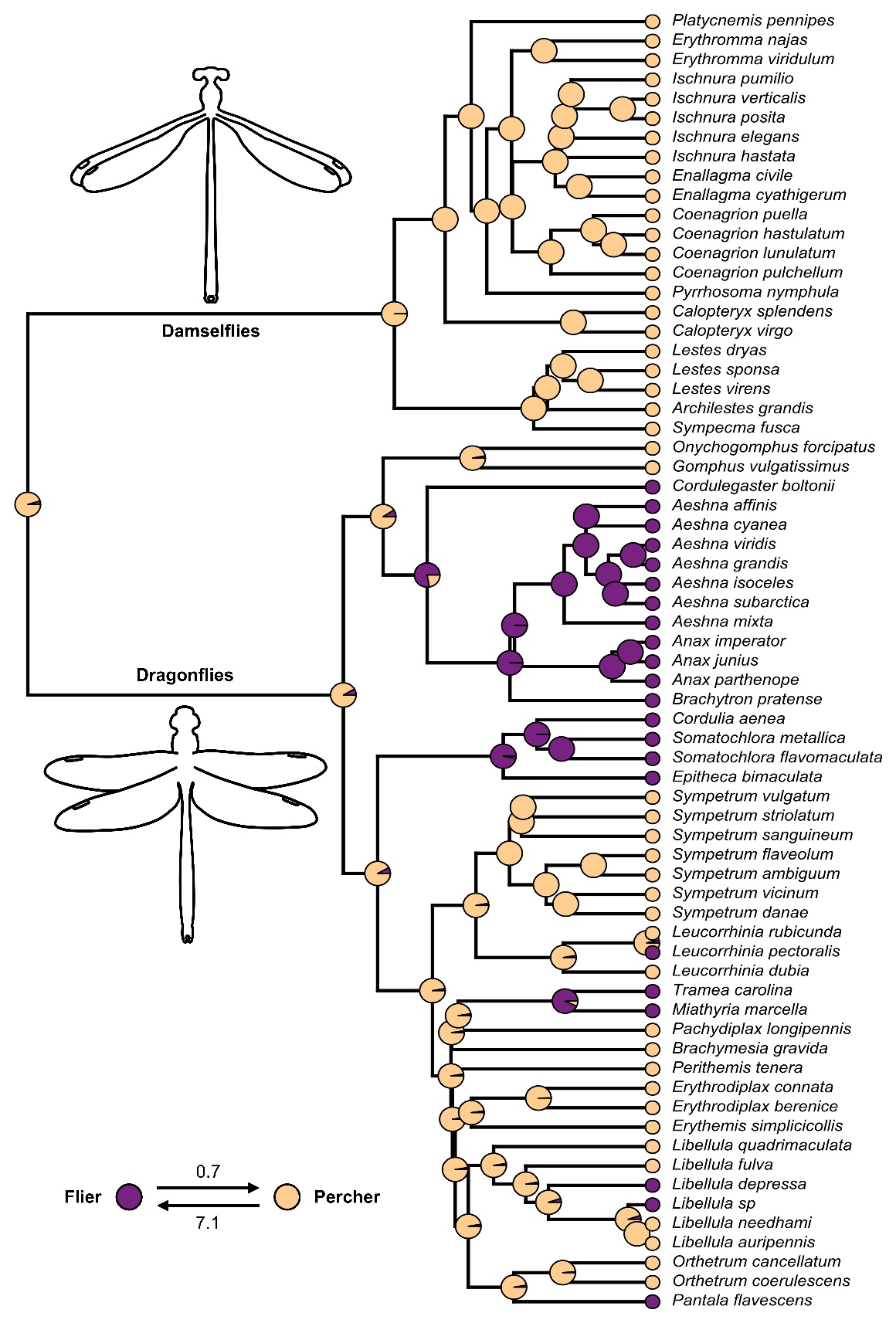


Supplementary Fig. S1: Marginal ancestral state reconstruction and transition rates (Revell & Harmon 2022) for the flight behavior of 67 Odonata species from nine families over 237 million years across the time-calibrated phylogeny. Transition rates (shown in left bottom corner) from flier to percher were much higher than vice versa, with percher being the ancestral state. Reconstruction was performed by model weighting based on four different transition matrices and stochastic character mapping using the *fitMk* function and 701 simulations using the *simmap* function in the *phytools* *R* package (Revell 2024).


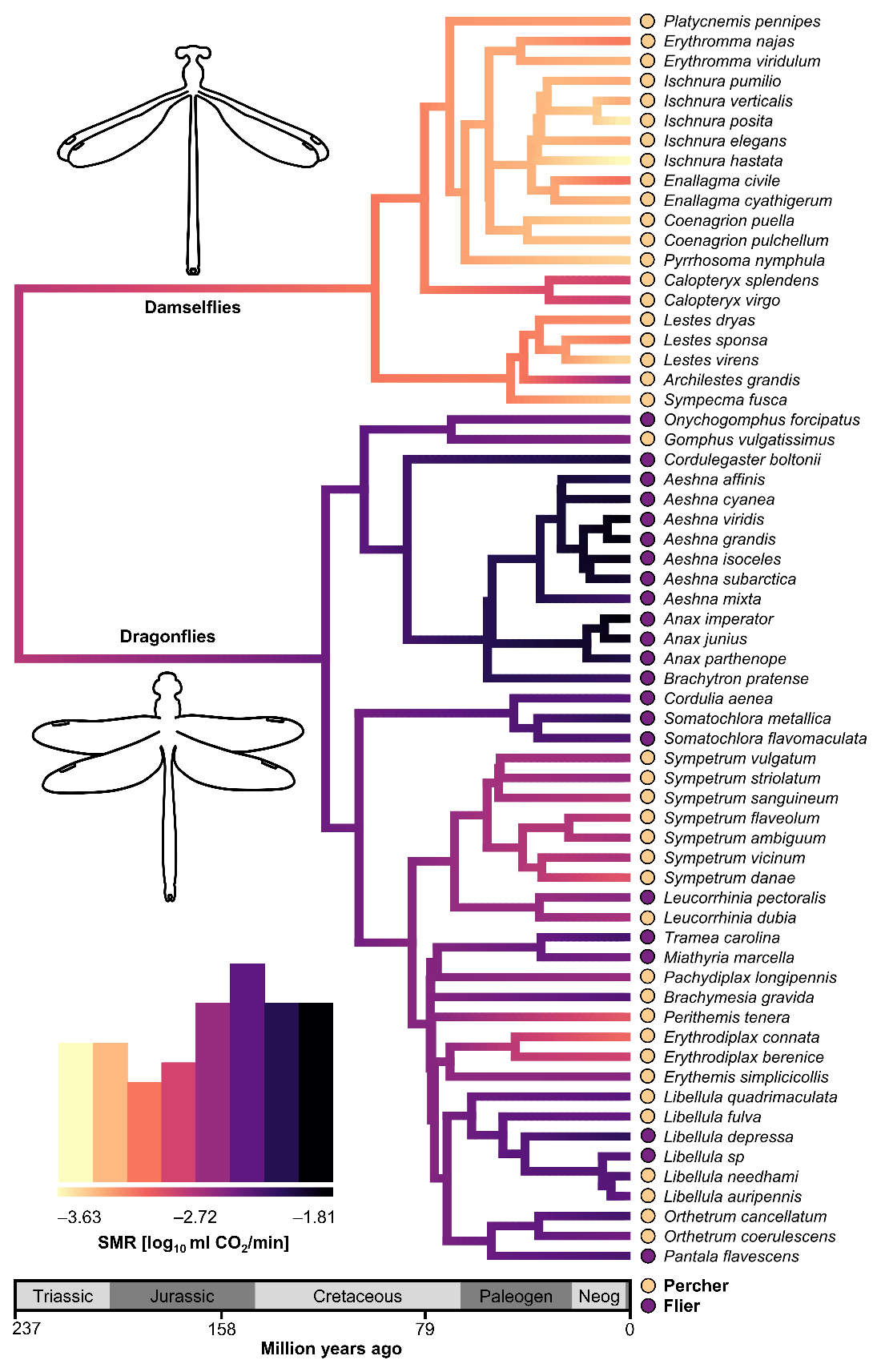


Supplementary Fig. S2: Macroevolution of standard metabolic rate (SMR) for fliers (tan circles) and perchers (purple circles) over 237 million years across the time-calibrated Odonata phylogeny (63 species of nine families). SMR strongly varied across the phylogeny and evolved gradually and in concert with body mass and flight behavior. The color gradient shows the observed trait values at the tips and estimated values along the internal phylogeny. The histogram depicts the distribution of trait values at the tips. The tree was visualized using the *contMap* function in the *phytools* *R* package (Revell 2024), which creates maps based on a Brownian Motion model.


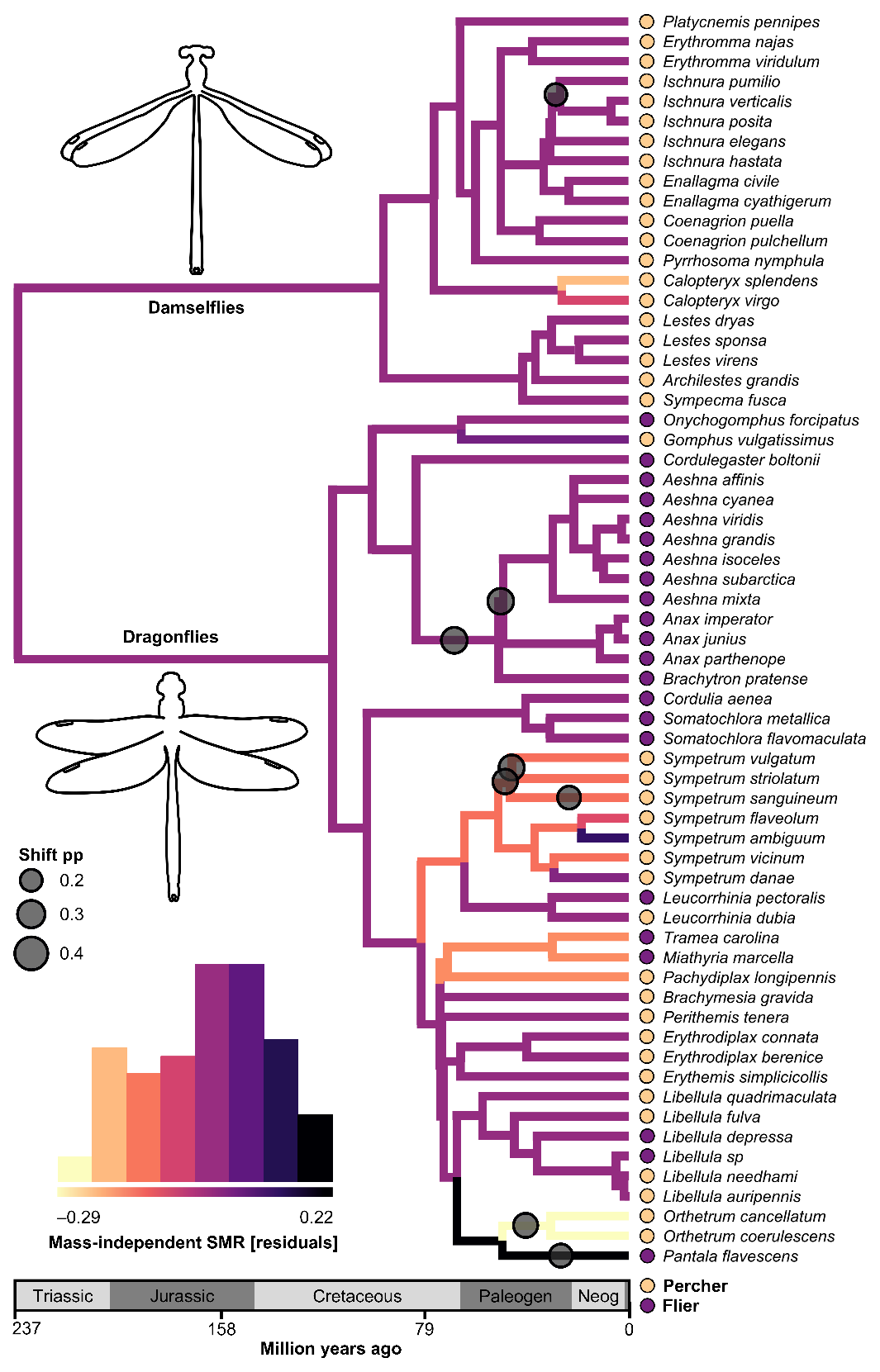


Supplementary Fig. S3: Macroevolution of mass-independent standard metabolic rate (SMR) for fliers (tan tip circles) and perchers (purple tip circles) over 237 million years across the time‑calibrated Odonata phylogeny (63 species) based on a reversible-jumb Markov chain Monte Carlo analysis of the multi-regime Ornstein-Uhlenbeck model (Uyeda & Harmon 2014). Evidence for regime shifts (circles on phylogeny) in mass-independent SMR was limited since all detected shifts had low posterior probabilities (pp). The colors of the tree edges show the mean value of the trait optimum (θ) for that edge from the posterior sample, and the histogram depicts the frequency of θ across the tree edges. Only shifts with pp ≥ 0.2 are shown. We estimated mass-independent SMR using the residuals obtained from phylogenetic size-correction via generalized least squares regression of log_10_ SMR on log_10_ body mass*.* The analysis was performed and plotted in the *bayou R* package (Uyeda *et al.* 2020).
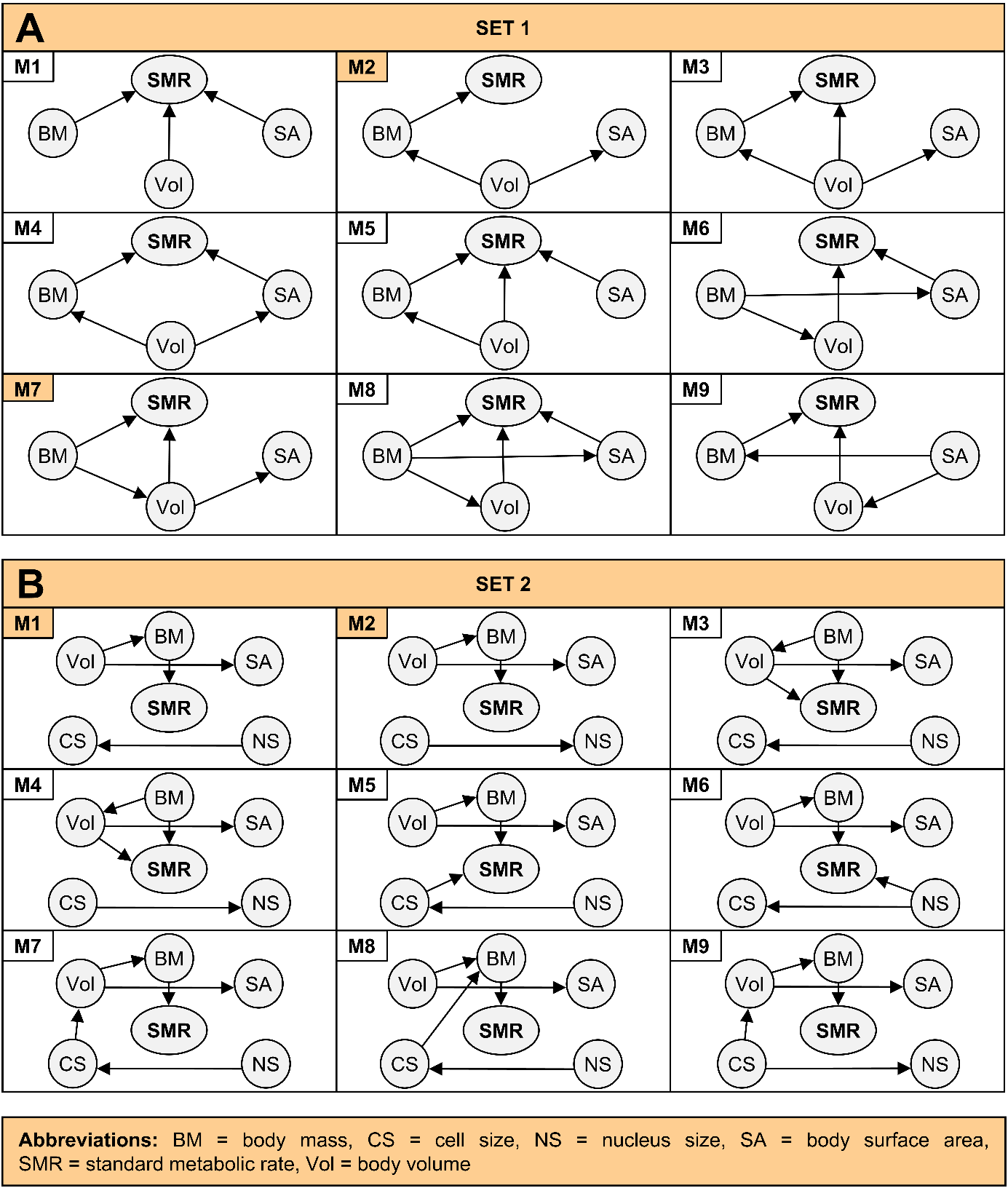


Supplementary Fig. S4: Directed acyclic graphs (DAG) of the alternative phylogenetic path models for model set 1 (A) and set 2 (B) (Garamszegi 2014a; von Hardenberg & Gonzalez-Voyer 2012). Each DAG depicts the hypothesized relationships among morphological and cellular traits (log_10_‑scale) across Odonata. Two sets with nine causal path models each were performed. Set 1 represents the relationship between standard metabolic rate and three morphological traits (body mass, body volume and body surface area) across 43 Odonata species. Statistically supported models of set 1 were retained and incorporated in set 2. Model set 2 additionally included two cellular traits (nucleus and cell size) and the morphological traits of model set 1 across 20 species. Statistically supported models (ΔCICc ≤ 2; Supplementary Table S7) are highlighted in orange and were combined into an average model for each set (Fig. 5). Path models were fitted in *phylopath* (van der Bijl 2018).


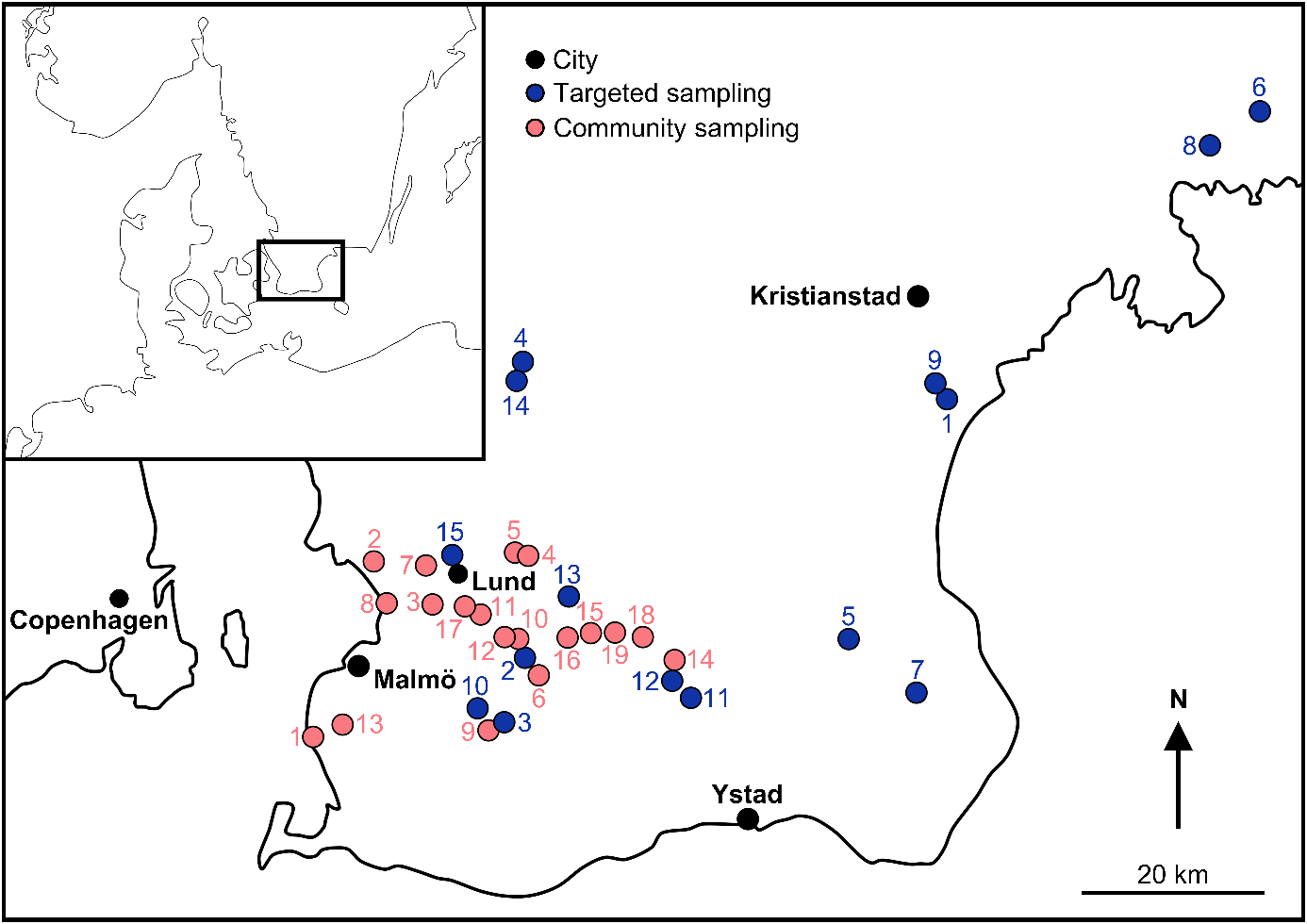


Supplementary Fig. S5: Map of collection sites for Odonata specimens collected in southern Sweden (regions of Skåne and Blekinge). Community sampling locations (rosa dots) were visited regularly over the field season, whereas targeted sampling localities (blue dots) were only visited once or a few times to aim for rare species. Black dots represent cities. Numbers depict locality ID (Supplementary Table S9). The enlarged section of the map is depicted in the top left corner.
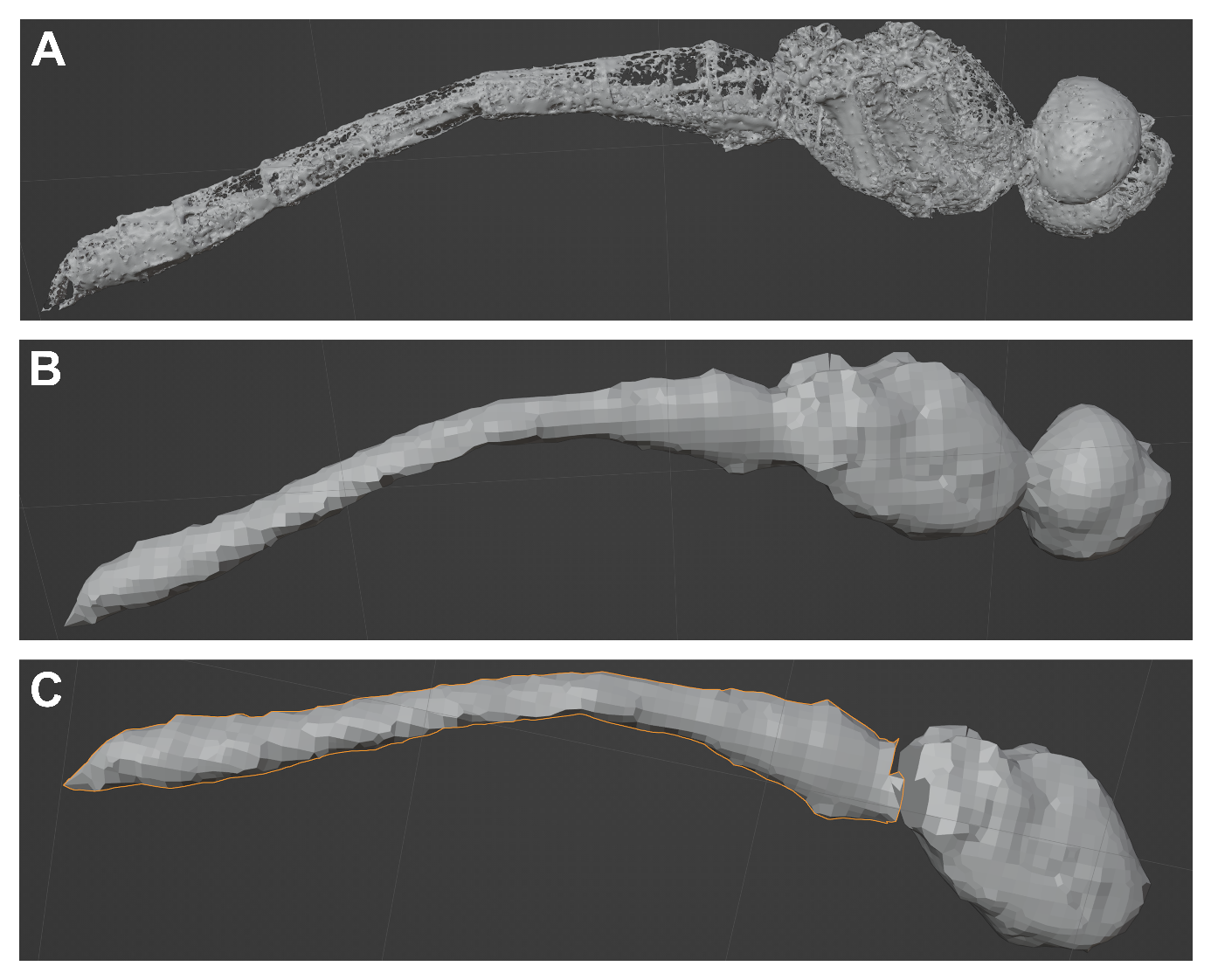


Supplementary Fig. S6: Body volume and body surface area reconstruction of the dragonfly and damselfly specimens in *Blender* based on prior micro-computer tomography (µCT) scans. The reconstruction and the resultant remesh of a male common darter *Sympetrum striolatum* (CT ID: 226) are shown as an example. A) Reconstruction after the successful µCT scan and removal of the noise, the wings, legs and antennae. B) The reconstruction was then remeshed and manually adjusted to approximate the reconstructions. C) The head was removed and the abdomen and thorax were separated, allowing the estimation of the surface area and volume of separately.


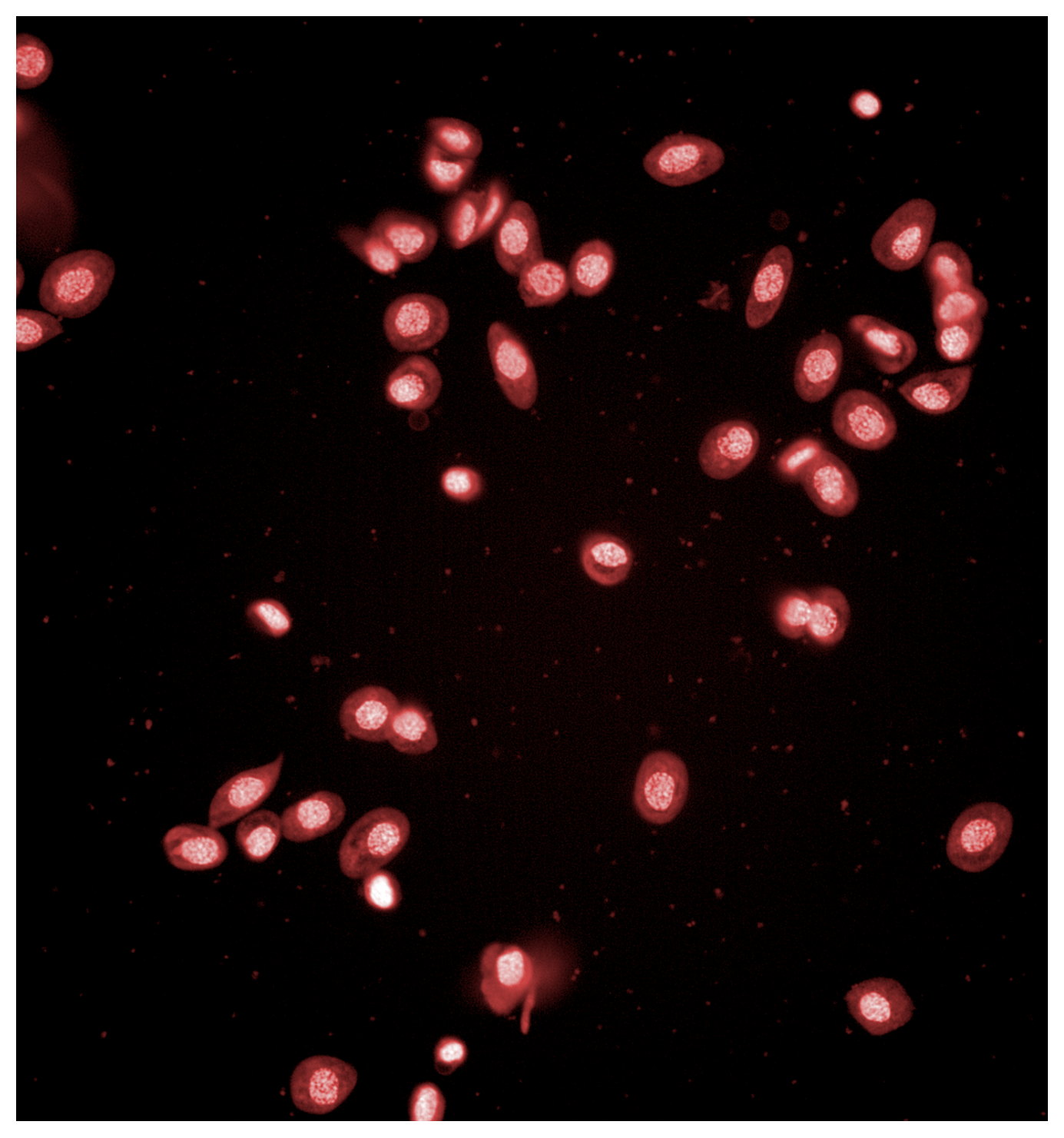


**Supplementary Fig. S7: High-resolution cell imaging using the Operetta CLS High‑Contend Analysis System and the integrated Harmony High‑Content Imaging and Analysis Software to estimate cell and nucleus sizes (area) using hemocytes in dragonflies and damselflies.** This exemplary image shows hemocyte cells of the blue emperor *Anax imperator* (Anisoptera: Aeshnidae). Cells were stained using Anthraquinone dye DRAQ5^TM^.


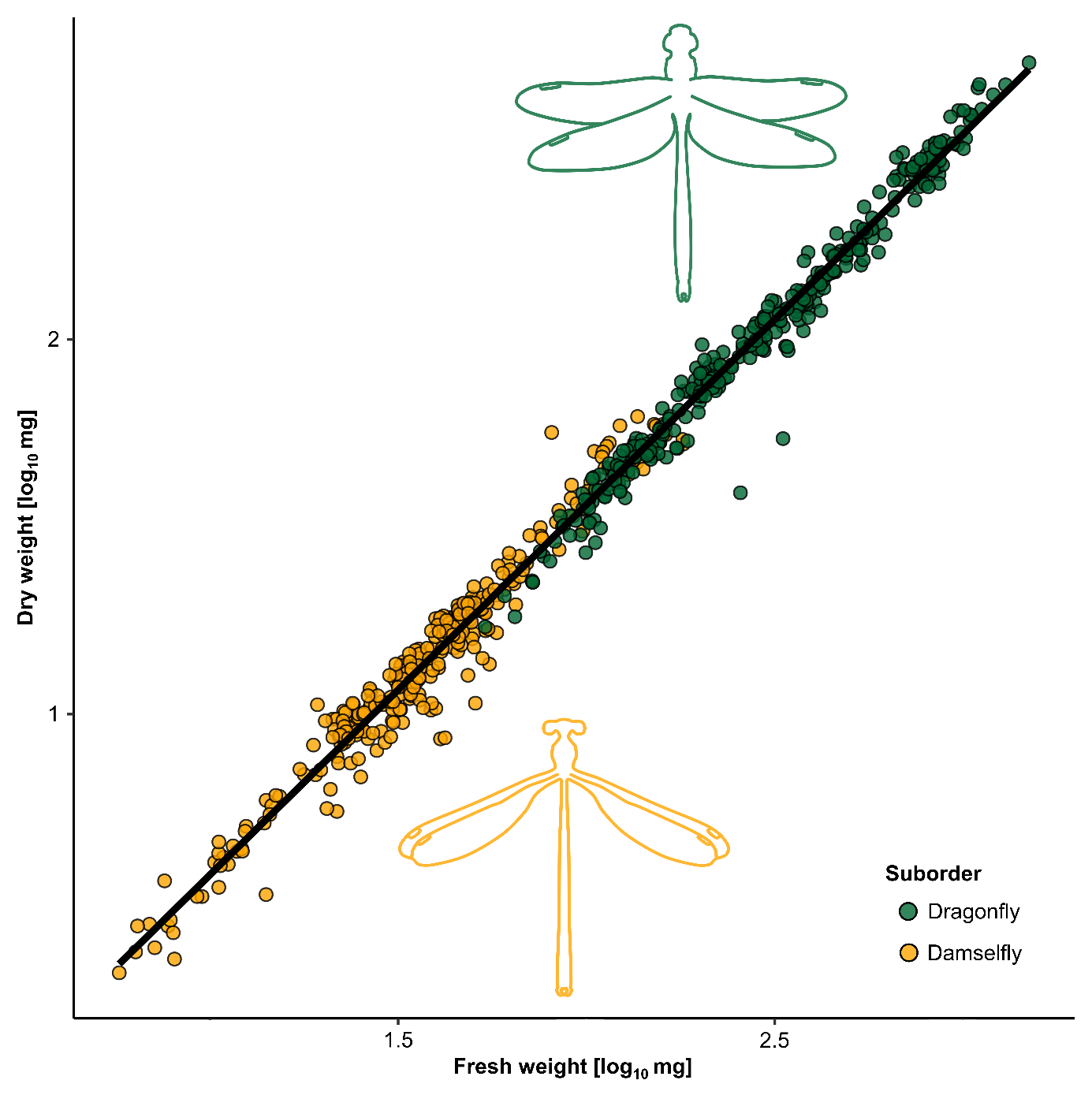


Supplementary Fig. S8: There was a strong correlation (r = 0.99, p < 10^–16^) between log_10_ dry and log_10_ fresh mass across the Odonata order (53 species). Each green or orange dot represents an individual measurement (n = 626) of a dragonfly or damselfly, respectively. The solid line represents the fitted regression line of a linear model with log_10_ dry mass as response and log_10_ fresh mass as predictor variable (slope: 0.99 ± 0.005 standard error, intercept: –4.12 ± 0.01 standard error).


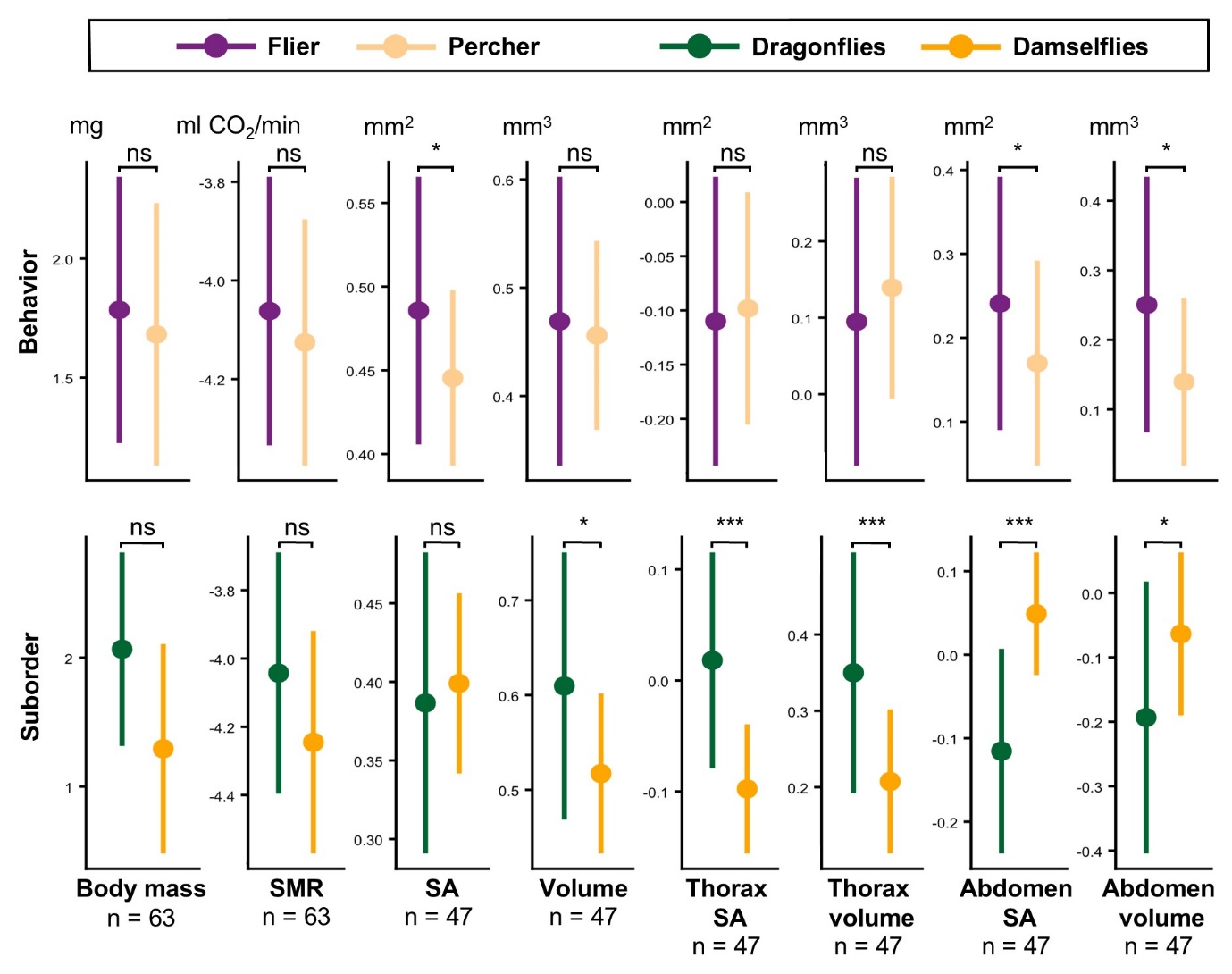


Supplementary Fig. S9: Comparison of body mass, standard metabolic rate (SMR), body surface area (SA), body volume, thorax SA, thorax volume, abdomen SA and abdomen volume between flight behaviors (top) and suborders (bottom) for 47 or 56 Odonata species (n). Large points and bars show estimates of the mean and 95% confidence intervals based on phylogenetic analysis of variance (pANOVA) models fitted with lambda estimation in the *phylolm* *R* package (Ho & Ane 2014). For all body traits, a log_10_‑transformation was conducted. For all body traits except body mass, we accounted for mass differences by including body mass as a covariate. ns indicates no evidence for a significant difference (p > 0.05) and asterisks a significant difference (p ≤ 0.05 *, p ≤ 0.001 ***).


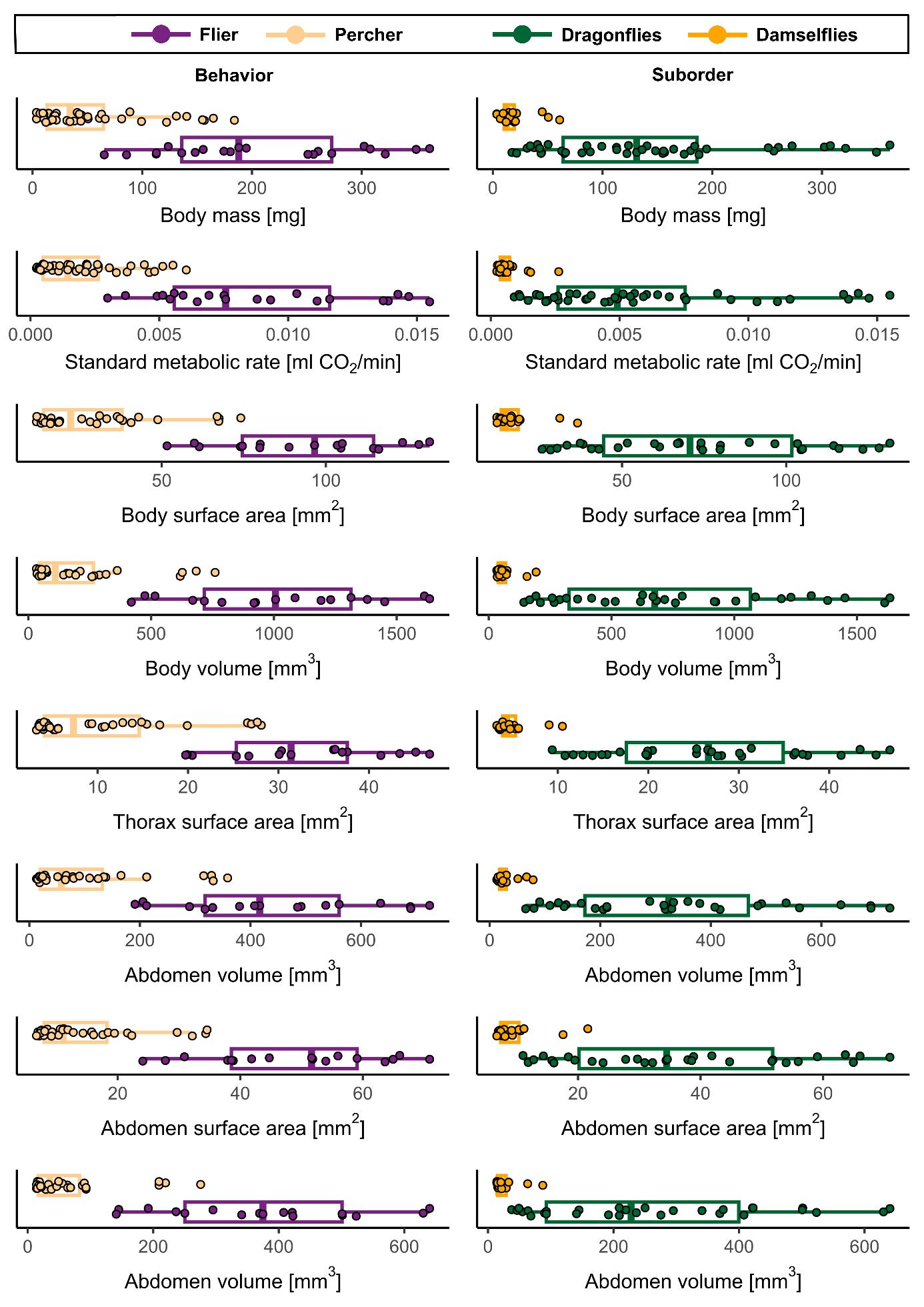


Supplementary Fig. S10: Overview of morphological data, comparing the two flight behaviors (left) and suborders (right). Untransformed data is shown (see Supplementary Fig. S9 for sample sizes).


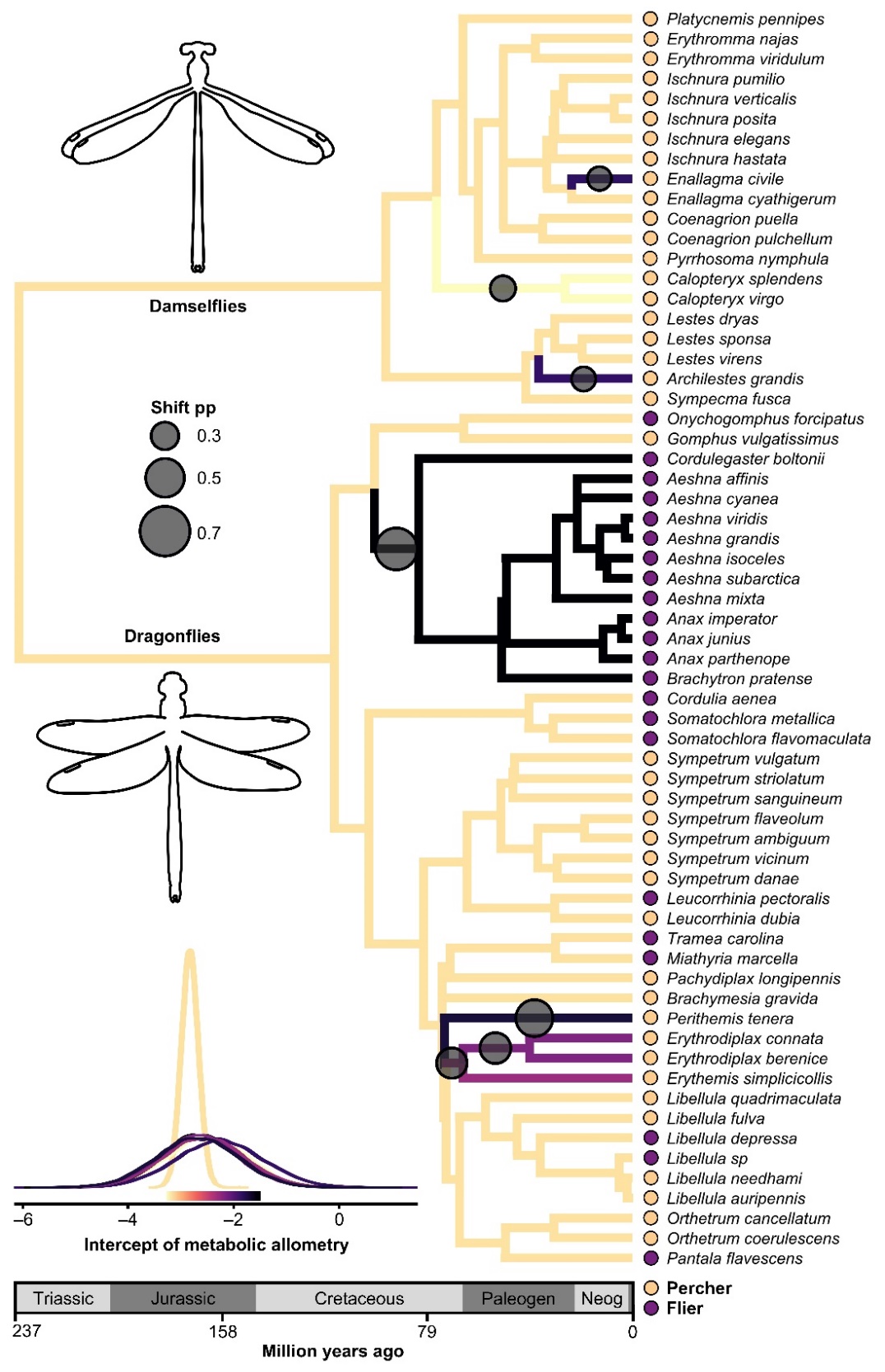


Supplementary Fig. S11: Macroevolutionary dynamics of the slope of the metabolic allometry for perchers (tan tip circles) and fliers (purple tip circles) across the time-calibrated Odonata phylogeny (63 species from nine families) based on a reversible-jumb Markov chain Monte Carlo analysis of the multi-optima Ornstein-Uhlenbeck model (Uyeda & Harmon 2014). Several optima shifts (circles) in allometric slope were identified across the phylogeny, with evidence for weak constraining forces. Circle size on the phylogeny represents the posterior probability (pp) of regime shifts. Only shifts with pp ≥ 0.2 are shown. Colors of tree edges show the mean value of the trait optimum for that edge from the posterior (scale shown in density plot). Density plot depicts the posterior distribution of slope optima for the root state and divergent regimes (colors correspond to tree edge colors). The analysis was performed and plotted in the *bayou* R package (Uyeda *et al.* 2020).


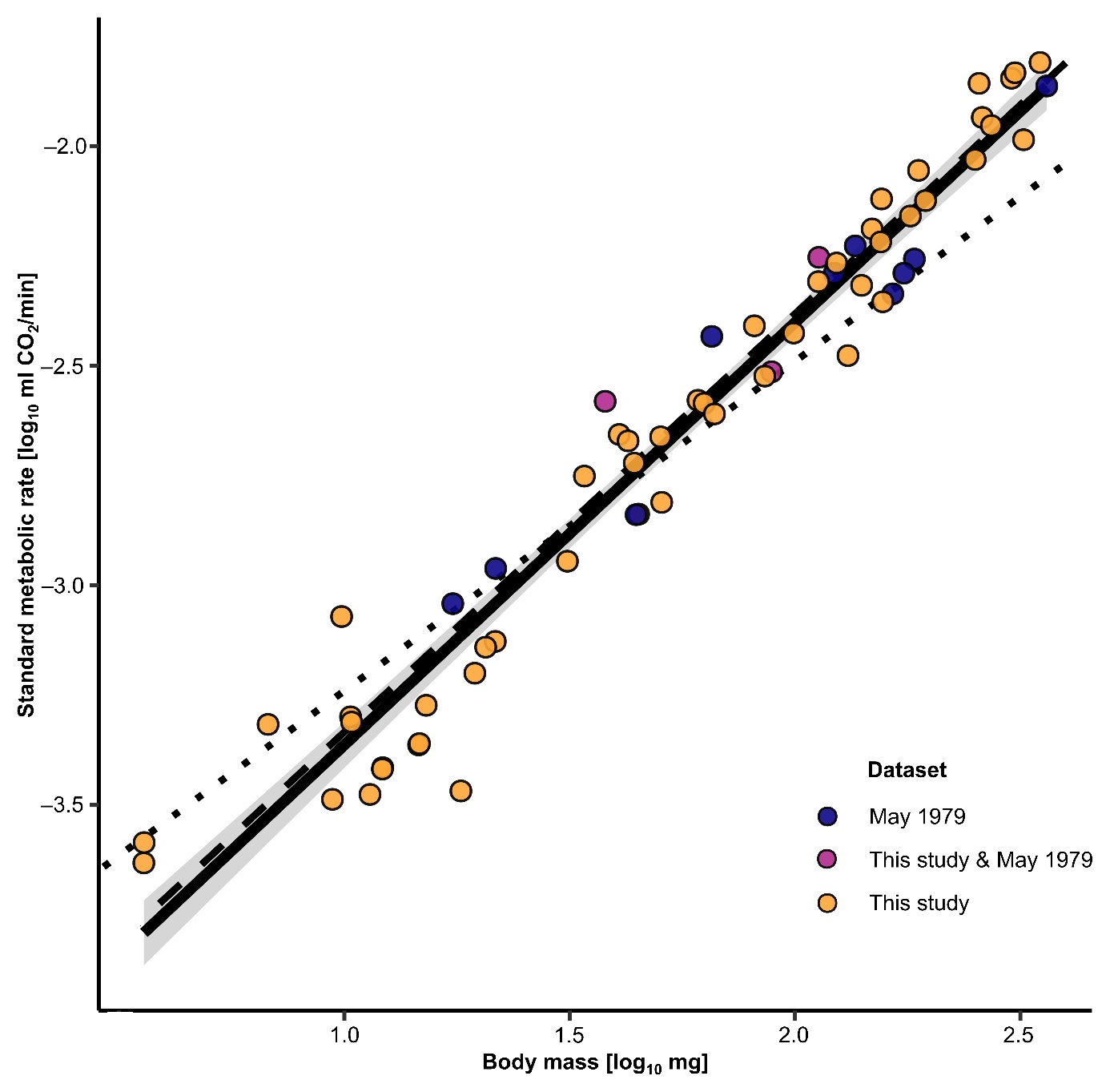


**Supplementary Fig. S11:** Interspecific metabolic allometry for 63 Odonata species across nine families and 30 genera. Similar to Figure 1 in the main text, but with the source of each species indicated by point color.

Supplementary Tables

Supplementary Table S1: Phylogenetic generalized least squares (PGLS) models for the interspecific metabolic allometry of 63 Odonata species

|  | **Model** | **AICc** | **ΔAICc** | **ω** | **Model parameters** | **Intercept** | **Slope** |
| --- | --- | --- | --- | --- | --- | --- | --- |
| **Unweighted** | **PGLS + OU_fR_** | **–98.68** | **––** | **0.21** | **σ^2^ = 2.67 × 10^–4^ α = 0.0076** | **–4.20 (–4.41 – –4.00)** | **0.87 (0.77 – 0.98)** |
|  | **PGLS + OU_rR_** | **–98.52** | **0.16** | **0.20** | **σ^2^ = 2.75 × 10^–4^ α = 0.0082** | **–4.21 (–4.41 – –4.01)** | **0.88 (0.77 – 0.98)** |
|  | **PGLS + δ** | **–98.46** | **0.22** | **0.19** | **σ^2^ = 7.04 × 10^–5^ δ = 3.53** | **–4.21 (–4.41 – –4.02)** | **0.88 (0.78 – 0.98)** |
|  | **PGLS** | **–97.96** | **0.72** | **0.15** | **σ^2^ = 1.46 × 10^–4^** | **–4.16 (–4.44 – –3.88)** | **0.84 (0.73 – 0.95)** |
|  | **PGLS + λ** | **–97.96** | **0.72** | **0.15** | **σ^2^ = 1.55 × 10^–4^ λ = 0.97** | **–4.16 (–4.43 – –3.86)** | **0.84 (0.73 – 0.94)** |
|  | PGLS + κ | –95.91 | 2.77 | 0.05 | σ^2^ = 3.56 × 10^–4^ κ = 0.70 | –4.17 (–4.42 – –3.93) | 0.85 (0.74 – 0.96) |
|  | PGLS + EB | –95.60 | 3.08 | 0.05 | σ^2^ = 2.04 × 10^–4^ b = 0.00 | –4.16 (–4.44 – –3.88) | 0.84 (0.73 – 0.95) |
|  |  |  |  |  |  |  |  |
| **Weighted** | **PGLS + λ** | **–93.92** | **––** | **––** | **––** | **–4.20 (–4.40 – –4.00)** | **0.88 (0.78 – 0.98)** |

Log_10_ standard metabolic rate (SMR; ml CO_2_/min) was used as the response and log_10_ body mass (mg; dry mass) as the predictor variable. The weighted PGLS model incorporated variation in within-species sample size. A strong evolutionary correlation between SMR and body mass (log_10_‑scale) was found (r = 0.89). For the best-supported model, the allometric slope was significantly higher than the traditionally expected slope of 0.67 or 0.75. We fitted multiple maximum-likelihood models with different branch length transformation parameters, including an Early Burst (EB), random root (OU_rR_) and fixed root Ornstein-Uhlenbeck model (OU_fR_). The unweighted models are ranked according to the corrected Akaike Information Criterion scores (AICc) and their differences (ΔAICc), with the best fitting model represented with the highest model weight (ω). Statistically supported models are highlighted in bold (ΔAICc ≤ 2). Estimates of slopes and intercepts are given with 95% confidence intervals (shown in parenthesis). Measurement errors were set to be estimated in all unweighted PGLS models (except in the PGLS_λ_ model since λ and measurement errors cannot be jointly estimated).

**Supplementary Table S2:** Phylogenetic generalized least squares (PGLS) models for the interspecific metabolic allometry of 63 Odonata species using fresh mass instead of dry mass

|  | **Model** | **AICc** | **ΔAICc** | **ω** | **Model parameters** | **Intercept** | **Slope** |
| --- | --- | --- | --- | --- | --- | --- | --- |
| **Unweighted** | **PGLS + δ** | **–98.38** | **––** | **0.25** | **σ^2^ = 5.80 × 10^–5^ δ = 5.35** | **–4.65 (–4.86 – –4.44)** | **0.90 (0.81 – 0.99)** |
|  | **PGLS + OU_fR_** | **–98.38** | **0.00** | **0.25** | **σ^2^ = 3.22 × 10^–4^ α = 0.011** | **–4.63 (–4.85 – –4.40)** | **0.89 (0.79 – 0.99)** |
|  | **PGLS + OU_rR_** | **–98.33** | **0.05** | **0.25** | **σ^2^ = 3.27 × 10^–4^ α = 0.012** | **–4.63 (–4.85 – –4.41)** | **0.89 (0.80 – 0.99)** |
|  | PGLS | –96.30 | 2.09 | 0.09 | σ^2^ = 1.21 × 10^–4^ | –4.53 (–4.83 – –4.24) | 0.84 (0.74 – 0.95) |
|  | PGLS + λ | –96.30 | 2.09 | 0.09 | σ^2^ = 1.32 × 10^–4^ λ = 0.91 | –4.53 (–4.83 – –4.23) | 0.84 (0.73 – 0.95) |
|  | PGLS + κ | –94.68 | 3.70 | 0.04 | σ^2^ = 4.60 × 10^–4^ κ = 0.52 | –4.58 (–4.85 – –4.31) | 0.86 (0.75 – 0.97) |
|  | PGLS + EB | –93.39 | 4.45 | 0.03 | σ^2^ = 1.21 × 10^–4^ b = 0.00 | –4.53 (–4.83 – –4.23) | 0.84 (0.73 – 0.95) |
|  |  |  |  |  |  |  |  |
| **Weighted** | **PGLS + λ** | **–97.37** | **––** | **––** | **––** | **–4.67 (–4.90 – –4.44)** | **0.90 (0.81 – 1.00)** |

Log_10_ standard metabolic rate (SMR; ml CO_2_/min) was used as the response and log_10_ fresh mass (mg) as the predictor variable. The weighted PGLS model incorporated variation in within-species sample size. For all statistically supported models, a highly significant evolutionary correlation between SMR and fresh mass (log_10_‑scale) was found (r = 0.88), with an allometric slope substantially higher than the traditionally expected slope of 0.67 or 0.75. We fitted multiple maximum-likelihood models differing in their branch length transformation parameters, including an Early Burst (EB), random root (OU_rR_) and fixed root Ornstein-Uhlenbeck model (OU_fR_). The unweighted models are ranked according to the corrected Akaike Information Criterion scores (AICc) and their differences (ΔAICc), with the best fitting model represented with the highest model weight (ω). Statistically supported models are highlighted in bold (ΔAICc ≤ 2). Estimates of slopes and intercepts are given with 95% confidence intervals (shown in parenthesis). Measurement errors were set to be estimated in all unweighted PGLS models (except in the PGLS_λ_ model since λ and measurement errors cannot be jointly estimated).

**Supplementary Table S3:** Phylogenetic generalized least squares (PGLS) models for the interspecific metabolic allometry of 34 Odonata species after excluding species with sample sizes smaller than ten

|  | **Model** | **AICc** | **ΔAICc** | **ω** | **Model parameters** | **Intercept** | **Slope** |
| --- | --- | --- | --- | --- | --- | --- | --- |
| **Unweighted** | **PGLS + κ** | **–65.06** | **––** | **0.88** | **σ^2^ = 2.43 × 10^–3^ κ = 1.0 × 10^–6^** | **–4.21 (–4.40 – –4.02)** | **0.87 (0.77 – 0.96)** |
|  | PGLS + λ | –58.94 | 6.12 | 0.04 | σ^2^ = 1.12 × 10^–4^ λ = 1.03 | –4.13 (–4.42 – –3.89) | 0.83 (0.72 – 0.95) |
|  | PGLS | –58.63 | 6.43 | 0.04 | σ^2^ = 1.02 × 10^–4^ | –4.15 (–4.38 – –3.89) | 0.84 (0.73 – 0.96) |
|  | PGLS + OU_fR_ | –56.37 | 8.68 | 0.01 | σ^2^ = 1.27 × 10^–4^ α = 0.0031 | –4.16 (–4.40 – –3.93) | 0.85 (0.74 – 0.97) |
|  | PGLS + δ | –56.23 | 8.82 | 0.01 | σ^2^ = 6.85 × 10^–5^ δ = 1.70 | –4.16 (–4.39 – –3.93) | 0.85 (0.74 – 0.96) |
|  | PGLS + EB | –55.87 | 9.19 | 0.01 | σ^2^ = 1.02 × 10^–4^ b = 0.00 | –4.13 (–4.39 – –3.87) | 0.83 (0.72 – 0.95) |
|  | PGLS + OU_rR_ | –55.79 | 9.27 | 0.01 | σ^2^ = 1.44 × 10^–4^ α = 0.0048 | –4.18 (–4.40 – –3.97) | 0.87 (0.75 – 0.98) |
|  |  |  |  |  |  |  |  |
| **Weighted** | **PGLS + λ** | **–58.63** | **––** | **––** | **––** | **–4.14 (–4.46 – –4.18)** | **0.93 (0.89 – 0.96)** |

Log_10_ standard metabolic rate (SMR; ml CO_2_/min) was used as the response and log_10_ body mass (mg; dry mass) as the predictor variable. The weighted PGLS model incorporated variation in within-species sample size. For the statistically supported model, the allometric slope was substantially higher than the traditionally expected slope of 0.67 or 0.75. We fitted multiple maximum-likelihood models differing in their branch length transformation parameters, including an Early Burst (EB), random root (OU_rR_) and fixed root Ornstein-Uhlenbeck model (OU_fR_). The unweighted models are ranked according to the corrected Akaike Information Criterion scores (AICc) and their differences (ΔAICc), with the best fitting model represented with the highest model weight (ω). Statistically supported models are highlighted in bold (ΔAICc ≤ 2). Estimates of slopes and intercepts are provided with 95% confidence intervals (shown in parenthesis). Measurement errors were set to be estimated in all unweighted PGLS models (except in the PGLS_λ_ model since λ and measurement errors cannot be jointly estimated).

**Supplementary Table S4:** Evidence for curvilinearity in metabolic allometry in Odonata (Kolokotrones *et al.* 2010)

| **Dataset** | **ΔAICc** | **Quadratic coefficient** | **p-value** |
| --- | --- | --- | --- |
| Full | 0.75 | 0.11 (0.06 SE) | 0.07 |
| Fresh instead of dry mass | 3.53 | 0.14 (0.05 SE) | 0.02 |
| Excluding species with sample size > 10 | 0.34 | 0.13 (0.06 SE) | 0.03 |

We fitted curvilinear PGLS_λ_ that included a quadratic coefficient and compared them to linear PGLS_λ_ using ΔAICc.

**Supplementary Table S5:** Phylogenetic analysis of covariance models (Smaers & Rohlf 2016) to detect Odonata families representing a significant outlier in the metabolic allometry

| **Family** | **Number of species** | **F-value** | **p-value** |
| --- | --- | --- | --- |
| Aeshnidae | 11 | 0.81 | 0.45 |
| Calopterygidae | 2 | 0.06 | 0.94 |
| **Coenagrionidae** | **12** | **4.18** | **0.02** |
| Corduliidae | 3 | 0.39 | 0.68 |
| Gomphidae | 2 | 0.90 | 0.41 |
| Lestidae | 5 | 2.92 | 0.06 |
| Libellulidae | 26 | 0.19 | 0.82 |

F‑ratio tests were used to investigate if a more complex PGLS_λ_ model that included a separate intercept and slope for a specific family fitted the data better than a PGLS_λ_ that assumed equal slopes and intercepts for all species (Smaers & Rohlf 2016). Families represented by only one species were excluded. Significant p‑values (p ≤ 0.05) are shown in bold and indicate that the family has a substantially different metabolic scaling relationship (slope and intercept) after accounting for variation in the covariates. Models were fitted in the *evomap* *R* package (Smaers & Mongle 2018).

Supplementary Table S6: Results of phylogenetic path analyses (Garamszegi 2014a; von Hardenberg & Gonzalez-Voyer 2012) for models (set 1 and set 2)

|  | **Path model** | **k** | **q** | **Fisher’s C statistic** | **p value** | **CICc** | **ΔCICc** | **Model weight ω** |
| --- | --- | --- | --- | --- | --- | --- | --- | --- |
| **Set 1** | **M7** | **2** | **8** | **1.95** | **0.75** | **22.2** | **––** | **0.45** |
|  | **M2** | **3** | **7** | **6.17** | **0.40** | **23.4** | **1.19** | **0.25** |
|  | M4 | 2 | 8 | 4.25 | 0.37 | 24.5 | 2.31 | 0.14 |
|  | M3 | 2 | 8 | 4.84 | 0.30 | 25.1 | 2.90 | 0.11 |
|  | M5 | 1 | 9 | 3.40 | 0.18 | 26.9 | 4.67 | 0.04 |
|  | M9 | 2 | 8 | 9.36 | 0.05 | 29.6 | 7.41 | 0.01 |
|  | M8 | 1 | 9 | 72.09 | < 0.001 | 95.5 | 73.36 | 0.00 |
|  | M6 | 2 | 8 | 98.92 | < 0.001 | 119.2 | 99.98 | 0.00 |
|  | M1 | 3 | 7 | 216.26 | < 0.001 | 233.5 | 211.28 | 0.00 |
|  |  |  |  |  |  |  |  |  |
| **Set 2** | **M1** | **11** | **10** | **18.2** | **0.70** | **65.7** | **––** | **0.55** |
|  | **M2** | **11** | **10** | **18.6** | **0.67** | **66.1** | **0.47** | **0.43** |
|  | M9 | 10 | 11 | 14.6 | 0.80 | 74.3 | 8.63 | 0.01 |
|  | M7 | 10 | 11 | 15.0 | 0.77 | 74.8 | 9.10 | 0.01 |
|  | M8 | 10 | 11 | 17.2 | 0.64 | 76.9 | 11.29 | 0.00 |
|  | M5 | 10 | 11 | 17.2 | 0.64 | 76.9 | 11.29 | 0.00 |
|  | M3 | 10 | 11 | 17.5 | 0.62 | 77.2 | 11.58 | 0.00 |
|  | M4 | 10 | 11 | 17.9 | 0.59 | 77.7 | 12.00 | 0.00 |
|  | M6 | 10 | 11 | 18.1 | 0.58 | 77.8 | 12.12 | 0.00 |

Shown are the number of tested conditional independencies (k) and model parameters (q), Fisher’s C statistic and its p values for the two sets of hypothesized alternative causal path models (Supplementary Fig. S4) (Garamszegi 2014a; von Hardenberg & Gonzalez-Voyer 2012). Models with poor model fit (p ≤ 0.05 of C statistic) were rejected. Models were ranked according to the corrected C statistic Information Criterion scores (CICc) and their differences (ΔCICc), with the best fitting model represented with the highest model weight ω. Statistically best-supported path models (ΔCICc ≤ 2) are highlighted in bold. Data for 43 or 19 Odonata species were used in model sets 1 and 2, respectively. Path models were fitted in *phylopath* (van der Bijl 2018).Supplementary Table S7: Comparison of models for the evolution of standard metabolic rate (log_10_ ml CO_2_/min) and mass-independent standard metabolic rate (residuals) across the phylogeny of 63 Odonata species

|  | **Model** | **logL** | **k** | **AICc** | **ΔAICc** | **w** |
| --- | --- | --- | --- | --- | --- | --- |
| **Standard metabolic rate** | **_mo_OU behavior** | **7.95** | **4** | **–7.21** | **––** | **0.51** |
|  | **_mr_BM suborder** | **6.01** | **3** | **–5.61** | **1.60** | **0.23** |
|  | _mr_BM behavior | 5.19 | 3 | –3.97 | 3.25 | 0.10 |
|  | _sr_BM | 3.89 | 2 | –3.58 | 3.64 | 0.08 |
|  | _mo_OU suborder | 5.60 | 4 | –2.51 | 4.70 | 0.05 |
|  | _so_OU | 3.89 | 3 | –1.37 | 5.84 | 0.03 |
|  |  |  |  |  |  |  |
| **Mass-independent standard metabolic rate** | **_mo_OU behavior** | **45.76** | **4** | **–82.83** | **––** | **0.31** |
|  | **_so_OU** | **44.38** | **3** | **–82.36** | **0.47** | **0.24** |
|  | **_sr_BM** | **42.95** | **2** | **–81.69** | **1.14** | **0.18** |
|  | **_mr_BM suborder** | **43.70** | **3** | **–80.99** | **1.84** | **0.12** |
|  | _mo_OU suborder | 44.43 | 4 | –80.18 | 2.66 | 0.08 |
|  | _mr_BM behavior | 43.05 | 3 | –79.70 | 3.14 | 0.06 |

A single-rate Brownian motion (_sr_BM) model was compared to the fit of multi-rate BM (_mr_BM), single-optimum Ornstein-Uhlenbeck (OU) and multi-optima OU models (Revell & Harmon 2022). For the multi-rate BM and multi-optima OU models, we investigated if there was evidence for different regime optima between suborders (dragonflies and damselflies) or flight behaviors (fliers and perchers). All models were fitted using maximum-likelihood estimation in *OUwie* (Beaulieu & O’Meara 2022), with standard errors of the mean incorporated as measurement errors. We estimated mass-independent trait values using the residuals obtained from phylogenetic size‑correction via generalized least squares regression of log_10_ SMR on log_10_ body mass. Models are ranked according to the corrected Akaike Information Criterion (AICc) and their differences (ΔAICc), with the best fitting model represented with the highest model weight (ω). Statistically supported models (ΔAICc < 2) are highlighted in bold.

Supplementary Table S8: Results of the reversible-jumb Markov chain Monte Carlo (rjMCMC) models based on multi‑optima Ornstein-Uhlenbeck models (Uyeda *et al.* 2017; Uyeda & Harmon 2014).

| **Model** | **Model parameter** | **Parameter mean** | **Parameter lower HPD** | **Parameter higher HPD** |  | **Shift position  (descendants of clade)** | **Shift pp** | **Shift θ (magnitude ± SE)** |
| --- | --- | --- | --- | --- | --- | --- | --- | --- |
| **SMR** | **lnL** | 33.59 | 3.18 | 43.89 |  | *Archilestes grandis* | 0.94 | –2.27 **±** 0.0019 |
|  | **prior** | –45.79 | –61.29 | –30.98 |  | All Calopterygidae | 0.90 | –2.75 **±** 0.0009 |
|  | **t_1/2_** | 1.13 | 3.8×10^5^ | 0.25 |  | *Perithemis tenera* | 0.87 | –3.08 **±** 0.0126 |
|  | **σ^2^** | 0.02 | 2.7×10^–4^ | 8.0×10^–2^ |  | All *Erythrodiplax* | 0.81 | –3.00 **±** 0.0086 |
|  | **k** | 10.80 | 7.00 | 15.00 |  | All *Sympetrum* & *Leucorrhinia* | 0.77 | –3.35 **±** 0.0042 |
|  | **θ_Root_** | –2.79 | –3.41 | –2.25 |  | All Aeshnidae & Cordulegastridae | 0.76 | –2.31 **±** 0.0047 |
|  | **θ_All_** | –2.66 | –– | –– |  | All Zygoptera | 0.52 | –3.35 **±** 0.0076 |
|  |  |  |  |  |  | All Anisoptera | 0.45 | –2.31 **±** 0.0090 |
|  |  |  |  |  |  | *Pachydiplax* *longipennis* | 0.13 | –2.69 **±** 0.0388 |
|  |  |  |  |  |  | *Enallagma civile* | 0.12 | –2.65 **±** 0.0624 |
|  |  |  |  |  |  | *Sympetrum danae* | 0.12 | –3.08 **±** 0.0502 |
|  |  |  |  |  |  | *Aeshna mixta* | 0.11 | –2.52 **±** 0.0624 |
|  |  |  |  |  |  | *Lestes sponsa* | 0.10 | –2.70 **±** 0.0697 |
|  |  |  |  |  |  | All *Sympetrum* | 0.10 | –2.75 **±** 0.0228 |
|  |  |  |  |  |  |  |  |  |
| **miSMR** | **lnL** | 50.62 | 41.11 | 43.89 |  | All Aeshnidae | 0.28 | 0.18 **±** 0.0009 |
|  | **prior** | –30.50 | –43.86 | –17.73 |  | All *Aeshna* & *Anax* | 0.27 | 0.20 **±** 0.0005 |
|  | **t_1/2_** | 0.66 | 1.6×10^5^ | 0.14 |  | *S. vulgatum* & *S. striolatum* | 0.26 | –0.24 **±** 0.0008 |
|  | **σ^2^** | 0.01 | 9.2×10^–5^ | 7.2×10^–2^ |  | *S. vulgatum.*, *S. striol. & S. sang.* | 0.26 | –0.18 **±** 0.0007 |
|  | **k** | 10.45 | 5.00 | 16.00 |  | All *Orthetrum* | 0.25 | –0.14 **±** 0.0007 |
|  | **θ_Root_** | 0.03 | –2.07 | 0.09 |  | *Sympetrum vulgatum* | 0.23 | –0.28 **±** 0.0011 |
|  | **θ_All_** | 0.04 | –– | –– |  | *Pantala flavescens* | 0.22 | 0.22 **±** 0.0011 |
|  |  |  |  |  |  | *I. pumilio*, *I. verticalis* & *I. posita* | 0.21 | 0.20 **±** 0.0008 |
|  |  |  |  |  |  | All *Sympetrum* | 0.18 | –1.11 **±** 0.0008 |
|  |  |  |  |  |  | *Orthetrum coerulescens* | 0.15 | –1.43 **±** 0.0015 |
|  |  |  |  |  |  | *Pachydiplax longipennis* | 0.14 | –1.12 **±** 0.0013 |
|  |  |  |  |  |  | *Perithemis tenera* | 0.14 | –1.12 **±** 0.0014 |
|  |  |  |  |  |  | All *Ischnura* | 0.13 | 0.16 **±** 0.0010 |
|  |  |  |  |  |  | *Sympetrum danae* | 0.13 | 0.14 **±** 0.0016 |
|  |  |  |  |  |  | *I. pum.*, *I. vert.*, *I. posit & I. eleg.* | 0.12 | 0.17 **±** 0.0011 |
|  |  |  |  |  |  | All *Aeshna* | 0.12 | 0.20 **±** 0.0011 |
|  |  |  |  |  |  | *T. carolina*, *M. marcel.* & *P. lon.* | 0.12 | –0.09 **±** 0.0010 |
|  |  |  |  |  |  | *Ischnura pumilio* | 0.11 | 0.20 **±** 0.0017 |
|  |  |  |  |  |  | *Cordulegaster boltonii* | 0.11 | –0.06 **±** 0.0015 |
|  |  |  |  |  |  | All *Orthetrum* & *P. flavescens* | 0.10 | 0.04 **±** 0.0021 |
|  |  |  |  |  |  | *Tramea carolina* | 0.10 | –0.09 **±** 0.0019 |
|  |  |  |  |  |  | All *Lestes* & *Archilestes grandis* | 0.10 | 0.13 **±** 0.0011 |
|  |  |  |  |  |  | *Lestes sponsa* & *Lestes virens* | 0.10 | 0.16 **±** 0.0014 |
|  |  |  |  |  |  |  |  |  |
| **Met Allo** | **lnL** | 18.74 | 14.56 | 22.28 |  | All Aeshnidae & Cordulegastridae | 0.73 | –2.62 **±** 0.0029 |
|  | **prior** | –25.18 | –31.54 | –19.53 |  | *Perithemis tenera* | 0.47 | –2.69 **±** 0.0031 |
|  | **t_1/2_** | 810.28 | 141.26 | 1.8×10^3^ |  | All *Erythrodiplax* & *Erythemis* | 0.36 | –2.66 **±** 0.0030 |
|  | **σ^2^** | 0.30 | 0.06 | 0.71 |  | *Erythemis simplicicollis* | 0.35 | –2.57 **±** 0.0031 |
|  | **k** | 6.49 | 2.00 | 10.00 |  | All *Calopteryx* | 0.26 | –2.51 **±** 0.0031 |
|  | **θ_Root intercept_** | –2.83 | –3.15 | –2.50 |  | *Enallagma civile* | 0.23 | –2.62 **±** 0.0031 |
|  | **θ_Root slope_** | –0.09 | –0.21 | 0.03 |  | *Archilestes grandis* | 0.21 | –2.26 **±** 0.0033 |
|  | **θ_All_ _intercept_** | –2.60 | –– | –– |  |  |  |  |
|  | **θ_All_ _slope_** | 0.77 | –– | –– |  |  |  |  |

Shown are the model and shift parameters for three rjMCMC models performed in *bayou* (Uyeda *et al.* 2020), namely for standard metabolic rate (SMR; log_10_ ml CO_2_/min), mass-independent SMR (miSMR; residuals) and metabolic allometry (Met Allo). Mean model parameters are depicted with lower and higher 95% highest posterior density interval (HPD). Shifts with posterior probabilities (pp) higher than 0.7 were considered statistically supported. Shift positions and the magnitude of the trait optima (θ) are also depicted in Fig. 3 and Supplementary Fig. S3. Models were fitted in the *bayou R* package (Uyeda *et al.* 2020)**.** σ^2^ = evolutionary rate, k = number of shifts, *I. eleg.*=*Ischnura elegans*, *I. pum.*=*Ischnura pumilio*, *I. posit.*=*Ischnura posita*, *I. vert.*=*Ischnura verticalis*, *M. marcel* = *Miathyria marcella*, *P. lon.*=*Pachydiplax longipennis*, *S. sang.* *=* *Sympetrum sanguineum*, *S. striol* = *Sympetrum* *striolatum*, SE = standard error, t_1/2_ = phylogenetic half-life (million years).

**Supplementary Table S9:** Collection localities in Sweden for the dragonfly and damselfly individuals used in our study

|  | **ID** | **Locality** | **Municipality** | **County** | **Latitude** | **Longitude** |
| --- | --- | --- | --- | --- | --- | --- |
| **Community sampling** | 1 | Bunkeflostrand | Malmö | Skåne | 55.538643N | 12.923311E |
|  | 2 | Borgeby | Lomma | Skåne | 55.738264N | 13.047567E |
|  | 3 | Flackarp | Lund | Skåne | 55.689511N | 13.167893E |
|  | 4 | Flyinge 30 A1 | Eslöv | Skåne | 55.746578N | 13.359953E |
|  | 5 | Flyinge 30 A3 | Eslöv | Skåne | 55.750340N | 13.339288E |
|  | 6 | Genarp | Lund | Skåne | 55.608660N | 13.385882E |
|  | 7 | Gunnesbo | Lund | Skåne | 55.734731N | 13.153120E |
|  | 8 | Habo Gard | Lomma | Skåne | 55.692279N | 13.074879E |
|  | 9 | Holmeja | Svedala | Skåne | 55.545279N | 13.281841E |
|  | 10 | Höje Å 6 | Lund | Skåne | 55.669539N | 13.300263E |
|  | 11 | Höje Å 7 | Lund | Skåne | 55.678972N | 13.265889E |
|  | 12 | Höje Å 14 | Lund | Skåne | 55.649652N | 13.319915E |
|  | 13 | IKEA | Malmö | Skåne | 55.553481N | 12.983885E |
|  | 14 | Ilstorp | Sjöbo | Skåne | 55.616557N | 13.664537E |
|  | 15 | Krutladan | Lund | Skåne | 55.650422N | 13.446847E |
|  | 16 | Lunnarp | Lund | Skåne | 55.649733N | 13.332357E |
|  | 17 | Råbydammen | Lund | Skåne | 55.686339N | 13.233592E |
|  | 18 | Vombs Bruksgård | Sjöbo | Skåne | 55.653528N | 13.600694E |
|  | 19 | Vombs Vattenwerk | Lund | Skåne | 55.657952N | 13.542964E |
|  |  |  |  |  |  |  |
| **Targeted catching** | 1 | Aletäppet Ripa | Kristianstad | Skåne | 55.925030N | 14.214790E |
|  | 2 | Attarp | Lund | Skåne | 55.617074N | 13.366822E |
|  | 3 | Eksholmssjon | Svedala | Skåne | 55.553572N | 13.313910E |
|  | 4 | Hultseröd | Eslöv | Skåne | 55.968451N | 13.352225E |
|  | 5 | Kulladals karr | Tomellila | Skåne | 55.650000N | 14.018361E |
|  | 6 | Långasjönäs Kroksjöarna | Karlshamn | Blekinge | 56.254070N | 14.857493E |
|  | 7 | Listarums grustag | Tommellila | Skåne | 55.587725N | 14.156070E |
|  | 8 | Mörrumsån Rosendala | Karlshamn | Blekinge | 56.215530N | 14.755258E |
|  | 9 | Ny Våtmark Köppinge | Kristianstad | Skåne | 55.936869N | 14.202740E |
|  | 10 | Pydden | Svedala | Skåne | 55.552234N | 13.299557E |
|  | 11 | Sövdeborgs slott | Sjöbo | Skåne | 55.581139N | 13.695389E |
|  | 12 | Sövdemolla | Sjöbo | Skåne | 55.601556N | 13.656849E |
|  | 13 | Stensoffa Field Station | Lund | Skåne | 55.695366N | 13.447837E |
|  | 14 | Rönne å Gunnaröd | Eslöv | Skåne | 55.949450N | 13.351280E |
|  | 15 | Ladugårdsmarken | Lund | Skåne | 55.739416N | 13.209258E |

Localities for community sampling were visited regularly throughout the summer seasons of 2021 and 2022, whereas the localities for targeted catching were only visited 1–2 times, aiming for rare species. The distribution of sampling locations is shown in Supplementary Fig. S5.

**Supplementary Table S10:** Overview of the 63 Odonata species used to study the metabolic allometry

| **Species** | **Family** | **Suborder** | **Behavior** | **Sample size** | **SMR** | **Body mass** | **Incubation time** | **Flow rate** |
| --- | --- | --- | --- | --- | --- | --- | --- | --- |
| *Aeshna affinis* | Aeshnidae | Anisoptera | Flier | 5 | 116.86 | 195.38 | 25.00 | 474.4 |
| *Aeshna cyanea* | Aeshnidae | Anisoptera | Flier | 14 | 130.87 | 274.18 | 27.73 | 476.0 |
| *Aeshna grandis* | Aeshnidae | Anisoptera | Flier | 21 | 152.39 | 308.44 | 26.05 | 480.7 |
| *Aeshna isoceles* | Aeshnidae | Anisoptera | Flier | 1 | 138.88 | 256.10 | 31.00 | 494.3 |
| *Aeshna mixta* | Aeshnidae | Anisoptera | Flier | 17 | 81.50 | 157.74 | 32.47 | 465.3 |
| *Aeshna subarctica* | Aeshnidae | Anisoptera | Flier | 3 | 112.81 | 273.90 | 42.67 | 479.1 |
| *Aeshna viridis* | Aeshnidae | Anisoptera | Flier | 2 | 146.86 | 308.35 | 33.00 | 471.4 |
| *Anax imperator* | Aeshnidae | Anisoptera | Flier | 14 | 170.55 | 354.17 | 21.80 | 491.5 |
| *Anax junius* | Aeshnidae | Anisoptera | Flier | 7 | 136.93 | 361.92 | –– | –– |
| *Anax parthenope* | Aeshnidae | Anisoptera | Flier | 2 | 94.27 | 251.55 | 29.00 | 494.1 |
| *Archilestes grandis* | Lestidae | Zygoptera | Percher | 8 | 27.60 | 62.95 | 18.00 | 100.0 |
| *Brachymesia gravida* | Libellulidae | Anisoptera | Percher | 8 | 51.35 | 122.18 | –– | –– |
| *Brachytron pratense* | Aeshnidae | Anisoptera | Flier | 4 | 72.48 | 180.35 | 46.00 | –– |
| *Calopteryx splendens* | Calopterygidae | Zygoptera | Percher | 14 | 15.29 | 46.17 | 59.29 | 250.5 |
| *Calopteryx virgo* | Calopterygidae | Zygoptera | Percher | 11 | 17.10 | 51.04 | 61.27 | 249.5 |
| *Coenagrion puella* | Coenagrionidae | Zygoptera | Percher | 20 | 3.69 | 11.80 | 65.90 | –– |
| *Coenagrion pulchellum* | Coenagrionidae | Zygoptera | Percher | 14 | 4.48 | 12.79 | 60.21 | 147.3 |
| *Cordulegaster boltonii* | Cordulegastridae | Anisoptera | Flier | 13 | 110.01 | 326.59 | 35.00 | 476.1 |
| *Cordulia aenea* | Corduliidae | Anisoptera | Flier | 10 | 59.12 | 116.27 | 32.80 | 481.5 |
| *Enallagma civile* | Coenagrionidae | Zygoptera | Percher | 8 | 10.88 | 10.74 | 18.00 | 100.0 |
| *Enallagma cyathigerum* | Coenagrionidae | Zygoptera | Percher | 26 | 5.04 | 14.95 | 59.62 | 179.3 |
| *Erythemis simplicicollis* | Libellulidae | Anisoptera | Percher | 14 | 31.45 | 90.11 | –– | –– |
| *Erythrodiplax berenice* | Libellulidae | Anisoptera | Percher | 8 | 14.48 | 44.40 | –– | –– |
| *Erythrodiplax connata* | Libellulidae | Anisoptera | Percher | 14 | 9.09 | 17.40 | –– | –– |
| *Erythromma najas* | Coenagrionidae | Zygoptera | Percher | 11 | 8.04 | 23.16 | 59.27 | 145.3 |
| *Erythromma viridulum* | Coenagrionidae | Zygoptera | Percher | 20 | 4.85 | 15.20 | 61.15 | 235.9 |
| *Gomphus vulgatissimus* | Gomphidae | Anisoptera | Percher | 7 | 37.42 | 131.21 | 33.14 | 488.3 |
| *Ischnura elegans* | Coenagrionidae | Zygoptera | Percher | 15 | 5.23 | 10.51 | 78.13 | –– |
| *Ischnura hastata* | Coenagrionidae | Zygoptera | Percher | 1 | 2.33 | 3.59 | 46.00 | 100.0 |
| *Ischnura posita* | Coenagrionidae | Zygoptera | Percher | 23 | 2.72 | 3.78 | 24.10 | 100.0 |
| *Ischnura pumilio* | Coenagrionidae | Zygoptera | Percher | 13 | 5.03 | 10.85 | 62.62 | 188.6 |
| *Ischnura verticalis* | Coenagrionidae | Zygoptera | Percher | 18 | 5.37 | 7.18 | 26.20 | 100.0 |
| *Lestes dryas* | Lestidae | Zygoptera | Percher | 28 | 6.89 | 20.63 | 59.54 | 169.9 |
| *Lestes sponsa* | Lestidae | Zygoptera | Percher | 20 | 7.70 | 21.35 | 67.25 | 174.1 |
| *Lestes virens* | Lestidae | Zygoptera | Percher | 21 | 3.59 | 10.41 | 59.24 | 188.9 |
| *Leucorrhinia dubia* | Libellulidae | Anisoptera | Percher | 10 | 24.91 | 50.61 | 29.91 | 417.1 |
| *Leucorrhinia pectoralis* | Libellulidae | Anisoptera | Flier | 3 | 33.46 | 85.73 | 32.00 | –– |
| *Libellula auripennis* | Libellulidae | Anisoptera | Percher | 8 | 46.08 | 164.80 | –– | –– |
| *Libellula depressa* | Libellulidae | Anisoptera | Flier | 7 | 81.74 | 198.83 | 30.75 | 484.8 |
| *Libellula fulva* | Libellulidae | Anisoptera | Percher | 7 | 46.14 | 157.76 | 34.43 | 380.5 |
| *Libellula needhami* | Libellulidae | Anisoptera | Percher | 8 | 55.30 | 183.98 | –– | –– |
| *Libellula quadrimaculata* | Libellulidae | Anisoptera | Percher | 9 | 52.04 | 142.68 | 29.56 | 488.7 |
| *Libellula* sp. | Libellulidae | Anisoptera | Flier | 7 | 51.35 | 174.39 | –– | –– |
| *Miathyria marcella* | Libellulidae | Anisoptera | Flier | 12 | 36.87 | 65.35 | –– | –– |
| *Onychogomphus forcipatus* | Gomphidae | Anisoptera | Percher | 15 | 40.89 | 99.55 | 35.40 | 466.2 |
| *Orthetrum cancellatum* | Libellulidae | Anisoptera | Percher | 12 | 61.93 | 159.08 | 30.33 | 485.8 |
| *Orthetrum coerulescens* | Libellulidae | Anisoptera | Percher | 21 | 42.30 | 83.21 | 35.86 | 471.0 |
| *Pachydiplax longipennis* | Libellulidae | Anisoptera | Percher | 29 | 26.89 | 66.73 | –– | –– |
| *Pantala flavescens* | Libellulidae | Anisoptera | Flier | 8 | 56.38 | 114.32 | –– | –– |
| *Perithemis tenera* | Libellulidae | Anisoptera | Percher | 9 | 10.93 | 21.67 | –– | –– |
| *Platycnemis pennipes* | Platycnemididae | Zygoptera | Percher | 16 | 5.69 | 15.73 | 62.50 | 303.7 |
| *Pyrrhosoma nymphula* | Coenagrionidae | Zygoptera | Percher | 5 | 3.97 | 18.20 | 61.20 | –– |
| *Somatochlora flavomaculata* | Corduliidae | Anisoptera | Flier | 6 | 59.09 | 124.07 | 31.83 | 485.4 |
| *Somatochlora metallica* | Corduliidae | Anisoptera | Flier | 7 | 79.55 | 160.27 | 29.38 | 482.2 |
| *Sympecma fusca* | Lestidae | Zygoptera | Percher | 2 | 3.82 | 12.15 | 64.00 | 211.2 |
| *Sympetrum ambiguum* | Libellulidae | Anisoptera | Percher | 8 | 30.01 | 41.23 | 15.10 | 100.0 |
| *Sympetrum danae* | Libellulidae | Anisoptera | Percher | 18 | 13.06 | 32.83 | 32.50 | 441.0 |
| *Sympetrum flaveolum* | Libellulidae | Anisoptera | Percher | 7 | 17.90 | 34.33 | 37.57 | 304.9 |
| *Sympetrum sanguineum* | Libellulidae | Anisoptera | Percher | 30 | 20.71 | 44.77 | 31.10 | 448.8 |
| *Sympetrum striolatum* | Libellulidae | Anisoptera | Percher | 15 | 27.40 | 66.51 | 31.80 | 461.2 |
| *Sympetrum vicinum* | Libellulidae | Anisoptera | Percher | 1 | 21.32 | 42.61 | 10.00 | 100.0 |
| *Sympetrum vulgatum* | Libellulidae | Anisoptera | Percher | 17 | 24.99 | 69.48 | 36.65 | 465.4 |
| *Tramea carolina* | Libellulidae | Anisoptera | Flier | 10 | 59.25 | 136.03 | –– | –– |

**Supplementary Table S11:** Seven of the 63 Odonata species did not occur in the phylogeny and were therefore replaced by the most closely related species of the same genus using recent phylogenies or field guides

| **Original taxa** | **Replaced taxa** | **Explanation** |
| --- | --- | --- |
| *Ischnura elegans* | *I. selegalensis* | Most closely related species according to the molecular phylogeny of Blow et al. (2021) |
| *Aeshna isosceles* | *A. juncea* | European species of the genus according to Dijkstra et al. (2020) (no phylogeny of this genus is available) |
| *Erythrodiplax berenice* | *E. unimaculata* | Closely related species of the same “*Unimaculata*” species group, according to the morphological revision of this genus (Borror 1942) |
| *Erythrodiplax conntata* | *E. fusca* | Closely related species of the same “*Connata*” species group, according to the morphological revision of this genus (Borror 1942) |
| *Tramea carolina* | *T. lacerta* | Choosing a random species in the same genus (Paulson 2011) (no complete phylogeny available for this genus) |
| *Orthetrum cancellatum* | *O. brunneum* | European species of the same genus which is morphologically quite similar (Dijkstra *et al.* 2020) (no phylogeny of this genus, that includes *O. cancellatum*, is available) |
| *Libellula*sp. | *L. flavida* | Choosing a random species of the same genus which occurs in Florida, USA (where May 1979a collected his *Libellula* sp. specimens) |

The choice of the replacement taxa did not affect the interpretation of our main results (Supplementary Table S12).**Table S12:** Evaluation of how replacement taxa could affect the robustness of the main results

| **Model or test** | **N** | **Parameter** | **Estimate (± SE)** |
| --- | --- | --- | --- |
| **PGLS_OUfixedRoot_: SMR on BM** | 10^–4^ | Slope mean | 0.87 ± 1.1×10^–4^ |
|  |  | Slope lower CI | 0.77 ± 1.1×10^–4^ |
|  |  | Slope upper CI | 0.97 ± 1.1×10^–4^ |
|  |  | Intercept mean | –4.20 ± 1.8×10^–4^ |
|  |  |  |  |
| **PGLS_δ_: SMR on BM, using fresh instead of** | 10^–4^ | Slope mean | 0.90 ± 1.7×10^–4^ |
| **dry mass** |  | Slope lower CI | 0.81 ± 2.0×10^–4^ |
|  |  | Slope upper CI | 0.99 ± 1.4×10^–4^ |
|  |  | Intercept mean | –4.64 ± 3.5×10^–4^ |
|  |  |  |  |
| **PGLS_κ_: SMR on BM, with sample size ≤ 10** | 10^–4^ | Slope mean | 0.87 ± 2.2×10^–6^ |
|  |  | Slope lower CI | 0.77 ± 4.4×10^–6^ |
|  |  | Slope upper CI | 0.96 ± 5.8×10^–7^ |
|  |  | Intercept mean | –4.21 ± 3.9×10^–6^ |
|  |  |  |  |
| **Evidence for curvilinear metabolic allometry** | 10^–4^ | Quadratic coefficient mean | 0.11 ± 8.9×10^–5^ |
| **(quadratic PGLS)** |  | Quadratic coefficient lower CI | –0.0037 ± 7.4×10^–5^ |
|  |  | Quadratic coefficient upper CI | 0.065 ± 1.9×10^–4^ |
|  |  | Quadratic coefficient p‑value | 0.22 ± 1.1×10^–4^ |
|  |  | ΔAICc (PGLS_quadratic_ – PGLS) | –0.75 ± 1.0×10^–2^ |
|  |  |  |  |
| **Evidence for differences in metabolic** | 10^–4^ | p‑value for different intercept | 0.63 ± 5.5×10^–4^ |
| **allometry between suborders (pANCOVA)** |  | p‑value for different slope | 0.69 ± 1.0×10^–3^ |
|  |  |  |  |
| **Evidence for differences in metabolic** | 10^–4^ | p‑value for different intercept | 0.47 ± 8.4×10^–4^ |
| **allometry between flight behaviors (pANCOVA)** |  | pvalue for different slope | 0.35 ± 7.9×10^–4^ |
|  |  |  |  |
| **Evidence for a multi-optima OU behavior** | 155 | ΔAICc (_mo_OU behavior – other models) | 1.46 ± 5.8×10^–2^ |
| **model for standard metabolic rate** |  | Akaike weight | 0.50 ± 4.9×10^–3^ |
|  |  |  |  |
| **Evidence for a multi-optima OU behavior** | 155 | ΔAICc (_mo_OU behavior – other models) | 0.11 ± 2.7×10^–2^ |
| **model for mass-independent SMR** |  | Akaike weight | 0.26 ± 3.8×10^–3^ |

To evaluate the robustness of our results to the choice of the seven replacement taxa (Supplementary Table S11), we re-ran the main analyses N times and randomly picked a species out of the respective genus available in the phylogeny as replacement taxa for each run. For computational reasons and model complexity, only maximum-likelihood models and no reversible-jumb Markov chain Monte Carlo modes were rerun. AICc = sample-size corrected Akaike Information Criterion, CI = 95% confidence interval, mo = multi-optima, OU = Ornstein-Uhlenbeck, pANCOVA = phylogenetic analysis of covariance, PGLS = phylogenetic generalized least squares, SE = standard error, SMR = standard metabolic rate.

**Supplementary Table S13:** Overview of the 20 Odonata species used to estimate the cell and nucleus sizes (area in µm^2^), including their respective family, suborder, flight behavior and sample size (number of individuals)

| **Species** | **Family** | **Suborder** | **Behavior** | **Sample size** | **Cell area** | **Nucleus area** |
| --- | --- | --- | --- | --- | --- | --- |
| *Aeshna cyanea* | Aeshnidae | Anisoptera | Flier | 1 | 124.27 | 36.60 |
| *Aeshna isoceles* | Aeshnidae | Anisoptera | Flier | 2 | 173.42 | 53.68 |
| *Aeshna mixta* | Aeshnidae | Anisoptera | Flier | 2 | 164.62 | 50.15 |
| *Anax imperator* | Aeshnidae | Anisoptera | Flier | 1 | 148.05 | 39.86 |
| *Brachytron pratense* | Aeshnidae | Anisoptera | Flier | 2 | 159.31 | 57.77 |
| *Calopteryx splendens* | Calopterygidae | Zygoptera | Percher | 1 | 95.22 | 33.86 |
| *Coenagrion hastulatum* | Coenagrionidae | Zygoptera | Percher | 1 | 139.72 | 49.86 |
| *Cordulegaster boltonii* | Cordulegastridae | Anisoptera | Flier | 1 | 188.89 | 57.45 |
| *Ischnura elegans* | Coenagrionidae | Zygoptera | Percher | 2 | 151.68 | 46.79 |
| *Ischnura pumilio* | Coenagrionidae | Zygoptera | Percher | 2 | 112.13 | 39.56 |
| *Lestes sponsa* | Lestidae | Zygoptera | Percher | 1 | 143.32 | 46.06 |
| *Lestes virens* | Lestidae | Zygoptera | Percher | 1 | 88.05 | 29.67 |
| *Leucorrhinia dubia* | Libellulidae | Anisoptera | Percher | 2 | 119.33 | 40.55 |
| *Libellula fulva* | Libellulidae | Anisoptera | Percher | 1 | 126.13 | 43.70 |
| *Onychogomphus forcipatus* | Gomphidae | Anisoptera | Percher | 2 | 132.35 | 42.37 |
| *Orthetrum cancellatum* | Libellulidae | Anisoptera | Percher | 1 | 116.92 | 42.79 |
| *Orthetrum coerulescens* | Libellulidae | Anisoptera | Percher | 2 | 118.11 | 39.49 |
| *Pyrrhosoma nymphula* | Coenagrionidae | Zygoptera | Percher | 1 | 115.99 | 42.44 |
| *Sympetrum sanguineum* | Libellulidae | Anisoptera | Percher | 1 | 158.85 | 46.40 |
| *Sympetrum striolatum* | Libellulidae | Anisoptera | Percher | 2 | 129.65 | 35.58 |

For cell and nucleus area, species-specific means are shown.

**Supplementary Table S14:** Overview of the 47 Odonata species used to estimate the body volume (mm^3^) and body surface area (mm^2^), including their respective family, suborder, flight behavior and sample size (number of individuals)

| **Species** | **Family** | **Suborder** | **Behavior** | **Sample size** | **Volume** | **Surface area** |
| --- | --- | --- | --- | --- | --- | --- |
| *Aeshna affinis* | Aeshnidae | Anisoptera | Flier | 1 | 716.60 | 73.97 |
| *Aeshna cyanea* | Aeshnidae | Anisoptera | Flier | 1 | 1612.80 | 128.32 |
| *Aeshna grandis* | Aeshnidae | Anisoptera | Flier | 2 | 1313.88 | 114.49 |
| *Aeshna isoceles* | Aeshnidae | Anisoptera | Flier | 3 | 1192.02 | 104.57 |
| *Aeshna mixta* | Aeshnidae | Anisoptera | Flier | 2 | 788.25 | 79.99 |
| *Aeshna subarctica* | Aeshnidae | Anisoptera | Flier | 1 | 1086.20 | 103.47 |
| *Aeshna viridis* | Aeshnidae | Anisoptera | Flier | 1 | 1231.00 | 104.98 |
| *Anax imperator* | Aeshnidae | Anisoptera | Flier | 2 | 1634.17 | 131.60 |
| *Anax parthenope* | Aeshnidae | Anisoptera | Flier | 1 | 1380.50 | 116.08 |
| *Brachytron pratense* | Aeshnidae | Anisoptera | Flier | 2 | 924.82 | 88.79 |
| *Calopteryx splendens* | Calopterygidae | Zygoptera | Percher | 2 | 155.94 | 31.05 |
| *Calopteryx virgo* | Calopterygidae | Zygoptera | Percher | 2 | 193.07 | 36.43 |
| *Coenagrion hastulatum* | Coenagrionidae | Zygoptera | Percher | 1 | 32.90 | 11.86 |
| *Coenagrion lunulatum* | Coenagrionidae | Zygoptera | Percher | 1 | 41.40 | 13.24 |
| *Coenagrion puella* | Coenagrionidae | Zygoptera | Percher | 2 | 38.37 | 13.28 |
| *Coenagrion pulchellum* | Coenagrionidae | Zygoptera | Percher | 2 | 36.15 | 12.74 |
| *Cordulegaster boltonii* | Cordulegastridae | Anisoptera | Flier | 2 | 1451.18 | 123.37 |
| *Cordulia aenea* | Corduliidae | Anisoptera | Flier | 3 | 515.51 | 61.42 |
| *Enallagma cyathigerum* | Coenagrionidae | Zygoptera | Percher | 5 | 44.73 | 13.88 |
| *Epitheca bimaculata* | Corduliidae | Anisoptera | Flier | 1 | 1006.60 | 96.56 |
| *Erythromma najas* | Coenagrionidae | Zygoptera | Percher | 2 | 74.98 | 18.95 |
| *Erythromma viridulum* | Coenagrionidae | Zygoptera | Percher | 2 | 44.65 | 13.77 |
| *Gomphus vulgatissimus* | Gomphidae | Anisoptera | Percher | 2 | 618.36 | 67.47 |
| *Ischnura elegans* | Coenagrionidae | Zygoptera | Percher | 3 | 40.04 | 13.21 |
| *Ischnura pumilio* | Coenagrionidae | Zygoptera | Percher | 2 | 34.96 | 11.93 |
| *Lestes dryas* | Lestidae | Zygoptera | Percher | 1 | 68.90 | 18.34 |
| *Lestes sponsa* | Lestidae | Zygoptera | Percher | 2 | 55.99 | 16.31 |
| *Lestes virens* | Lestidae | Zygoptera | Percher | 3 | 43.99 | 14.46 |
| *Leucorrhinia dubia* | Libellulidae | Anisoptera | Percher | 1 | 210.60 | 33.24 |
| *Leucorrhinia pectoralis* | Libellulidae | Anisoptera | Flier | 1 | 419.20 | 51.60 |
| *Leucorrhinia rubicunda* | Libellulidae | Anisoptera | Percher | 2 | 289.11 | 40.68 |
| *Libellula depressa* | Libellulidae | Anisoptera | Flier | 3 | 918.83 | 79.87 |
| *Libellula fulva* | Libellulidae | Anisoptera | Percher | 1 | 683.30 | 67.37 |
| *Libellula quadrimaculata* | Libellulidae | Anisoptera | Percher | 2 | 760.43 | 74.08 |
| *Onychogomphus forcipatus* | Gomphidae | Anisoptera | Percher | 1 | 361.80 | 48.81 |
| *Orthetrum cancellatum* | Libellulidae | Anisoptera | Percher | 3 | 626.94 | 66.85 |
| *Orthetrum coerulescens* | Libellulidae | Anisoptera | Percher | 1 | 316.00 | 42.96 |
| *Platycnemis pennipes* | Platycnemididae | Zygoptera | Percher | 2 | 53.33 | 16.21 |
| *Pyrrhosoma nymphula* | Coenagrionidae | Zygoptera | Percher | 3 | 72.26 | 18.73 |
| *Somatochlora flavomaculata* | Corduliidae | Anisoptera | Flier | 1 | 474.30 | 59.93 |
| *Somatochlora metallica* | Corduliidae | Anisoptera | Flier | 2 | 669.38 | 74.58 |
| *Sympecma fusca* | Lestidae | Zygoptera | Percher | 1 | 54.10 | 16.06 |
| *Sympetrum danae* | Libellulidae | Anisoptera | Percher | 2 | 143.12 | 25.64 |
| *Sympetrum flaveolum* | Libellulidae | Anisoptera | Percher | 1 | 167.20 | 28.22 |
| *Sympetrum sanguineum* | Libellulidae | Anisoptera | Percher | 2 | 192.35 | 30.23 |
| *Sympetrum striolatum* | Libellulidae | Anisoptera | Percher | 1 | 266.50 | 38.12 |
| *Sympetrum vulgatum* | Libellulidae | Anisoptera | Percher | 3 | 258.74 | 37.25 |

For body volume and body surface area, species-specific means are shown.

Supplementary Table S15: Output from regression models assuming different respiratory quotients for data conversion

| **RQ** | **AICc** | **ΔAICc** | **ω** | **R^2^** | **Residual SE** | **Intercept** | **Slope** |
| --- | --- | --- | --- | --- | --- | --- | --- |
| **0.79** | –85.55 | –– | 0.38 | 0.95 | 0.119 | –4.325 ± 0.052 | 0.962 ± 0.028 |
| **0.80** | –85.47 | 0.08 | 0.36 | 0.95 | 0.119 | –4.325 ± 0.052 | 0.962 ± 0.028 |
| **0.84** | –84.79 | 0.76 | 0.26 | 0.95 | 0.120 | –4.326 ± 0.052 | 0.965 ± 0.028 |
| **1.00** | –78.11 | 7.44 | 0.01 | 0.95 | 0.126 | –4.237 ± 0.055 | 0.974 ± 0.030 |

After converting the oxygen consumption rate (VO_2_) data of May (1979) to carbon dioxide production rate (VCO_2_) assuming different respiratory quotients (RQ), we combined these data with our metabolic allometry data. We used linear ordinary least squares regression with log_10_ standard metabolic rate (ml CO_2_/min) as response and log_10_ body mass (mg) as predictor variable to estimate the interspecific metabolic allometry for 63 Odonata species. The best‑fitting model was chosen by evaluating the differences in corrected Akaike Information Criterion (AICc) scores, Akaike weight ω, R^2^ (coefficient of determination) and residual standard errors (SE). Estimates are given with SE.

**Supplementary References**

Abbott, J.C. (2015). *Dragonflies of Texas*. University of Texas Press, New York.

Addo-Bediako, A., Chown, S.L. & Gaston, K.J. (2002). Metabolic cold adaptation in insects: A large-scale perspective. *Funct Ecol*, 16, 332–338.

Akaike, H. (1974). A new look at the statistical model identification. *IEEE Trans Automat Contr*, 19, 716–723.

Auguie, B. (2017). gridExtra: Miscellaneous functions for “grid” graphics.

Bates, D., Mächler, M., Bolker, B. & Walker, S. (2015). Fitting linear mixed-effects models using lme4. *J Stat Softw*, 67, 1–48.

Beaulieu, J.M., Jhwueng, D.C., Boettiger, C. & O’Meara, B.C. (2012). Modeling stabilizing selection: Expanding the Ornstein-Uhlenbeck model of adaptive evolution. *Evolution (N Y)*, 66, 2369–2383.

Beaulieu, J.M. & O’Meara, B.C. (2022). OUwie: Analysis of evolutionary rates in an OU framework.

van der Bijl, W. (2018). phylopath: Easy phylogenetic path analysis in R. *PeerJ*, 2018, e4718.

Blomberg, S.P., Garland, T. & Ives, A.R. (2003). Testing for phylogenetic signal in comparative data: Behavioral traits are more labile. *Evolution (N Y)*, 57, 717–745.

Blow, R., Willink, B. & Svensson, E.I. (2021). A molecular phylogeny of forktail damselflies (genus Ischnura) reveals a dynamic macroevolutionary history of female colour polymorphisms. *Mol Phylogenet Evol*, 160, 107134.

Bollback, J.P. (2006). SIMMAP: Stochastic character mapping of discrete traits on phylogenies. *BMC Bioinformatics*, 7, 88.

Borror, D.J. (1942). A revision of the libelluline genus Erythrodiplax (Odonata). *Contributions in Zoology and Entomology*, 4, 1–286.

Brooks, S.P. & Gelman, A. (1998). General methods for monitoring convergence of iterative simulations. *Journal of Computational and Graphical Statistics*, 7, 434–455.

Burnham, K.P. & Anderson, D.R. (2003). *Model selection and multimodel inference: A practical information theoretic approach*. Springer Science & Business Media, New York.

Butler, M.A. & King, A.A. (2004). Phylogenetic comparative analysis. A modeling approach for adaptive evolution. *Am Nat*, 164, 683–695.

Capellini, I., Venditti, C. & Barton, R.A. (2010). Phylogeny and metabolic scaling in mammals. *Ecology*, 91, 2783–2793.

Cavanaugh, J.E. (1997). Unifying the derivations for the Akaike and corrected Akaike information criteria. *Stat Probab Lett*, 33, 201–208.

Chesher, A. (1991). The effect of measurement error. *Biometrika*, 78, 451–462.

Chown, S.L., Marais, E., Terblanche, J.S., Klok, C.J., Lighton, J.R.B. & Blackburn, T.M. (2007). Scaling of insect metabolic rate is inconsistent with the nutrient supply network model. *Funct Ecol*, 21, 282–290.

Community, B.O. (2018). Blender - A 3D modelling and rendering package.

Cooper, N., Thomas, G.H., Venditti, C., Meade, A. & Freckleton, R.P. (2016). A cautionary note on the use of Ornstein Uhlenbeck models in macroevolutionary studies. *Biological Journal of the Linnean Society*, 118, 64–77.

Corbet, P.S. (1962). *A biology of dragonflies*. *Entomology research institute*. H. F. & G. Witherby LTD, London.

Corbet, P.S. (1999). *Dragonflies - Behaviour and ecology of Odonata*. Harley Books, Colchester.

Corbet, P.S. & May, M.L. (2008). Fliers and perchers among Odonata: Dichotomy or multidimensional continuum? A provisional reappraisal. *International Journal of Odonatology*, 11, 155–171.

Cuff, A.R., Randau, M., Head, J., Hutchinson, J.R., Pierce, S.E. & Goswami, A. (2015). Big cat, small cat: Reconstructing body size evolution in living and extinct Felidae. *J Evol Biol*, 28, 1516–1525.

Dijkstra, K.D.B., Schröter, A. & Lewington, R. (2020). *Field guide to the dragonflies of Britain and Europe*. Second Edi. Bloomsbury Publishing, London.

Dodds, P.S., Rothman, D.H. & Weitz, J.S. (2001). Re-examination of the “3/4-law” of metabolism. *J Theor Biol*, 209, 9–27.

Duncan, F.D. & Crewe, R.M. (1993). A comparison of the energetics of foraging of three species of Leptogenys (Hymenoptera, Formicidae). *Physiol Entomol*, 18, 372–378.

Felsenstein, J. (1973). Maximum likelihood estimation of evolutionary trees from continuous characters. *Am J Hum Genet*, 25, 471–492.

Felsenstein, J. (1985). Phylogenies and the comparative method. *Am Nat*, 125, 1–15.

Felsenstein, J. (2008). Comparative methods with sampling error and within-species variation: Contrasts revisited and revised. *Am Nat*, 171, 713–725.

Fitzjohn, R.G., Maddison, W.P. & Otto, S.P. (2009). Estimating trait-dependent speciation and extinction rates from incompletely resolved phylogenies. *Syst Biol*, 58, 595–611.

Freckleton, R.P., Harvey, P.H. & Pagel, M.D. (2002). Phylogenetic analysis and comparative data: A test and review of evidence. *Am Nat*, 160, 712–726.

Garamszegi, L.Z. (2014a). *Modern phylogenetic comparative methods and their application in evolutionary biology. Concepts and practice*. Springer, Heidelberg, New York, Dordrecht, London.

Garamszegi, L.Z. (2014b). Uncertainties due to within-species variation in comparative studies: Measurement errors and statistical weights. In: *Modern phylogenetic comparative methods and their application in evolutionary biology. Concepts and Practice* (ed. Garamszegi, L.Z.). Springer, Berlin and Heidelberg, pp. 157–200.

Garamszegi, L.Z. & Møller, A.P. (2010). Effects of sample size and intraspecific variation in phylogenetic comparative studies: A meta-analytic review. *Biological Reviews*, 85, 797–805.

Garland, T., Bennett, A.F. & Rezende, E.L. (2005). Phylogenetic approaches in comparative physiology. *Journal of Experimental Biology*, 208, 3015–3035.

Garland, T. & Ives, A.R. (2000). Using the past to predict the present: Confidence intervals for regression equations in phylogenetic comparative methods. *Am Nat*, 155, 346–364.

Garnier, S., Ross, N., Rudis, B., Sciaini, M., Camargo, A.P. & Scherer, C. (2023). viridis(Lite) - Colorblind-friendly color maps for R.

Geisser, S. (1975). The predictive sample reuse method with applications. *J Am Stat Assoc*, 70, 320–328.

Gelman, A. & Rubin, D.B. (1992). Inference from iterative simulation using multiple sequences. *Statistical Science*, 7, 457–472.

Gonzalez-Voyer, A. & von Hardenberg, A. (2014). An introduction to phylogenetic path analysis. In: *Modern phylogenetic comparative methods and their application in evolutionary biology. Concepts and Practice* (ed. Garamszegi, L.Z.). Springer-Verlag, Berlin and Heidelberg, pp. 201–230.

Grabowski, M., Kopperud, B.T., Tsuboi, M. & Hansen, T.F. (2023). Both diet and sociality affect primate brain-size evolution. *Syst Biol*, 72, 404–418.

Grafen, A. (1989). The phylogenetic regression. *Philosophical Transactions of the Royal Society B: Biological Sciences*, 326, 119–157.

Green, P.J. (1995). Reversible jump Markov chain Monte Carlo computation and Bayesian model determination. *Biometrika*, 82, 711–732.

Hansen, T.F. (1997). Stabilizing selection and the comparative analysis of adaptation. *Evolution (N Y)*, 51, 1341–1351.

Hansen, T.F., Pienaar, J. & Orzack, S.H. (2008). A comparative method for studying adaptation to a randomly evolving environment. *Evolution (N Y)*, 62, 1965–1977.

von Hardenberg, A. & Gonzalez-Voyer, A. (2012). Disentangling evolutionary cause-effect relationships with phylogenetic confirmatory path analysis. *Evolution (N Y)*, 67, 378–387.

Harmon, L. (2023). treeplyr: “dplyr” functionality for matched tree and data objects.

Harmon, L.J. (2019). *Phylogenetic comparative methods*. *Current Biology*.

Harmon, L.J. & Losos, J.B. (2005). The effect of intraspecific sample size on type I and type II error rates in comparative studies. *Evolution (N Y)*, 59, 2705.

Harmon, L.J., Losos, J.B., Jonathan Davies, T., Gillespie, R.G., Gittleman, J.L., Bryan Jennings, W., *et al.* (2010). Early bursts of body size and shape evolution are rare in comparative data. *Evolution (N Y)*, 2385–2396.

Harvey, P.H. & Pagel, M.D. (1991). *The comparative method in evolutionary biology*. Oxford University Press, Oxford, New York & Tokyo.

Ho, L.S.T. & Ane, C. (2014). A linear-time algorithm for Gaussian and non-Gaussian trait evolution models. *Syst Biol*, 63, 397–408.

Huelsenbeck, J.P., Nielsen, R. & Bollback, J.P. (2003). Stochastic mapping of morphological characters. *Syst Biol*, 52, 131–158.

Ives, A.R., Midford, P.E. & Garland, T. (2007). Within-species variation and measurement error in phylogenetic comparative methods. *Syst Biol*, 56, 252–270.

Jasienski, M. & Bazzaz, F.A. (1999). The fallacy of ratios and the testability of models in biology. *Oikos*, 84, 321.

Keklikoglou, K., Arvanitidis, C., Chatzigeorgiou, G., Chatzinikolaou, E., Karagiannidis, E., Koletsa, T., *et al.* (2021). Micro‐ct for biological and biomedical studies: A comparison of imaging techniques. *J Imaging*, 7.

Kleiber, M. (1932). Body size and metabolism. *Hilgardia*, 6, 315–353.

Kleiber, M. (1961). *The fire of life. An introduction to animal energetics*. John Wiley & Sons, New York & London.

Kolokotrones, T., Van Savage, Deeds, E.J. & Fontana, W. (2010). Curvature in metabolic scaling. *Nature*, 464, 753–756.

Kratochvíl, L. & Rovatsos, M. (2021). Ratios can be misleading for detecting selection. *Current Biology*, 32, R28–R30.

Li, H., Zhang, H., Tang, Z. & Hu, G. (2008). Micro-computed tomography for small animal imaging: Technological details. *Progress in Natural Science*, 18, 513–521.

Lighton, J.R.B. (2008). *Measuring metabolic rates. A manual for scientists*. Oxford University Press, New York.

Lighton, J.R.B. & Halsey, L.G. (2011). Flow-through respirometry applied to chamber systems: Pros and cons, hints and tips. *Comparative Biochemistry and Physiology - A Molecular and Integrative Physiology*, 158, 265–275.

Manisha, S. (2001). An estimation of population mean in the presence of measurement error. *Journal of the Indian Society of Agricultural Statistics*, 54, 173–205.

Martins, E.P. & Hansen, T.F. (1997). Phylogenies and the comparative method: A general approach to incorporating phylogenetic information into the analysis of interspecific data. *Am Nat*, 149, 646–667.

May, M.L. (1979a). Energy metabolism of dragonflies (Odonata: Anisoptera) at rest and during endothermic warm-up. *Journal of Experimental Biology*, 83, 79–94.

May, M.L. (1979b). Energy metabolism of dragonflies (Odonata: Anisoptera) at rest and during endothermic warm-up. *J Exp Biol*, 83, 79–94.

Mizutani, R. & Suzuki, Y. (2012). X-ray microtomography in biology. *Micron*, 43, 104–115.

Nakagawa, S. & Schielzeth, H. (2010). Repeatability for Gaussian and non-Gaussian data: A practical guide for biologists. *Biological Reviews*, 85, 935–956.

Nielsen, R. (2002). Mapping mutations on phylogenies. *Syst Biol*, 51, 729–739.

O’Connor, M.P., Agosta, S.J., Hansen, F., Kemp, S.J., Sieg, A.E., McNair, J.N., *et al.* (2007). Phylogeny, regression, and the allometry of physiological traits. *Am Nat*, 170, 431–442.

O’Meara, B.C., Ané, C., Sanderson, M.J. & Wainwright, P.C. (2006). Testing for different rates of continuous trait evolution using likelihood. *Evolution (N Y)*, 60, 922.

O’Meara, B.C. & Beaulieu, J.M. (2014). Modelling stabilizing selection: The attraction of Ohrnstein-Uhlenbeck models. In: *Modern phylogenetic comparative methods and their application in evolutionary biology. Concepts and practice* (ed. Garamszegi, L.Z.). Springer Verlag, Berlin and Heidelberg, pp. 381–393.

Orme, D., Freckleton, R., Thomas, G., Petzoldt, T., Fritz, S., Isaac, N.J.B., *et al.* (2018). caper: Comparative analyses of phylogenetics and evolution in R.

Pagel, M.D. (1997). Inferring evolutionary processes from phylogenies. *Zool Scr*, 26, 331–348.

Pagel, M.D. (1999). Inferring the historical patterns of biological evolution. *Nature*, 401, 877–884.

Paradis, E. & Schliep, K. (2019). ape 5.0: An environment for modern phylogenetics and evolutionary analyses in R. *Bioinformatics*, 35, 526–528.

Paulson, D.R. (2011). *Dragonflies and damselflies of the east*. Princeton University Press, Princeton.

Pennell, M.W., Eastman, J.M., Slater, G.J., Brown, J.W., Uyeda, J.C., FitzJohn, R.G., *et al.* (2014). geiger v2.0: An expanded suite of methods for fitting macroevolutionary models to phylogenetic trees. *Bioinformatics*, 30, 2216–2218.

Pinheiro, J., Bates, D. & R Core Team. (2022). nlme: Linear and nonlinear mixed effects models.

R Core Team. (2021). R: A language and environment for statistical computing.

Revell, L.J. (2009). Size-correction and principal components for interspecific comparative studies. *Evolution (N Y)*, 63, 3258–3268.

Revell, L.J. (2010). Phylogenetic signal and linear regression on species data. *Methods Ecol Evol*, 1, 319–329.

Revell, L.J. (2024). phytools 2.0: An updated R ecosystem for phylogenetic comparative methods (and other things). *PeerJ*, 12, e16505.

Revell, L.J., González-Valenzuela, L.E., Alfonso, A., Castellanos-García, L.A., Guarnizo, C.E. & Crawford, A.J. (2018). Comparing evolutionary rates between trees, clades and traits. *Methods Ecol Evol*, 9, 994–1005.

Revell, L.J. & Harmon, L.J. (2022). *Phylogenetic comparative methods in R*. Princeton University Press, Princeton and Oxford.

Revell, L.J., Harmon, L.J. & Collar, D.C. (2008). Phylogenetic signal, evolutionary process, and rate. *Syst Biol*, 57, 591–601.

Le Rouzic, A., Hansen, T.F., Gosden, T.P. & Svensson, E.I. (2015). Evolutionary time-series analysis reveals the signature of frequency-dependent selection on a female mating polymorphism. *Am Nat*, 185, E182–E196.

RStudio Team. (2022). RStudio.

Rubner, M. (1883). Ueber den Einfluss der Körpergröße auf Stoff und Kraftwechsel. *Z Biol*, 19, 535–562.

Sable Systems International. (2023). *Expedata-P Data Analysis Software*. Available at: https://www.sablesys.com/products/classic-line/expedata-p-data-analysis-software/#product-specs. Last accessed 18 November 2023.

Schambach, S.J., Bag, S., Schilling, L., Groden, C. & Brockmann, M.A. (2010). Application of micro-CT in small animal imaging. *Methods*, 50, 2–13.

Schramm, B.W., Labecka, A.M., Gudowska, A., Antoł, A., Sikorska, A., Szabla, N., *et al.* (2021). Concerted evolution of body mass, cell size and metabolic rate among carabid beetles. *J Insect Physiol*, 132, 104272.

Shipley, B. (2000a). A correction note on a new inferential test for path models based on directed acyclic graphs. *Structural Equation Modeling*, 16, 537–538.

Shipley, B. (2000b). *Cause and correlation in biology. A user’s guide to path analysis, structural equations and causal inference*. Cambridge University Press, Cambridge.

Silvestro, D., Kostikova, A., Litsios, G., Pearman, P.B. & Salamin, N. (2015). Measurement errors should always be incorporated in phylogenetic comparative analysis. *Methods Ecol Evol*, 6, 340–346.

Smaers, J.B. & Mongle, C.S. (2018). Evomap: R package for the evolutionary mapping of continuous traits.

Smaers, J.B. & Rohlf, F.J. (2016). Testing species’ deviation from allometric predictions using the phylogenetic regression. *Evolution (N Y)*, 70, 1145–1149.

Sokal, R.R. & Rohlf, J. (2009). *Introduction to biostatistics*. Second. Dover Publications.

Stevenson, R.D., Hill, M.F. & Bryant, P.J. (1995). Organ and cell allometry in Hawaiian Drosophila: How to make a big fly. *Proc R Soc Lond B Biol Sci*, 259, 105–110.

Stone, M. (1974). Cross-validatory choice and assessment of statistical predictions. *J R Stat Soc Series B Stat Methodol*, 36, 111–133.

Svensson, E.I., Willink, B., Duryea, M.C. & Lancaster, L.T. (2020). Temperature drives pre‐reproductive selection and shapes the biogeography of a female polymorphism. *Ecol Lett*, 23, 149–159.

Symonds, M.R.E. & Elgar, M.A. (2002). Phylogeny affects estimation of metabolic scaling in mammals. *Evolution (N Y)*, 56, 2330–2333.

Tonini, J.F.R., Provete, D.B., Maciel, N.M., Morais, A.R., Goutte, S., Toledo, L.F., *et al.* (2020). Allometric escape from acoustic constraints is rare for frog calls. *Ecol Evol*, 10, 3686–3695.

Uyeda, J.C. & Harmon, L.J. (2014). A novel Bayesian method for inferring and interpreting the dynamics of adaptive landscapes from phylogenetic comparative data. *Syst Biol*, 63, 902–918.

Uyeda, J.C., J., E. & Harmon, L.J. (2020). bayou: Bayesian fitting of Ornstein-Uhlenbeck models to phylogenies.

Uyeda, J.C., Pennell, M.W., Miller, E.T., Maia, R. & McClain, C.R. (2017). The evolution of energetic scaling across the vertebrate tree of life. *Am Nat*, 190, 185–199.

Waller, J.T. & Svensson, E.I. (2017). Body size evolution in an old insect order: No evidence for Cope’s Rule in spite of fitness benefits of large size. *Evolution (N Y)*, 71, 2178–2193.

Waller, J.T., Willink, B., Tschol, M. & Svensson, E.I. (2019). The odonate phenotypic database, a new open data resource for comparative studies of an old insect order. *Sci Data*, 6, 1–6.

West, G.B., Brown, J.H. & Enquist, B.J. (1997). A general model for the origin of allometric scaling laws in biology. *Science (1979)*, 276, 122–126.

West, G.B., Brown, J.H., Enquist, B.J., West, G.B., Brown, J.H. & Enquist, B.J. (1999). The fourth dimension of life. Fractal geometry and allometric scaling of organisms. *Science (1979)*, 284, 1677–1679.

White, C.R. (2011). Allometric estimation of metabolic rates in animals. *Comparative Biochemistry and Physiology - A Molecular and Integrative Physiology*, 158, 346–357.

White, C.R., Marshall, D.J., Alton, L.A., Arnold, P.A., Beaman, J.E., Bywater, C.L., *et al.* (2019). The origin and maintenance of metabolic allometry in animals. *Nat Ecol Evol*, 3, 598–603.

White, C.R. & Seymour, R.S. (2003). Mammalian basal metabolic rate is proportional to body mass 2/3. *Proc Natl Acad Sci U S A*, 100, 4046–4049.

White, C.R. & Seymour, R.S. (2005). Allometric scaling of mammalian metabolism. *Journal of Experimental Biology*, 208, 1611–1619.

Wickham, H. (2016). *ggplot2: Elegant graphics for data analysis*. Springer Verlag, New York.

Wickham, H., Averick, M., Bryan, J., Chang, W., McGowan, L., François, R., *et al.* (2019). Welcome to the Tidyverse. *J Open Source Softw*, 4, 1686.

Wickham, H., François, R., Henry, L., Müller, K. & Vaughan, D. (2023). dplyr: A grammar of data manipulation.
